# Essential gene prediction using limited gene essentiality information–An integrative semi-supervised machine learning strategy

**DOI:** 10.1371/journal.pone.0242943

**Published:** 2020-11-30

**Authors:** Sutanu Nandi, Piyali Ganguli, Ram Rup Sarkar

**Affiliations:** 1 Chemical Engineering and Process Development, CSIR-National Chemical Laboratory, Pune, Maharashtra, India; 2 Academy of Scientific & Innovative Research (AcSIR), Ghaziabad, India; Torrens University Australia, AUSTRALIA

## Abstract

Essential gene prediction helps to find minimal genes indispensable for the survival of any organism. Machine learning (ML) algorithms have been useful for the prediction of gene essentiality. However, currently available ML pipelines perform poorly for organisms with limited experimental data. The objective is the development of a new ML pipeline to help in the annotation of essential genes of less explored disease-causing organisms for which minimal experimental data is available. The proposed strategy combines unsupervised feature selection technique, dimension reduction using the Kamada-Kawai algorithm, and semi-supervised ML algorithm employing Laplacian Support Vector Machine (LapSVM) for prediction of essential and non-essential genes from genome-scale metabolic networks using very limited labeled dataset. A novel scoring technique, Semi-Supervised Model Selection Score, equivalent to area under the ROC curve (auROC), has been proposed for the selection of the best model when supervised performance metrics calculation is difficult due to lack of data. The unsupervised feature selection followed by dimension reduction helped to observe a distinct circular pattern in the clustering of essential and non-essential genes. LapSVM then created a curve that dissected this circle for the classification and prediction of essential genes with high accuracy (auROC > 0.85) even with 1% labeled data for model training. After successful validation of this ML pipeline on both Eukaryotes and Prokaryotes that show high accuracy even when the labeled dataset is very limited, this strategy is used for the prediction of essential genes of organisms with inadequate experimentally known data, such as *Leishmania sp*. Using a graph-based semi-supervised machine learning scheme, a novel integrative approach has been proposed for essential gene prediction that shows universality in application to both Prokaryotes and Eukaryotes with limited labeled data. The essential genes predicted using the pipeline provide an important lead for the prediction of gene essentiality and identification of novel therapeutic targets for antibiotic and vaccine development against disease-causing parasites.

## 1. Introduction

Gene essentiality information of disease-causing organisms that throws light on the minimally essential genes that are absolutely required for the survival of the organism under any environmental condition has not only been indispensable for the prediction of novel therapeutic targets for antibiotic and vaccine development but has also contributed towards industrial bioprocessing, food microbiology, and bioremediation. However, experimental techniques [1–5] like genetic foot-printing, gene knockouts, RNA interference (RNAi), transposon mutagenesis have been employed to perform a genome-wide screen to check for gene essentiality are expensive, labor-intensive, as well as time-consuming.

As an efficient alternative to these highly complex experimental strategies, researchers now are employing computational techniques based on homology mapping, constraint-based modeling strategies, and machine learning strategies [6–8]. The homology-based essential gene prediction methods rely on the fact that essential genes are less likely to evolve, tend to remain conserved, and are often shared by distantly related organisms. Essential genes have been identified by comparative genomic analysis in different bacterial species such as Mycoplasma [9], *Liberibacter* [10], *Plasmodium falciparum* [11], and *Brucella spp*. [12]. However, the limitation of this method is that the conserved ortholog genes between different species form only a small fraction of the entire genome [13]. Also, it has been observed that highly conserved genes across different species are not always essential, as gene essentiality also depends on different environmental conditions where the organism resides.

Constraint-based modeling strategies, such as Flux Balance Analysis (FBA), employ genome-scale reconstructed metabolic networks to predict the metabolic fluxes at steady-state. This methodology is widely used for predicting essential genes by performing *in-silico* knockout of a gene and estimating its corresponding lethality [14–16]. A limitation of this FBA method is that only a limited number of environmental conditions can be considered for a certain biomass equation (or objective function) with respect to gene essentiality.

On the other hand, Machine Learning (ML) strategies comprise various data-driven approaches that train a model from the inherent patterns of the training data and make a prediction for the unlabeled data. These ML algorithms can be broadly grouped under supervised, semi-supervised, and unsupervised strategies [17,18]. The supervised strategies such as Decision Tree, Naïve Bayes, Support Vector Machine (SVM), etc. require sufficient amounts of labeled data for model training. In contrast, the unsupervised method relies on clustering algorithms (e.g., K-Means Clustering), where no labeled data is required. The semi-supervised ML algorithms that comprise Generative Models, Self-Training, Transductive SVM, and Laplacian SVM combine the potential of both supervised and unsupervised ML strategies and can train the model with a very limited amount of labeled data. At the same time, optimization of the hyper-parameter is crucial for enhancing the predictive performance of these machine learning classifiers. Various meta-heuristic techniques, such as Particle Swarm Optimization (PSO) [19], Genetic Algorithm (GA) [20], Ant Colony Optimization (ACO) [21], Grey Wolf Optimizer (GWO) [22], Ant Lion Optimizer (ALO) [23], etc. have been used for hyper-parameter tuning.

Based on the availability of labeled data of essential genes, researchers have employed supervised machine learning strategies [6–8] as well as deep learning-based strategies to predict essential genes [24,25]. The key advantage of these strategies lies in the fact that these models are capable of capturing the inherent patterns of a large array of biologically relevant ‘features’ that are distinctive and reflect the heterogeneous properties of essential genes. Supervised machine learning classifiers such as logistic regression [26,27], support vector machine [28–31], random forest [32], decision tree [26], ensemble [26] and probabilistic Bayesian-based methods [26,27,33] and instance-based learning methods such as K Nearest neighbor (K-NN) and Weighted KNN (WKNN) [34] have been used for gene essentiality prediction. Deep Learning strategies based on multilayer perceptron networks have also been used for essential gene prediction [24,35]. In these studies, researchers have mostly opted for simpler optimization methods for parameter tuning, such as the grid search technique, where the entire parameter space is explored in all possible combinations.

These machine learning-based classifiers predict gene essentiality of unannotated genes based on the pattern of the features of previously annotated genes that have been verified experimentally and labeled as essential and non-essential. In order to achieve this, researchers have curated different combinations of features. Most of the machine learning approaches use calculated features either from coding sequences [36–38] or network (e.g., protein interaction network, metabolic network) topological features [6,39] or both. Features, such as amino acid frequency and protein length computed from protein sequence, and codon adaptation index (CAI), Effective Number of Codons (ENC), Phyletic Retention (PR), GC content computed from nucleotide sequence are some of the known features of gene essentiality across bacteria [28,29,40]. Protein interaction networks (PIN) have been used to calculate topological network features to classify gene essentiality [28,39]. However, these strategies fail for many organisms that do not hold the idea of the centrality-lethality hypothesis in a PIN [41]. On the other hand, few studies have used flux-based features derived from metabolic networks to classify genes [29,30] that have been calculated under a single environmental condition that does not represent a universal set of features. Detailed reviews of the existing machine learning strategies for gene essentiality prediction have been discussed in different works of literature [6–8].

A major drawback of these existing machine learning algorithms for essential gene prediction is that they require a large amount of these labeled data that helps to train these models for an accurate prediction of the essentiality of unannotated genes, and show very poor performance when the labeled data set is imbalanced or limited. To circumvent these problems, in our previous study, an integrative machine learning strategy has been developed using a combination of feature selection algorithm, Support Vector Machine- Recursive Feature Elimination (SVM-RFE) [42] and classifier, Sequential Minimal Optimization (SMO) [43] for gene essentiality prediction in the metabolism of *Escherichia coli*, which performed well on imbalanced data set with diverse features computed from flux coupled connected sub-network along with other sequence-based features [40]. Here, the advantages of using the Flux Coupling Analysis (FCA) based feature for the prediction of gene essentiality with high accuracy and confidence have been reported. FCA analysis help to capture the physiological dependence of one gene-reaction combination on another, which is coupled to it, under all input exchanges of a reaction, representing all possible environmental conditions, thereby helping the classifier to accurately identify the minimally essential genes that are absolutely crucial for sustaining the metabolic demands of the cell to ensure its survival [40]. However, this technique was unable to predict gene essentiality when a very small amount of experimentally verified labeled data are available.

To mitigate the problems inherent in the existing strategies, we propose an integrative semi-supervised machine learning strategy based on Laplacian SVM [44] for the classification of genes using gene sequence, protein sequence, network topological, and flux-based features with very limited labeled data on gene essentiality of metabolic networks for both Prokaryotic and Eukaryotic organisms. Another objective of this work is the development of a new machine learning pipeline to help in the annotation of essential genes of less explored organisms, like *Leishmania donovani* and *Leishmania major*, the causative organisms for the neglected tropical disease Leishmaniasis, for which very limited experimental data is available. By using the available tools and techniques, the prediction of gene essentiality and targeted therapy for the disease becomes extremely difficult [45]. In the present work, it is hypothesized that using these diverse features, like topological network features of both the genome-scale metabolic reaction network as well as the flux-coupled sub-networks, together with the sequence-based features simultaneously, that can capture both the properties of genotype and phenotype and by employing the proposed algorithm, it is possible to predict the essentiality of uncharacterized genes with high accuracy even in the cases where labeled data is limited. This is in contrast to other machine learning pipelines for essential gene prediction that relies on only sequence-based features and has been applied to only Prokaryotes [26,46]. In this work, the novel features derived from the genome-scale metabolic reaction network, as well as the flux-coupled sub-networks, contribute towards the better prediction of gene essentiality by capturing the contribution of a gene in sustaining the metabolic demands of the cell under varied environmental challenges that are indispensable for its survival. A new scoring technique has also been proposed, called the Semi-Supervised Model Selection Score (SSMSS) that correlates well with Mathews Correlation Coefficient (MCC) [47] and can be used for the selection of the best model when the calculation of supervised performance metrics like MCC or auROC is difficult due to lack of experimental data. After the successful validation of this proposed pipeline on twelve organisms, with well-annotated genes essentiality information, using as low as 1% labeled data on two types of training datasets (i.e., with 80% training and 20% blind datasets, as well as using the whole dataset for training), the essential genes in *Leishmania* have been predicted as well as categorized the reaction-gene pairs in five different groups based Gene-Protein-Reaction (GPR) association in metabolism. These groups depict the association of the reactions with different combinations of essential and non-essential genes, which throws light on the probable reaction-gene combination that can be used for targeted therapy. This study promises to lay the foundation to the prediction of gene essentiality information for less explored organisms that will help experimental biologists to identify novel therapeutic targets even when only limited information is available.

## 2. Methods

**Fig 1:**
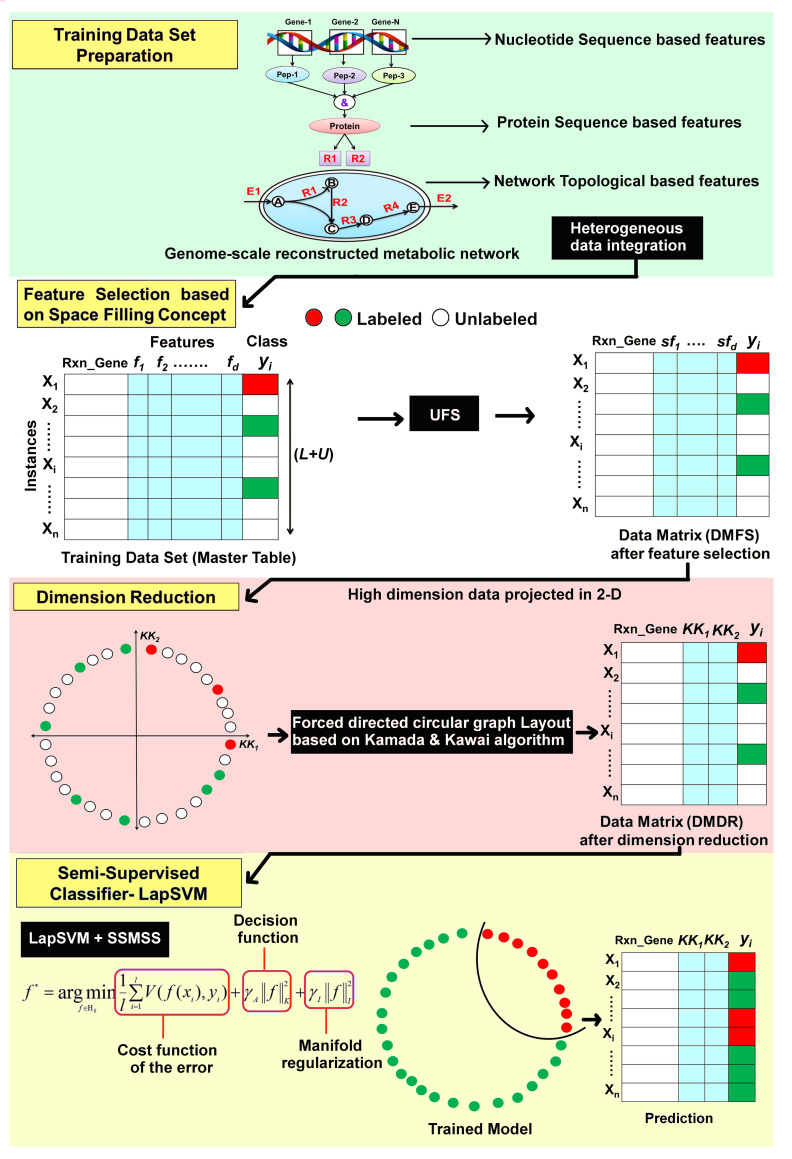
The proposed machine learning strategy. The integrated pipeline for prediction of essential genes based on limited labeled training dataset consisting of reaction-gene pairs with sequence, informatics, and topological network features.

The Machine learning strategy developed to predict gene essentiality, as elucidated in Fig 1, combines feature selection technique based on a space-filling concept, dimension reduction (DR) using the Kamada-Kawai (KK) algorithm, and classification of genes based on a semi-supervised machine learning algorithm employing Laplacian Support Vector Machine (LapSVM). This pipeline combines heterogeneous biological features, such as sequence-based, as well as network-based features. It classifies genes based on a training dataset of very limited information of essential genes from experimental data. Twelve organisms comprising of both Prokaryotes and Eukaryotes (Table 1) with well-annotated genes essentiality information from the OGEE database [48] have been considered for the validation of this proposed strategy, and the subsequent prediction of essential genes in *Leishmania major* and *Leishmania donovani* have been performed. The gene essentiality information has only been considered from the OGEE database as this collates data using text mining as well as manually verified with experimental data, unlike other gene essentiality databases that rely on only text mining.

**Table 1:** Organisms considered for model training and validation.

Organism Name	Abbreviation	Input files	
		FASTA files of coding nucleotide and protein sequence (RefSeq assembly accession)	Genome-Scale Reconstructed Metabolic Network
**Organisms used for Model Development and Validation of the Proposed Pipeline**			
*Acinetobacter sp*. ADP1	ACIAD	GCF_000046845.1_ASM4684v1	iAbaylyiv4 [49]
*Bacillus subtilis subsp*. *subtilis str*. 168	BACSU	GCF_000009045.1_ASM904v1	iYO844 [50]
*Escherichia coli* K-12 MG1655	ECOLI	GCF_000005845.2_ASM584v2	iJO1366 [51]
*Helicobacter pylori*	HELPY	GCF_000008525.1_ASM852v1	iIT341 [52]
*Mycobacterium tuberculosis* H37Rv	MYCTU	GCF_000195955.2_ASM19595v2	iNJ661 [53]
*Pseudomonas aeruginosa* PAO1	PSEAE	GCF_000006765.1_ASM676v1	iPae1146 [54]
*Pseudomonas aeruginosa* UCBPP-PA14	PSEAB	GCF_000014625.1_ASM1462v1	iPau1129 [54]
*Salmonella enterica subsp*. *enterica serovar Typhimurium* LT2	SALTY	GCF_000006945.2_ASM694v2	STM_v1_0 [55]
*Staphylococcus aureus subsp*. *aureus* NCTC 8325	STAAB	GCF_000013425.1_ASM1342v1	BMID000000141098 [56]
*Saccharomyces cerevisiae*	YEAST	GCF_000146045.2_R64	iMM904 [57]
*Caenorhabditis elegans*	CELEG	GCF_000002985.6_WBcel235	iCEL1273 [58]
*Mus musculus*	MUSMU	GCF_000001635.26_GRCm38.p6	iMM1415 [59]
**Organisms used for Case Study**			
*Leishmania donovani*	LDONO	TriTrypDB-36	iMS604 [60]
*Leishmania major*	LMAFR	TriTrypDB-36	iAC560 [61]

### 2.1. Training data and Testing data set preparation and integration of heterogeneous features

The training datasets for the pipeline of the 12 target organisms were prepared by calculating mainly two types of features: topological features and sequenced based features. These features were extracted primarily from the genome-scale reconstructed metabolic networks, the fasta files containing the coding nucleotide sequences of the genes, and protein sequences of these target organisms (Table 1) [62]. From the genome-scale reconstructed metabolic network, the information of metabolites, reactions, and genes was collated.

The sequence-based features and the topological features of the metabolic reaction network, and flux-coupled sub-network based were calculated and accumulated for each reaction-gene combination. These reaction-gene combinations integrate diverse features of the metabolic adaptation of the organism and give detailed insights into the role of a particular gene in the metabolic reaction network. This helps in the prediction of the essentiality of the gene in the target organism with high accuracy. A total of 289 features were computed for each reaction-gene pair. Brief descriptions of these features are given below, and their abbreviation**s** are enlisted in S1 Table.

To establish the model consistency and reproducibility of the proposed pipeline, two different types of data sets for each of the twelve organisms have been used. The first type of data set consists of 80% data points of total data set with limited labeled data that is used for training while the remaining 20% is used for blind testing to check the model validation. Using this 80% data points of the whole dataset, different types of training data set are further created with limited labeled data points in the range, i.e., i % Labeled (L) and (100—i%) Unlabeled (UL) data, where i = 1, 2, 3, 4, 5, 10, 30, 50, 70 and 90. In each category, labeled samples were chosen randomly from the master table. It is to be mentioned here that this selection of labeled data was conditionally randomized to ensure that both the essential and non-essential genes categories appear with equal probability. In this way, 100 data sets in each labeled category have been created.

The second type of data set consists of the whole dataset with limited labeled data, which is used for model training and prediction purposes for each of the twelve organisms. It is to be mentioned here that, in less-studied organisms where gene essentiality information is very less, a blind test cannot be applied. For those cases, the whole data set with limited labeled data will be used for model training and prediction purposes.

#### Topological analysis of reaction and flux-coupled sub-network

The metabolic network of each target organism was transformed into an undirected reaction network (RN), in which each node denotes an enzyme (reaction), and each edge represents the connection between two reactions that have common metabolites. The commonly used topological network features, such as centrality measures, that highlight the biological significance of an enzyme in a network were computed [63]. Generally, a central and highly connected enzyme in biological networks is often essential as it represents an important hub within the network [64]. If this hub node is blocked, then the whole pathway might be disrupted.

Similarly, Flux coupling analysis (FCA) is an optimization procedure based on flux, which represents whether the reaction subsets are coupled or not in certain given specific environmental exchange constraints [65,66]. Flux-coupled subgraph was used to extract biologically relevant topological features dependent on physiological flux relationships.

Eight centrality measures have been computed for both the reaction as well as the flux coupled networks, *viz*., Degree Centrality, Eigenvalue Centrality, Eccentricity, Hub score, Authority Scores, Page Rank, Betweenness Centrality, and Number of triangles. A detailed description of all these centrality measures has been discussed in different literature [67–69]. These topological features have been calculated using the “igraph” package in R [70].

#### Features derived from the coding nucleotide sequence

Three types of features (*viz*. nucleotide content, codon usage bias, and information-theoretic features) of the metabolic genes have been extracted from the nucleotide sequence of the organisms that contribute towards gene essentiality. A brief description of the features has been discussed below.

#### Nucleotide content

Previous studies have elucidated that in bacterial genomes, GC content is correlated with the environmental condition in which the bacterium survives [71]. Hence, the related GC content of the genome of a target organism can be an essential feature for gene essentiality prediction. Another study showed that there is a significant difference in the distribution of the frequency of occurrence of A, T, G, and C nucleotides at the 3rd synonymous position of codons between the essential and non-essential genes [40]. These features were computed using an in-house code.

#### Codon usage bias

Protein abundance in an organism can be predicted by using Codon usage [72–74]. Highly expressing abundant proteins in metabolism might have functional importance and can be essential. Codon usage bias features, like Effective Number of Codons (ENC) [75] and Codon Adaptation Index (CAI) [73], were calculated using EMBOSS package version 6.6.0–1 [76].

#### Mutual Information (MI) and Conditional Mutual Information (CMI)

A previous study has used information-theoretic features such as mutual information (MI) and conditional mutual information (CMI), for essential gene prediction [37]. MI and CMI profile of coding nucleotide sequence can be used as genomic signatures which represent the phylogenetic relationship between genomic sequences [77]. A total of 80 features (16 MI and 64 CMI) have been computed by using in house Perl script.

#### Features derived from protein sequence

In order to investigate the dependence of gene essentiality on protein sequences, various derived and informatic features such as the frequencies of the amino acids, protein length, paralogy score, average Kidera factor, etc. have been considered in this study.

#### Frequencies of the twenty amino acids and protein length

Each protein sequence related to the reaction-gene combination was used to calculate the occurrences of the 20 amino acids that reflect the physicochemical properties of these proteins related to each of the reaction-gene combinations under consideration. These twenty features were calculated using EMBOSS package version 6.6.0–1 [76] and named according to their corresponding 20 amino acids.

#### Paralogy based features (paralogy score)

The sequence similarity of a gene in its intragenome is called a paralogous gene of an organism. Paralogous genes have the same or similar types of biological functions. An organism may not be affected by the deletion of one of the paralogous genes because another paralogous gene may compensate for a similar type of function. So there are fewer chances for paralogous genes to be essential [78].

The paralogy score of a gene was calculated by performing a BLAST [version 2.2.26] search against the whole set of protein sequences of a target organism with different E-value threshold ranging from 10^−3^ to 10^−30^ with at least 40% identity. Features based on paralogy score were labeled as P3 (E-value cut off 10^−3^), P5 (E-value cut off 10^−5^), P7 (E-value cut off 10^−7^), P10 (E-value cut off 10^−10^), P20 (E-value cut off 10^−20^), P30 (E-value cut off 10^−30^). These features have been calculated using in house Perl script.

#### Fourier sine and cosine coefficient

The Fourier sine and cosine coefficient of protein sequences [79] have been used to see if there are any inherent patterns which will help to classify between essential and non-essential genes. The Fourier coefficient (FC) is the converted numerical values of protein sequences, which describes the physical properties of corresponding amino acids. These physical properties represent the ten property factors using factor analysis introduced by Kidera et al. [80]. Mathematical representations of these coefficients are given below:

$${FC\sin WN_{k}\_KF_{n}=a}_{k}^{\left[n\right]}=\sum_{l=0}^{N-1}f_{l}^{\left[n\right]}\sin\left(\frac{2\pi kl}{N}\right)$$ (Eq 1)

$${FC\cos WN_{k}\_KF_{n}=b}_{k}^{\left[n\right]}=\sum_{l=0}^{N-1}f_{l}^{\left[n\right]}\cos\left(\frac{2\pi kl}{N}\right)$$ (Eq 2)

Where the length of the protein sequence is N, $f_{l}^{\left[n\right]}$ is n^th^ property factor of amino acid *l*, and wavenumber is *k* (Eqs 1 and 2).

Fourier sine and cosine coefficient in a specific range of Wave Number (WN) and Kidera Factor (KF) was calculated. The range of WN and KF are 0≤*k*≤7 and 1≤*n*≤10. It is also reported that global folding information of the protein is encoded in a specific range of wavenumber 0≤*k*≤7 [79]. A total of 150 features were computed. These features have been calculated using in house Perl script.

#### Average Kidera factor

The ten Kidera Factors (viz. KF1: Helix/bend preference, KF2: Side-chain size, KF3: Extended structure preference, KF4: Hydrophobicity, KF5: Double-bend preference, KF6: Partial specific volume, KF7: Flat extended preference, KF8: Occurrence in the alpha region, KF9: pK-C, KF10: Surrounding hydrophobicity) were derived by multivariate analysis on 20 amino acids using 188 physical properties and dimension reduction techniques [80]. The protein sequence of the corresponding reaction-gene combination was used to calculate ten features (*AKF*_*i*_ where, i = 1 to 10) by averaging the ten Kidera factors. These features have been calculated using in house Perl script.

### 2.2. Feature selection based on the space-filling concept

The contribution of these 289 features towards gene essentiality is unknown; hence, there may be a possibility to select redundant features by the feature selection algorithm. These redundant features may affect the training performance of the machine learning model. Hence, it is important as well as challenging to choose the non-redundant, unique feature subset for training the model. Feature selection helps to capture the most relevant biological features and helps the classifier to learn a better way to predict essential and non-essential genes with high accuracy. Here the unsupervised feature selection method based on the space-filling concept has been used [81]. This unsupervised method selects the features based on a coverage measure that estimates the spatial distribution of the data points in a hypercube and ensures uniform distribution of points in a regular grid in the data space. The method captures the variability of features with new and relevant information about the data. This method has been tested on various datasets and different scenarios with noise injection and data shuffling. The benefits of using this algorithm are two folds. Firstly, being an unsupervised algorithm, prior information of the output variable is not required.

Additionally, here no classifier is required for feature selection. Hence time complexity is less in comparison to other feature selection algorithms, like SVM-RFE. Also, it has been observed that this method gives better information of relevant features than other unsupervised correlation-based feature selection techniques that, although it can remove the redundant features, cannot eliminate the features with low variability that are non-relevant and non-informative for classification [82,83].

### 2.3. Dimension reduction using forced directed graph layout

After feature selection, the data set was transformed into a lower dimension (2-D) using a dimension reduction technique for visualization. Projected 2-D features set to reserve all the information the same as higher-dimensional data. This is an important step in the pipeline as the classifier works better in 2-D than with the higher dimension data. For dimension reduction, a force-directed graph layout algorithm Kamada-Kawai has been used that considers each data point as a node in a graph having attractive and repulsive forces between them that can be modeled as springs connecting the nodes [84]. The algorithm then tries to cluster the data points by minimizing the total energy of the system based on attracting and repelling forces between them. Here the input of the Kamada-Kawai algorithm is a graph constructed by using the K-Nearest Neighbour (K-NN) algorithm. For known organisms, it has been observed that essential genes are clustered together in one side of an arc in a circle layout, and non-essential genes are clustered in the rest of the circle. A circular layout of each organism has been observed from the Kamada Kawai algorithm with a specific parameter (K Nearest Neighbor) value of the K-NN algorithm. Here it is assumed that if a similar circular layout is observed for less explored organisms related to gene essentiality, the unlabeled genes will be clustered together category wise and reside on the arc of the circle. This analysis had been performed using the “dimRed” package in R [85].

Both the feature selection and the dimensionality reduction methods are used for not only reducing the number of features in a dataset but also to select the important features, which are contributing significantly. Feature selection is used for selecting the relevant features without changing the original values, whereas, the dimensionality reduction step transforms the higher dimensional features into a lower dimension. From the dimension reduction technique it is very difficult to identify the key features which are contributing for classifications, hence the feature selection step is necessary.

To test the efficiency of this dimension reduction technique combined with unsupervised feature selection and LapSVM classifier, the performance metrics of Kamada-Kawai has been compared against other dimension reduction techniques, such as Principal Component Analysis (PCA) [86], Metric Dimensional Scaling (MDS) [87], Fruchterman Reingold [88] and FastICA [89] using the gold standard dataset of twelve organisms. To test the statistical significance of the results, the one-tailed Mann Whitney U Test has been performed with 1% level of significance (*P*<0.01).

### 2.4. Semi-supervised classifier: Laplacian SVM

Essential gene classification using the machine learning technique can be a difficult task when a minimal amount of gene essentiality information for the target organism is available. In this setting, semi-supervised learning is an appropriate approach that builds a trained model from labeled and unlabeled samples [90]. Most of these semi-supervised algorithms follow two common assumptions, i.e., cluster assumption and manifold assumption. Cluster assumption states that data points in the same cluster have a chance of having the same class label. Manifold assumption means that close data points along the manifold area follow similar data structures or similar class labels. However, cluster assumption follows the global feature, and manifold assumption follows the local features in the model.

Laplacian support vector machine (LapSVM) is a graph-based semi-supervised learning method, which is based on a manifold regularization framework [44]. The graph is constructed from labeled and unlabeled data as the node. The similarity between data points in a graph can be assigned by edge weight, which is calculated from the K-NN algorithm. In this way, the information of labeled data points can be passed to another node, and then, the unlabeled nodes can be labeled. The input data set being circular (non-linear), Radial Basis Function (RBF) kernel with the classifier LapSVM have been used. This analysis had been performed using the “RSSL” package in R [91].

### 2.5. The score for best model selection

There are various performance metrics, e.g., True Positive Rate (TPR), False Positive Rate (FPR), precision, recall, F-measure, Matthews correlation coefficient (MCC), Area under the receiver operating characteristic curve (auROC), etc. to evaluate the trained model in supervised machine learning technique. These measures are statistically significant if sufficient labeled data are available. However, due to limited labeled data, these metrics will not work for best model selection in a semi-supervised type algorithm. To circumvent the above problem, a new measure has been proposed, called the Semi-Supervised Model Selection Score (SSMSS), for selecting the best model. This SSMSS score is dependent on four different measurements (Eq 3). For this, the training data set, having limited labeled reference, has been labeled as ground truth (GT) reference. Another reference set called the pseudo reference (PR) has been considered by calculating the distance from unlabeled data points to the labeled dataset. The dataset containing the predicted labels by the Laplacian SVM classifier has been labeled as the Laplacian Reference (LR). Thereafter, Silhouette Index (SI) [92] was computed to check the clustering grouping quality. The CorrectPrediction_GT_LR_ measure was calculated based on the matches between the predictions of the Laplacian SVM classifier with the Ground Truth data. Here, the calculation of the MCC with the help of Pseudo-reference and Laplacian Reference was represented as MCC_PR_LR_. Silhouette Index calculation based on Pseudo Reference and Laplacian Reference was denoted by SI_PR_ and SI_LR_ respectively. Based on these parameters, the values of the proposed Semi-Supervised Model Selection Score (SSMSS) may vary from 0 to 1. If any of the above four measurements is low, then the SSMSS value will be drastically decreased. The best model will be selected from 64 models which has the highest SSMSS value for each data set in different parameters combinations, i.e., kernel parameter [Radial Basis Function (RBF) kernel parameter sigma (*σ*)] and LapSVM parameters [lambda (*λ*): L_2_ regularization parameter and gamma (*γ*): the weight of the unlabeled data]. It may be mentioned here that the score will not consider those models which have negative Silhouette Index and MCC value. The parameters (*σ*,*λ*,*γ*) have been varied with four different values, i.e., 0.01,0.1,1,10. Therefore, by tuning these model parameters using grid search, 64 models for each data set have been generated. The following equation has been proposed for the calculation of the SSMSS.

$${\mathrm{SSMSS}}_{k=1\;\mathrm{to}\;64}=\min\left\{{\mathrm{CorrectPrediction}}_{\mathrm{GT}\_\mathrm{LR}}^{k}{,\mathrm{MCC}}_{\mathrm{PR}\_\mathrm{LR}}^{k}{,\mathrm{SI}}_{\mathrm{PR}}^{k}{,\mathrm{SI}}_{\mathrm{LR}}^{k}\right\}$$ (Eq 3)

$$\forall {\mathrm{MCC}}_{\mathrm{PR}\_\mathrm{LR}}^{k}\geq 0,{\mathrm{SI}}_{\mathrm{PR}}^{k}\geq 0,{\mathrm{SI}}_{{}_{\mathrm{LR}}}^{k}\geq 0.$$

$${\mathrm{SSMSS}}_{\mathrm{best}}=\max\left\{{\mathrm{SSMSS}}_{k=1},{\mathrm{SSMSS}}_{k=2},\ldots \ldots \ldots ,{\mathrm{SSMSS}}_{k=64}\right\},$$

where k is the k^th^ model with a particular parametric combination and SSMSS_best_ is the best score of the best model among these 64 models.

### 2.6. Time complexity of the proposed strategy

The proposed pipeline has three components (i.e., Unsupervised Feature Selection, Kamada Kawai Dimension Reduction Technique, and LapSVM semi-supervised classifiers), which work sequentially. To calculate the total time complexity T(n,d) of the proposed strategy, the cumulative effect of all three components have been considered, where n denotes the number of data points (reaction-gene pair) that depends on the size of the metabolic network of the organism, and d is the total number of features.

The time required for each of the three components can be represented as follows [44,81,84]:

Time required for Unsupervised Feature Selection algorithm $=\frac{d(d+1)n^{2}}{2}$ Time required for Kamada Kawai algorithm = *n*^3^

Time Required for LapSVM = *n*^3^

Therefore, the total time required T(n,d) can be represented as:

$$\begin{array}{l} ∴T(n,d)=\frac{d(d+1)n^{2}}{2}+n^{3}+n^{3}\\ or,\;T(n,d)\leq 4n^{3}+n^{2}(d^{2}+d)\\ or,\;T(n,d)\leq (4+d+d^{2})n^{3}\\ or,\;T(n,d)\leq Cd^{2}n^{3}\\ or,\;T(n,d)=Ο(d^{2}n^{3}) \end{array}$$

Where, *C* is a constant, in particular, *C*≥6 ∀d,n∈ℕ.

Therefore, the total time complexity of the proposed strategy is *O*(d^2^n^3^).

### 2.7. Gene essentiality prediction, experimental validation, and pathway enrichment

The essential gene prediction results for the twelve model organisms have been compared with experimental data obtained from the OGEE database, and the corresponding supervised performance metrics such as TPR, FPR, MCC, auROC, etc. were calculated. Further, the predicted essentiality information of the reaction-gene pairs of all twelve organisms has been categorized into five different groups based on their involvement in different reactions. These five groups are following: **CEN** (Combination of Essential and Non-essential), involving both essential and non-essential genes controlling a reaction; **ME** (Multiple Essential), multiple essential genes involved in a reaction; **MN** (Multiple Non-essential), multiple non-essential genes governed a reaction; **SE** (Single Essential), single essential genes involved in a reaction; **SN** (Single Non-essential), single non-essential genes involved in a reaction. Thereafter, the distributions of the five categories of reaction-gene pairs from the predicted results have been compared with the distribution observed in experimental data for all the organisms using the Chi-Square Test (1% level of significance).

For *Leishmania donovani* and *Leishmania major*, the best model was selected based on the SSMSS score for the prediction of the essential reaction-gene combinations. These predicted reaction gene combinations were then classified into the five categories, like the other twelve species. The list of unique genes that were extracted from this predicted essential reaction-gene pairs was analyzed for their associated Gene Ontology (GO) terms [93,94] from the Uniprot database [95]. The percentages of genes associated with each GO term were calculated for both the organisms. Additionally, using the DAVID pathways enrichment tool [96], the essential genes were further analyzed to identify the significantly enriched KEGG pathways [97] that were associated with these essential genes.

Source codes of the entire machine learning strategy and pipeline are given in S1 Text, which consists, Training data set preparation and integration of heterogeneous features, Feature selection based on the space-filling concept, Dimension reduction using forced directed graph layout, and Semi-supervised classifier: LapSVM.

## 3. Results

### 3.1. Model validation with experimental data

The integrative proposed strategy (Fig 1) was applied and validated on twelve organisms (Table 1) with well-annotated genes essentiality information from experimental data obtained from the OGEE database [48].

### 3.2. Features frequently selected by the feature selection algorithm

The important features chosen by the feature selection algorithm have been represented in the heat map (See methods section for a detailed description of features and S1 Fig), where X-axis represents the name of the 82 features that have been selected at least once by the features selection algorithm and Y-axis corresponds to names of the organism. Red cell color indicates features selected by the feature selection algorithm in the corresponding organism. White-colored cell shows the feature that is not selected or is redundant. Among 289 features, three features, *viz*., Reaction Network betweenness centrality (RN_betweenness), Reaction Network Page Rank centrality (RN_page_rank), and Flux Coupled Analysis Network Page Rank centrality (FCA_page_rank) are selected by the features selection algorithm for every organism. These frequently selected features are topological network features. Apart from these features, Information-theoretic features (Fourier sine or cosine coefficient, Mutual Information, Conditional Mutual Information) from nucleotide and peptide sequences are also selected. If a node is important in the reaction network and flux-coupled network, then there is a chance that the enzyme or protein which controls that particular reaction and its corresponding coding sequence is also essential.

### 3.3. Dimension reduction

After applying feature selection, the Kamada-Kawai dimension reduction technique [84] is used for visualization purposes. Here, a circular layout of each organism is observed. While the essential gene-reaction combinations are clustered together in one side of the arc in a 2-D circular layout, the non-essential reaction-gene combinations are clustered in the rest of the circle. Now on applying Laplacian SVM, the classifier was able to easily classify gene essentiality based on their transformed 2-D feature and the limited label information. Now in different parameter combinations of Laplacian SVM, different trained models are obtained. To select the best model among trained models, the proposed SSMSS score has been used.

### 3.4. Robustness of the proposed score (SSMSS)

To check the robustness of the SSMSS score, the proposed strategy has been applied on both types of training data set (i.e., data set with 80–20% combination of samples and with the whole data set) for these twelve organisms. Using this 80% data points of the whole dataset, different types of training data set is further created with limited labeled data points in the range, i.e., i % Labeled (L) and (100—i%) Unlabeled (UL) data, where i = 1, 2, 3, 4, 5, 10, 30, 50, 70 and 90. In each category, labeled samples were chosen randomly from the master table. It is to be mentioned here that this selection of labeled data was conditionally randomized to ensure that both the essential and non-essential genes categories appear with equal probability. In this way, 100 data sets in each labeled category have been created. For the testing purpose, both the whole training data set and the 20% blind data set have been used for prediction. The parameters (*σ*,*λ*,*γ*) were tuned with four different values i.e. 0.01,0.1,1,10. Therefore, by tuning these model parameters using grid search generated 64 models for each data set have been created. After that, the prediction results were compared with the known gene essentiality information, which is publicly available from the experiment. Six supervised performance metrics have been calculated for the predicted class label with the known class label. After that, the association between the proposed score and auROC was assessed. To verify the linear relationship between auROC and the proposed score (SSMSS), the Pearson correlation coefficient has been calculated, and scatter plots were generated in different limited labeled data sets in each target organism (S2 Fig).

From the scatter plot (S2 Fig), it has been observed that in all the cases, Pearson correlation >0.75. Hence, it may be inferred that due to the linear relationship existing between auROC and the proposed score (SSMSS), the applicability of this scoring technique is asserted and can be used for the calculation of the performance measurement matrix and best model selection for the semi-supervised based classifier.

### 3.5. Predictive performance of the best models in the different labeled category on training and blind test data set

**Fig 2:**
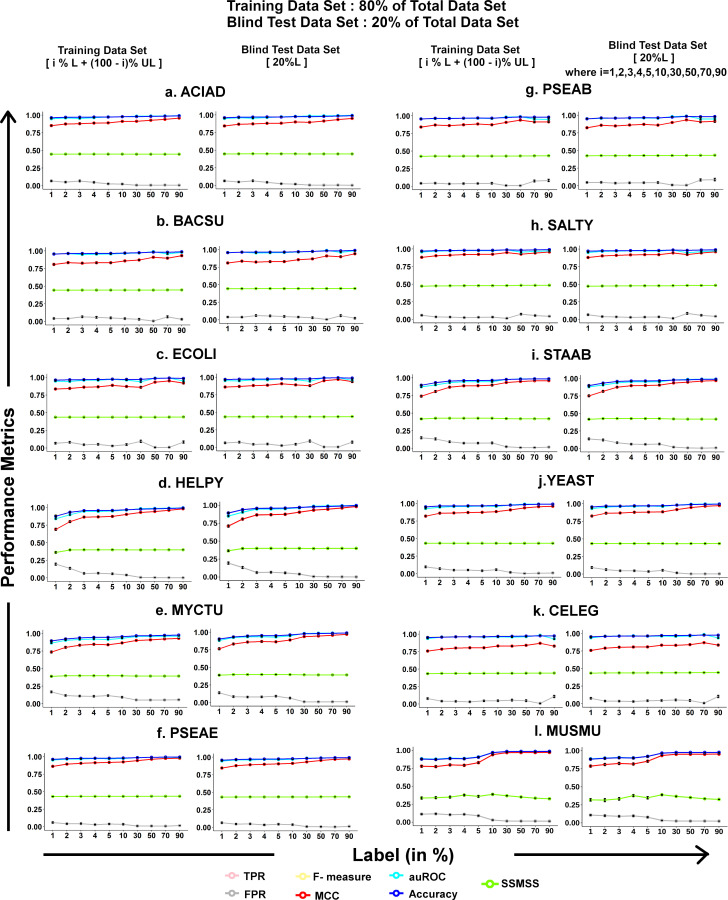
Comparison of the predictive performance of the best models in the different labeled category. The average performance of the best 100 models at training and blind testing for six supervised metrics (i.e., TPR, FPR, F-measure, MCC, auROC, accuracy) and SSMSS for each labeled type. The X-axis represents the category of labeled data, the Y-axis represents the value of performance metrics.

In a real-life scenario, only limited gene essentiality information is available for the less explored organisms. However, model building from this limited label data and determining how the highest score will select the best model is difficult. Hence, to test the model performance on known organisms by creating limited labeled datasets (i.e., by varying the limited labeled data from 1% to 90% from the 80% training dataset), six supervised performance metrics have been calculated for each category under different parameter combinations of *σ*,*λ*,*γ* (See Section 2.5: The score for best model selection) for a detailed description of these parameters). Here, within each labeled category, the average behavior of the predictive performance (six supervised performance metrics) and the Score (SSMSS) of the best 100 trained models are plotted in Fig 2. This has been shown for two different conditions, training data set (80% of the whole data) and blind testing data set (20% of the whole data). As observed from the low standard deviations for each metrics (under each category), it is worth to mention that the accuracy for the training and testing are very similar in most of the cases.

From these plots, it has been observed that the model selection based on the SSMSS score in each category corresponds to a high auROC value of greater than 0.8 in all cases across all organisms. Also, it is observed that if the label increases, then model performance will also show higher accuracy. However, it is seen that the auROC score remains consistently high, using 1% labeled data or more, which establishes the fact that the proposed method can predict using a minimum of 1% labeled data. It has also been observed that this method is giving a consistent better predictive performance on both Prokaryotic and Eukaryotic organisms for both the data sets (80% training and 20% blind testing) and follow similar patterns for six supervised performance metrics in differently labeled categories. As the predictive performance of 20%, the blind data set is similar to training performance, so further, it can be concluded that model overfitting and underfitting is not arising in this case.

To compare the predictive performance of the proposed method, 1% labeled data set has been considered for each of the twelve organisms. For training, different supervised classifiers have been used, such as Random Forest [98], Naive Bayes [99], Logistic regression [100], J48(C.45) Decision Tree [101] as well as our own previously reported Supervised essential gene prediction pipeline [40] on the whole dataset for testing (S3 Fig). In all of the cases, it is found that the proposed method performed better than all other methods using only 1% labeled data of the whole training dataset.

### 3.6. Effect of feature selection and dimension reduction in model performance

**Fig 3:**
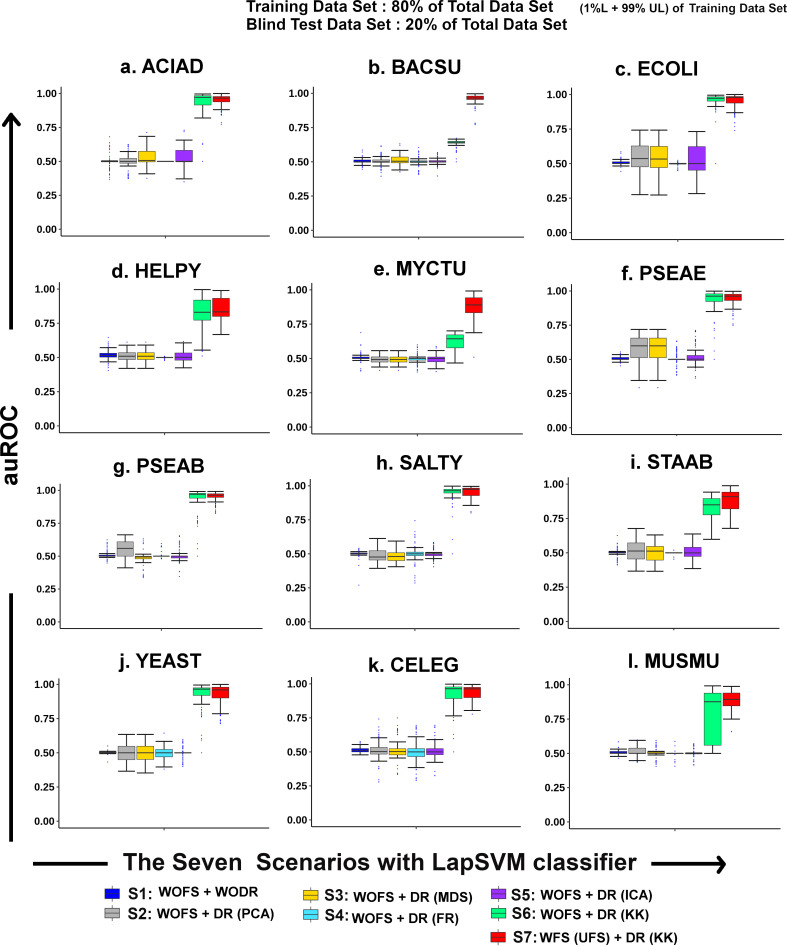
Effect of feature selection and dimension reduction on model performance. Comparison of the effect of different dimension reduction techniques PCA, MDS, FR, ICA, and KK (S2—S6) with S1 (Without Feature Selection and Without Dimension Reduction) and S7 (With Feature Selection and With Dimension Reduction-KK) when combined with LapSVM classifier. Plot represents the auROC value of 100 best models with 1% labeled data across all organisms.

To compare the effect of feature selection and dimension reduction steps along with the LapSVM classifier, seven different types of classification scenarios, based on different dimension reduction technique such as PCA, MDS, FR, ICA, and KK, were simulated on training data set (80% data points) and blind testing (20% data points) data sets of twelve organisms. The corresponding performance was calculated on the blind test data set (Fig 3). Each training data set has only 1% labeled data, and the rest of them Unlabeled.

The seven scenarios were created with LapSVM classifier and combinations of features selection and dimension reduction techniques:

Scenario 1 (S1): Without feature selection and Without dimension reduction technique [WOFS +WODR]
Scenario 2 (S2): Without feature selection and With dimension reduction technique (Principal Component Analysis) [WOFS + DR (PCA)]
Scenario 3 (S3): Without feature selection and With dimension reduction technique (Metric Dimensional Scaling) [WOFS + DR (MDS)]
Scenario 4 (S4): Without feature selection and With dimension reduction technique (Fruchterman Reingold) [WOFS + DR (FR)]
Scenario 5 (S5): Without feature selection and With dimension reduction technique (Independent Component Analysis) [WOFS + DR (ICA)]
Scenario 6 (S6): Without feature selection and With dimension reduction technique (Kamada Kawai) [WOFS + DR (KK)]
Scenario 7 (S7): With feature selection (Unsupervised Feature Selection) and With dimension reduction technique (Kamada Kawai) [WFS (UFS) + DR (KK)]

From this analysis, it has been observed that for scenarios 1 to 5, the auROC value is very low, which signifies that dimension reduction techniques, e.g., PCA, MDS, FR, ICA, cannot significantly improve the gene essentiality prediction (Fig 3). On the other hand, for scenarios 6 and 7, it is observed that on applying the Kamada-Kawai method of dimension reduction along with unsupervised feature selection, the model performance (auROC) improves drastically in each target organism. On comparing the efficacy of Kamada-Kawai (KK) with the other dimension reduction methods using the one-tailed Mann-Whitney U Test, a significant improvement in auROC values (*P*<0.01) for all the twelve organisms was observed (S2 Table). Scenario 6 highlights the importance of this dimension reduction step, where it is found that even without feature selection, the dimension reduction step [S6: WOFS + DR (KK)] has a huge impact on the results (*P*<0.01) [S3 Table]. However, the feature selection step helped us in identifying the minimal set of features that contribute towards gene essentiality prediction with greater accuracy in all organisms (lower *P*-values obtained in Scenario 7 with [S7: WFS + DR (KK)]) (S3 Table). Hence, it is observed that the Kamada-Kawai dimension reduction technique, when combined with LapSVM, gives significantly better performance for all twelve organisms even when only 1% labeled data is used (Fig 3).

### 3.7. Predictive performance using whole training data set

In model organisms where gene essentiality information is sufficiently available at the genome-scale, blind testing can be applied. However, in less explored organisms where gene essentiality information is very less, a blind test cannot be applied as the reference size is very small. For these cases, the whole data set with limited labeled data can be used for model training and prediction purposes.

**Fig 4:**
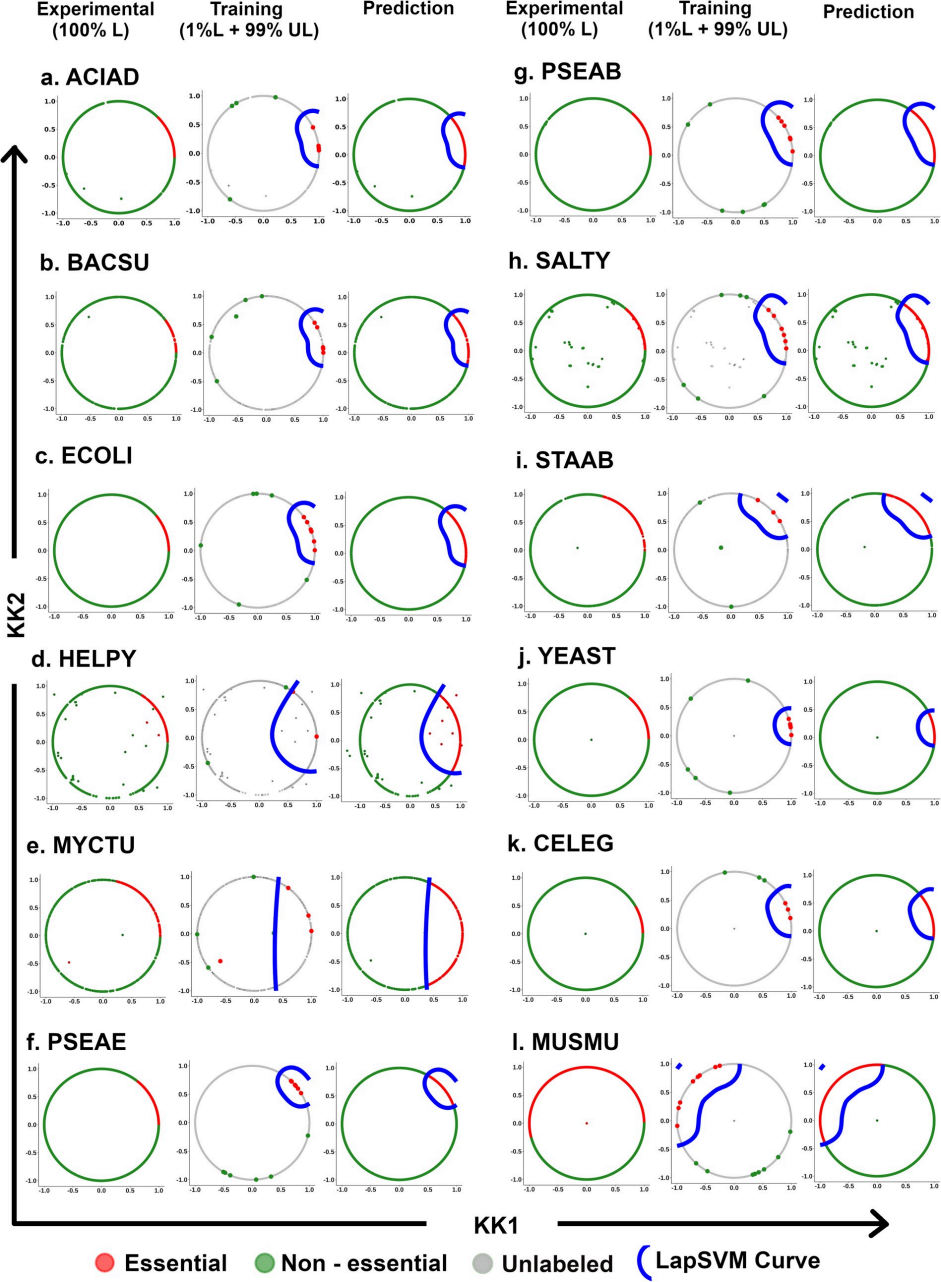
Visualization of the outcome of the proposed strategy. Essential, non-essential, and Unlabeled reaction gene pairs are colored accordingly Red, Green, and Gray. The learning curve for the best-trained model by LapSVM is colored with blue. The left circle represents the original data set with labeled data points. The middle circle shows the training data set with the learning curve, and the Right circle represents the prediction labeled with the learning curve.

To establish the predictive performance of the proposed strategy on the whole training data set, 1% labeled data were selected randomly, and the remaining 99% data points were considered unlabeled for the twelve organisms, where the information of gene essentiality in genome-scale was available from the experiments. Now, this whole data set was trained by the proposed strategy. The best model was selected based on the highest score (SSMSS). The same data set is used for prediction from the best-trained model. The outcome of the proposed strategy can be visualized as three circles (Fig 4). The first circle represents the circular projection of the whole data set in 2-D after applying the Kamada Kawai dimension reduction technique with gene essentiality information from the experiment. The second circle shows the training data set with 1% labeled & 99% Unlabeled data and learning curve of the Laplacian model. The third circle shows the predicted gene essentiality label from the best-trained model. From Fig 4, it is observed that the proposed model also performed well (as similar circular patterns from experiment and predicted) on the whole training data set.

The predictive performance on both the data sets (80% and the Whole data set) has been compared by six supervised performance metrics (i.e., TPR, FPR, F-measure, MCC, auROC, and accuracy) based on actual and predicted labels from the proposed strategy. Here it has been observed that the average predictive performance of the 100 trained model with 80% data set is similar to the performance on the whole data set (S4 Fig).

### 3.8. Categorization of reaction-gene pairs

**Fig 5:**
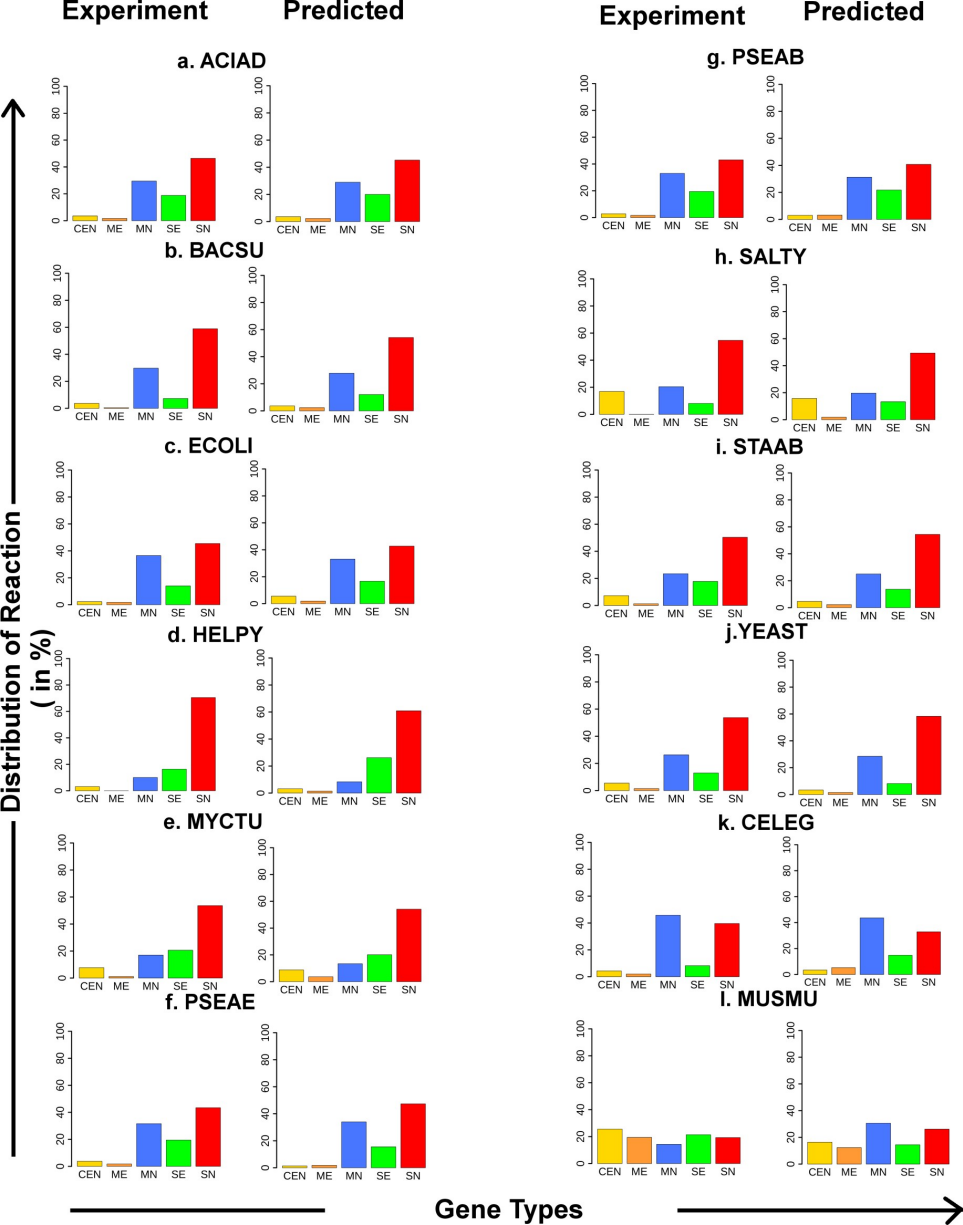
Comparison of the distributions of reaction. The reactions have been classified into five categories and the predicted distributions of reaction-gene pairs have been compared with the experimental data across all twelve organisms.

Categorization of the predicted essentiality information of reaction gene pairs into the five categories, viz. CEN, ME, MN, SE, and SN show that the distribution of reaction of the predicted results matches exactly with the distribution observed with the experimental data for each of the twelve organisms (Fig 5). Also, the Chi-square test was performed with a Null Hypothesis (H_0_) that the two distributions of reaction (experimental vs. predicted) are similar for all twelve organisms. Here, it has been observed that the *P*-values of the Chi-square test (*P*-values are indicated in S4 Table) are greater than 0.01 in all the 12 organisms. As *P-*values are large, it can be concluded that the experimental distributions of reaction are not significantly different from the predicted distributions. This pattern has been fairly consistent over all the organisms, where it is found that the highest fraction of reactions is regulated by single non-essential (red) or multiple non-essential genes (blue). On the other hand, fractions of reaction governed by a single essential gene are low due to a small number of minimally essential genes in all organisms. From this plot (Fig 5), it is also observed that the fractions of reactions governed by multiple essential genes are extremely low in each of the twelve organisms. These comprise the small set of reactions that are absolutely crucial for the survival of the organisms.

### 3.9. Case Study: *Leishmania donovani* and *Leishmania major*

**Fig 6:**
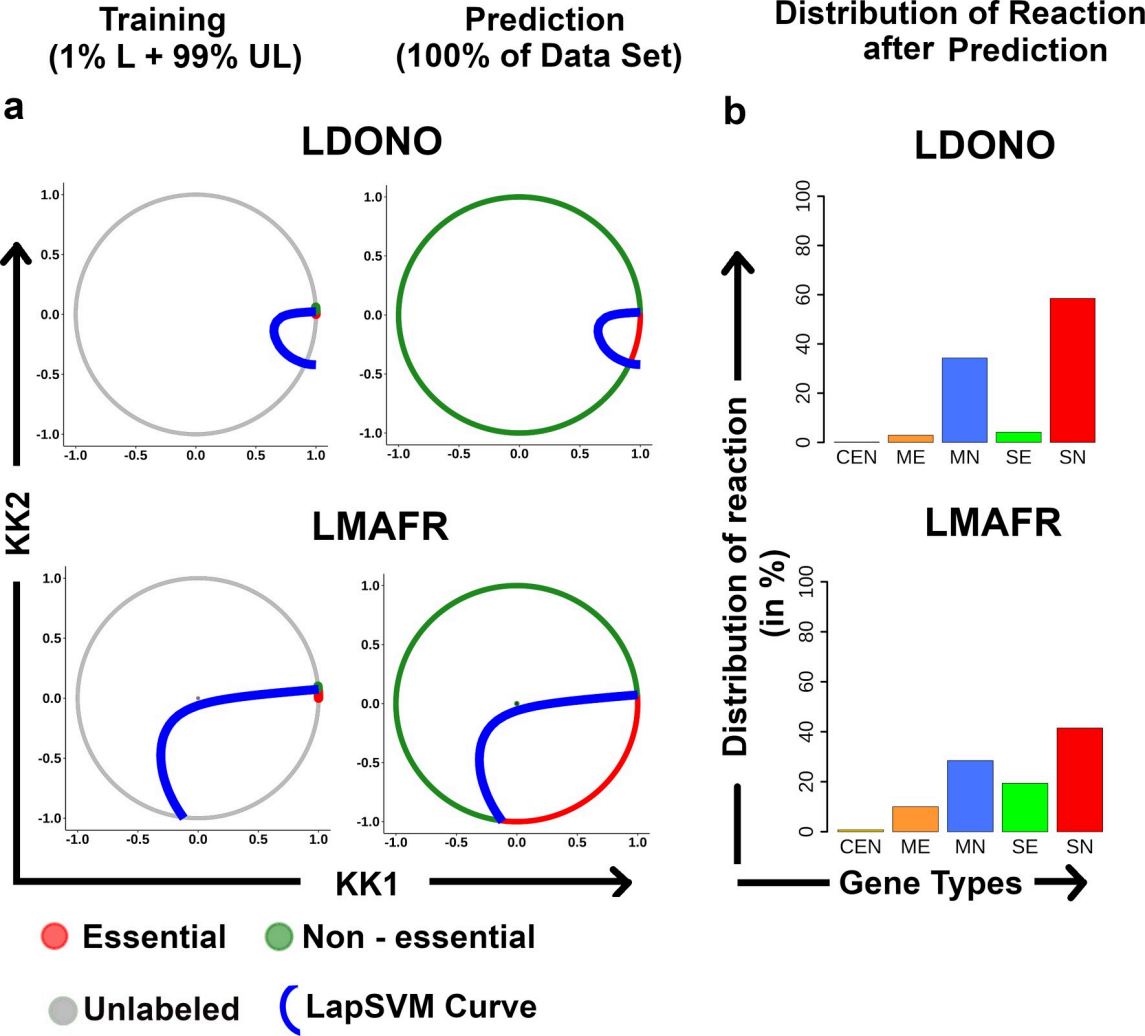
Gene essentiality prediction in *L*. *donovani* and *L*. *major*. **(a)** Kamada—Kawai dimension reduction on *Leishmania* datasets showed a circular pattern as observed for other organisms and the learning curve by LapSVM; (**b)** Distribution of reaction-gene pairs of *Leishmania* species into five categories.

The proposed strategy has been implemented for less explored organisms like *Leishmania donovani* (11 genes have genes essentiality information [102]) and *Leishmania major* (10 genes have genes essentiality information [102]) using the semi-supervised machine learning strategy. Here it is observed that the network centrality features and information-theoretic features, such as the Fourier cosine coefficient derived from the Kidera factor, have been selected by the feature selection algorithm in both the cases of *L*. *donovani* and *L*. *major*. Additionally, certain unique features were also selected for each of the two organisms (S1 Fig). When the Kamada-Kawai dimension reduction technique was applied on *Leishmania* data sets, a similar circular pattern was observed, like the other twelve organisms that helped the classifier in predicting gene essentiality (Fig 6A).

For the essential gene prediction, in the case of *Leishmania donovani*, 80 reaction-gene pairs were predicted as essential among 1129 reaction-gene pairs. For *Leishmania major*, 335 reaction-gene pairs were predicted as essential among 1188 reaction-gene pairs. The categorization of these reaction-gene pairs displayed a pattern similar to the distributions of reaction observed in the twelve model organisms (Fig 6B). Predicted gene essentiality information from the proposed pipeline is listed in (S5 and S6 Tables). The list of essential genes extracted from these reaction gene pairs consists of 44 essential genes of *L*.*donovani* and 194 of *L*. *major*.

These essential genes were associated with 53 and 219 Gene Ontology (Molecular Function) terms for *L*. *donovani* and *L*. *major*, respectively (S7 and S8 Tables). The Gene Ontology term that occurred most frequently with these essential genes were related to ATP binding in both the organisms. The pathway enrichment of these essential genes shows 11 significantly enriched KEGG pathways for *L*. *donovani* and 20 *L*. *major*. Although 8 KEGG pathways were found to be common among the two species, certain unique pathways specific to each species were also enriched for each of the two organisms (S9 and S10 Tables). Further experimental validation on these predicted results would confirm the role of these genes in these less-studied organisms.

## 4. Discussion

Essential gene prediction helps to unveil the complexities and survival strategies of many disease-causing organisms. The prediction of gene essentiality is a challenging task in machine learning due to the unavailability of sufficient experimentally labeled data and a proper metric for selection of the best model. Considering this limited gene essentiality information, the proposed pipeline has been able to predict gene essentiality at genome-scale using as small as a set of 1% labeled genes having gene essentiality information using both 80%-20% (training-blind testing) dataset as well as the whole dataset for training and testing (Figs 2 and 4). This proposed pipeline consists of three key steps. First, the unsupervised feature selection algorithm has been used to select the relevant feature set from 289 feature set consisting of different heterogeneous biological features such as sequence-based features, and topological features derived from metabolic reaction network, and flux-coupled sub-network which help to distinguish between essential and non-essential reaction gene combinations. Here, it is observed that for every organism, the features selection algorithm selected three phenotypic features that have shown high correlation with gene essentiality, *viz*., Reaction Network betweenness centrality (RN_betweenness), Reaction Network Page Rank centrality (RN_page_rank), and Flux Coupled Analysis Network Page Rank centrality (FCA_page_rank). Apart from these, novel features considered in this study, such as Information-theoretic features (Fourier sine coefficient and Fourier cosine coefficient derived from Kidera factor), were also correlated with gene essentiality prediction in most of the organisms. A distinguishing pattern between essential and non-essential genes for the selected features was captured by the feature selection algorithm, which helped the classifier to predict gene essentiality more accurately. Secondly, data set after feature selection was projected into a 2-D circular layout using the dimension reduction step Kamada-Kawai. This step is essential to project the high dimensional data into a 2-D plane, which helps the classifier LapSVM to perform significantly better for all the organisms (*P*<0.01) (S2 Table). The results show that this dimension reduction step is capable of improving the prediction accuracy even without feature selection (Fig 3, S3 Table). However, we have also retained the feature selection step in our pipeline to identify the important features that are contributing to gene essentiality classification. After applying Kamada-Kawai, a distinct structured pattern was observed, showing the essential reaction-gene combinations clustered together and the non-essential reaction-gene combination in another cluster, each residing on the arc of a 2-D circular layout for each of the twelve known organisms (Fig 4). This clustered pattern of reaction-gene pairs helped the semi-supervised classifier (Laplacian SVM) build a non-linear curve that dissects this circle into essential and non-essential classes with significantly higher accuracy. The novelty of the proposed strategy lies in the integration of the Kamada-Kawai algorithm with the semi-supervised LapSVM classifier that contributes to the high accuracy obtained using the pipeline. This is evident from S2 Table, where a significantly higher model performance of the Kamada-Kawai step was observed over the other widely used dimension reduction techniques. Further, it has been observed that the LapSVM classifier, when combined with the Kamada-Kawai step, contributes to the higher predictive performance of this pipeline as compared to the other supervised machine learning techniques when only 1% labeled data is available (S3 Fig).

Thereafter, the SSMSS score was used to select the best model. Here it was observed that the selected model based on this scoring technique had a corresponding high auROC value when compared with the experimentally known labels (S2 Fig). This indicated the reliability of the proposed SSMSS score, which, although show high variation for less number of labeled data, is useful as an alternative score when the calculation of supervised metrics is difficult for best model selection.

After the successful validation of this strategy on twelve organisms, the methodology was used to annotate gene essentiality in less-studied organisms like *Leishmania donovani* and *Leishmania major*, for which less or no organism-specific machine learning studies are available. Here, it was observed that 80 reaction-gene pairs were predicted to be essential in *Leishmania donovani*. These reactions involved 44 genes that were mostly associated with ATP binding [GO:0005524], oxidoreductase activity [GO:0016491], and AMP deaminase activity [GO:0003876] GO terms. Similarly, in the case of *Leishmania major*, 335 reaction-gene pairs were predicted as essential that involve 194 genes. Here it is observed that in addition to the ATP binding and metal-ion binding activities [GO:0005524], some genes that were predicted to be essential were also associated with amino acid transmembrane transporter activity [GO:0015171], magnesium ion binding [GO:0000287], and protein serine/threonine kinase activity [GO:0004674] GO terms that were not observed in the *L*. *donovani*. On the other hand, in the case of *L*. *donovani*, the genes involved in flavin adenine dinucleotide binding [GO:0050660] and AMP deaminase activity [GO:0003876] were predicted as essential, which is not observed in *L*. *major*.

The KEGG pathway enrichment study performed on the essential gene sets of the two organisms–*L*. *donovani* and *L*. *major* throw light on the pathways that are crucial for the survival of these micro-organisms and can be considered as probable therapeutic targets. Here, it is observed that apart from the pathways involved in Purine metabolism, Pyrimidine metabolism, Pyruvate metabolism, etc., that were common to both the organisms, a set of unique pathways were also enriched in each of *L*.*major* and *L*.*donovani*. While in the case of *L*. *major*, the pathways involved in Glycolysis/Gluconeogenesis, Glycine, serine and threonine metabolism, Citrate cycle (TCA cycle), Pyruvate metabolism, and Inositol phosphate metabolism were significantly enriched (*P*< 0.001), the essential genes of *L*. *donovani* show a higher enrichment for Sphingolipid metabolism and Steroid biosynthesis pathways. Further, the predicted essential reaction-gene combinations were categorized into five different groups (i.e., CEN, ME, MN, SE, and SN) that help to identify the individual reactions that are regulated by single or multiple essential genes. It may be mentioned here that a common pattern in these categories of distributions was observed across all the twelve organisms that corroborate well with the experimental observations (Fig 5). The Chi-Square Test performed to verify the difference in the experimental and predicted distributions showed no significant difference (S4 Table). A similar pattern was also predicted for *L*. *donovani* and *L*. *major* that further ascertains the validity of the predictions (Fig 6b). These results indicate the strength of the model in identifying true essential genes using a small amount labeled data, a selection of biologically relevant features to represent gene essentiality, and optimal parameters for curve formation to classify essential genes. The limitation of the proposed strategy is that, it requires the genome-scale reconstructed metabolic network, and at least 1% genes of this network should be annotated experimentally with gene essentiality information.

Using a graph-based semi-supervised machine learning scheme and combining different well-established methods in ML problems, a novel integrative approach has been proposed for essential gene prediction that shows universality in application to both prokaryotes and eukaryotes with limited labeled data. The run time of the pipeline is dependent on the size of the metabolic network (n), and the number of features (d) considered and can be represented as T(n,d) = *O(n*^*3*^*d*^*2*^*)*. In the case of *L*. *major* and *L donovani*, the total runtime was 41 minutes and 48 minutes, respectively, when simulated on a workstation of Intel(R) Xeon(R) CPU E5-2620 v4 @ 2.10GHz with 32GB RAM. This strategy will provide experimental biologists a well standardized and validated methodology to predict gene essentiality of less-studied organisms as well as will cater to the theoretical scientists with a novel approach for binary classification problems when limited labeled data is available. The essential genes predicted using the pipeline provide important leads for the identification of novel therapeutic targets for antibiotic and vaccine development against disease-causing parasites, such as *Leishmania sp*.

## Supporting information

S1 Fig Heatmap plot of selected features by the feature selection algorithm. Red cells indicate features selected by the feature selection algorithm in the corresponding organism. White cells show the feature that is not selected or is redundant.
(TIF)

S2 Fig Robustness evaluation of the proposed score (SSMSS). Scatter plots is demonstrating an association between auROC and SSMSS in each labeled category data sets in different model parameters conditions for twelve organisms. The X-axis represents the score (SSMSS), and Y-axis represents the corresponding auROC. To represent each category, ten different colors are used.
(TIF)

S3 Fig Comparison of the predictive performance of the proposed strategy with other supervised methods. Comparison of the performance of proposed strategy (PS) with supervised classifiers [i.e., Decision Tree (DT), Logistic regression (LR), Naive Bayes (NB), Random Forest (RF) and our own previously reported Supervised essential gene prediction pipeline] based on 1% labeled data on twelve organisms. The X-axis represents the different types of performance metrics for machine learning strategies, the Y-axis represents the value of performance metrics. Six different color codes were used to represent six different performance metrics.
(TIF)

S4 Fig Comparison of the predictive performance on both types of data sets (80% and whole data set). Average predictive performance of the best 100 models on 80% training data set and performance of whole training data set containing the Limited Labeled (L = 1%) and remaining Unlabeled (UL) data for six supervised metrics (i.e., TPR, FPR, F-measure, MCC, auROC, accuracy) and SSMSS for each labeled type. The X-axis represents the different performance metrics, the Y-axis represents the value of performance metrics.
(TIF)

S1 Table List of curated 289 features. List of curated 289 features for essential gene prediction.
(DOCX)

S2 Table Comparison of auROC of Kamada-Kawai (KK) dimension reduction technique with PCA, MDS, FR and ICA. The values reported in the table represent the *P-*values obtained using the one-tailed Mann-Whitney U Test.
(DOCX)

S3 Table Comparison of the effect of feature selection and Kamada-Kawai (KK) dimension reduction technique on the model performance (auROC). The values reported in the table represent the *P*-values obtained using the one-tailed Mann-Whitney U Test.
(DOCX)

S4 Table Comparison of percentage distribution of reaction into five categories from experiment vs predicted results. The values reported in the table represent the *P*-values obtained using the Chi-square test.
(DOCX)

S5 Table Gene essentiality information of reaction gene combinations in *Leishmania donovani* predicted using the proposed pipeline. (DOCX)

S6 Table. Gene essentiality information of reaction gene combinations in Leishmania major predicted using the proposed pipeline (DOCX)

S7 Table Gene Ontology (Molecular Function) terms of the predicted essential genes in *Leishmania donovani*. (DOCX)

S8 Table Gene Ontology (Molecular Function) terms of the predicted essential genes in *Leishmania major*. (DOCX)

S9 Table KEGG pathway enrichment of the predicted essential genes in *Leishmania donovani*. (DOCX)

S10 Table KEGG pathway enrichment of the predicted essential genes in *Leishmania major*. (DOCX)

S1 Text Source code of proposed machine learning strategy. This supplementary text contains source code for the proposed machine learning strategy, including codes for (a) Training data set preparation and integration of heterogeneous features; (b) Feature selection based on the space-filling concept; (c) Dimension reduction using forced directed graph layout; (d) Semi-supervised classifier LapSVM.
(DOCX)

## References

[1] Baba T, Ara T, Hasegawa M, Takai Y, Okumura Y, Baba M, et al Construction of Escherichia coli K-12 in-frame, single-gene knockout mutants: the Keio collection. Mol Syst Biol. 2006;2: 2006.0008. 10.1038/msb4100050 16738554 PMC1681482

[2] Cruz A, Coburn CM, Beverley SM. Double targeted gene replacement for creating null mutants. Proc Natl Acad Sci U S A. 1991;88: 7170–4. 10.1073/pnas.88.16.7170 1651496 PMC52255

[3] Gerdes SyS, Scholle MD, Campbell JW, Balazsi G, Ravasz E, Daugherty MD, et al Experimental determination and system level analysis of essential genes in Escherichia coli MG1655. J Bacteriol. 2003;185: 5673–5684. 10.1128/jb.185.19.5673-5684.2003 13129938 PMC193955

[4] Reznikoff WS, Winterberg KM. Transposon-based strategies for the identification of essential bacterial genes. Microb Gene Essentiality Protoc Bioinforma. 2008; 13–26. 10.1007/978-1-59745-321-9_2 18392958

[5] Agrawal N, Dasaradhi PVN, Mohmmed A, Malhotra P, Bhatnagar RK, Mukherjee SK. RNA interference: biology, mechanism, and applications. Microbiol Mol Biol Rev. 2003;67: 657–685. 10.1128/mmbr.67.4.657-685.2003 14665679 PMC309050

[6] Li X, Li W, Zeng M, Zheng R, Li M. Network-based methods for predicting essential genes or proteins: a survey. Brief Bioinform. 2019. 10.1093/bib/bbz017 30776072

[7] Zhang X, Acencio ML, Lemke N. Predicting essential genes and proteins based on machine learning and network topological features: A comprehensive review. Front Physiol. 2016;7: 1–11. 10.3389/fphys.2016.00001 27014079 PMC4781880

[8] Peng C, Lin Y, Luo H, Gao F. A comprehensive overview of online resources to identify and predict bacterial essential genes. Front Microbiol. 2017;8: 2331 10.3389/fmicb.2017.02331 29230204 PMC5711816

[9] Liu W, Fang L, Li M, Li S, Guo S, Luo R, et al Comparative genomics of Mycoplasma: analysis of conserved essential genes and diversity of the pan-genome. PLoS One. 2012;7: e35698 10.1371/journal.pone.0035698 22536428 PMC3335003

[10] Fagen JR, Leonard MT, McCullough CM, Edirisinghe JN, Henry CS, Davis MJ, et al Comparative genomics of cultured and uncultured strains suggests genes essential for free-living growth of Liberibacter. PLoS One. 2014;9: e84469 10.1371/journal.pone.0084469 24416233 PMC3885570

[11] Rout S, Warhurst DC, Suar M, Mahapatra RK. In silico comparative genomics analysis of Plasmodium falciparum for the identification of putative essential genes and therapeutic candidates. J Microbiol Methods. 2015;109: 1–8. 10.1016/j.mimet.2014.11.016 25486552

[12] Yang X, Li Y, Zang J, Li Y, Bie P, Lu Y, et al Analysis of pan-genome to identify the core genes and essential genes of Brucella spp. Mol Genet Genomics. 2016;291: 905–912. 10.1007/s00438-015-1154-z 26724943

[13] Bruccoleri RE, Dougherty TJ, Davison DB. Concordance analysis of microbial genomes. Nucleic Acids Res. 1998;26: 4482–4486. 10.1093/nar/26.19.4482 9742253 PMC147859

[14] Lu Y, Deng J, B Carson M, Lu H, J Lu L. Computational methods for the prediction of microbial essential genes. Curr Bioinform. 2014;9: 89–101.

[15] Joyce AR, Palsson BØ. Predicting gene essentiality using genome-scale in silico models Microbial Gene Essentiality: Protocols and Bioinformatics. Springer; 2008 pp. 433–457. 10.1007/978-1-59745-321-9_30 18392986

[16] Basler G. Computational prediction of essential metabolic genes using constraint-based approaches Gene Essentiality. Springer; 2015 pp. 183–204. 10.1007/978-1-4939-2398-4_12 25636620

[17] Dey A. Machine learning algorithms: a review. Int J Comput Sci Inf Technol. 2016;7: 1174–1179.

[18] Kotsiantis SB, Zaharakis I, Pintelas P. Supervised machine learning: A review of classification techniques. Emerg Artif Intell Appl Comput Eng. 2007;160: 3–24.

[19] Kennedy J, Eberhart R. Particle swarm optimization. Proceedings of ICNN’95-International Conference on Neural Networks. 1995 pp. 1942–1948.

[20] Bonabeau E, Dorigo M, Marco D de RDF, Theraulaz G, Théraulaz G, et al Swarm intelligence: from natural to artificial systems Oxford university press; 1999.

[21] Dorigo M, Birattari M, Stutzle T. Ant colony optimization. IEEE Comput Intell Mag. 2006;1: 28–39.

[22] Mirjalili S, Mirjalili SM, Lewis A. Grey wolf optimizer. Adv Eng Softw. 2014;69: 46–61.

[23] Mirjalili S. The ant lion optimizer. Adv Eng Softw. 2015;83: 80–98.

[24] Hasan MA, Lonardi S. DeeplyEssential: a deep neural network for predicting essential genes in microbes. BMC Bioinformatics. 2020;21: 1–19. 10.1186/s12859-020-03688-y 32998698 PMC7525945

[25] Zampieri G, Vijayakumar S, Yaneske E, Angione C. Machine and deep learning meet genome-scale metabolic modeling. PLoS Comput Biol. 2019;15: e1007084 10.1371/journal.pcbi.1007084 31295267 PMC6622478

[26] Deng J, Deng L, Su S, Zhang M, Lin X, Wei L, et al Investigating the predictability of essential genes across distantly related organisms using an integrative approach. Nucleic Acids Res. 2011;39: 795–807. 10.1093/nar/gkq784 20870748 PMC3035443

[27] Cheng J, Wu W, Zhang Y, Li X, Jiang X, Wei G, et al A new computational strategy for predicting essential genes. BMC Genomics. 2013;14: 910 10.1186/1471-2164-14-910 24359534 PMC3880044

[28] Hwang Y-C, Lin C-C, Chang J-Y, Mori H, Juan H-F, Huang H-C. Predicting essential genes based on network and sequence analysis. Mol Biosyst. 2009;5: 1672–1678. 10.1039/B900611G 19452048

[29] Plaimas K, Eils R, König R. Identifying essential genes in bacterial metabolic networks with machine learning methods. BMC Syst Biol. 2010;4: 1 10.1186/1752-0509-4-1 20438628 PMC2874528

[30] Plaimas K, Mallm J-P, Oswald M, Svara F, Sourjik V, Eils R, et al Machine learning based analyses on metabolic networks supports high-throughput knockout screens. BMC Syst Biol. 2008;2: 67 10.1186/1752-0509-2-67 18652654 PMC2526078

[31] Chen L, Zhang Y-H, Wang S, Zhang Y, Huang T, Cai Y-D. Prediction and analysis of essential genes using the enrichments of gene ontology and KEGG pathways. PLoS One. 2017;12: e0184129 10.1371/journal.pone.0184129 28873455 PMC5584762

[32] Qin C, Sun Y, Dong Y. A new computational strategy for identifying essential proteins based on network topological properties and biological information. PLoS One. 2017;12: e0182031 10.1371/journal.pone.0182031 28753682 PMC5533339

[33] Gustafson AM, Snitkin ES, Parker SCJ, DeLisi C, Kasif S. Towards the identification of essential genes using targeted genome sequencing and comparative analysis. BMC Genomics. 2006;7: 1 10.1186/1471-2164-7-1 17052348 PMC1624830

[34] Saha S, Heber S, et al In silico prediction of yeast deletion phenotypes. Genet Mol Res. 2006;5: 224–232. 16755513

[35] Jin S, Zeng X, Xia F, Huang W, Liu X. Application of deep learning methods in biological networks. Brief Bioinform. 2020 10.1093/bib/bbaa043 32363401

[36] Ning LW, Lin H, Ding H, Huang J, Rao N, Guo FB. Predicting bacterial essential genes using only sequence composition information. Genet Mol Res. 2014;13: 4564–4572. 10.4238/2014.June.17.8 25036505

[37] Nigatu D, Sobetzko P, Yousef M, Henkel W. Sequence-based information-theoretic features for gene essentiality prediction. BMC Bioinformatics. 2017;18: 473 10.1186/s12859-017-1884-5 29121868 PMC5679510

[38] Yu Y, Yang L, Liu Z, Zhu C. Gene essentiality prediction based on fractal features and machine learning. Mol Biosyst. 2017;13: 577–584. 10.1039/c6mb00806b 28145541

[39] Azhagesan K, Ravindran B, Raman K. Network-based features enable prediction of essential genes across diverse organisms. PLoS One. 2018;13: e0208722 10.1371/journal.pone.0208722 30543651 PMC6292609

[40] Nandi S, Subramanian A, Sarkar RR. An integrative machine learning strategy for improved prediction of essential genes in Escherichia coli metabolism using flux-coupled features. Mol Biosyst. 2017;13: 1584–1596. 10.1039/c7mb00234c 28671706

[41] Raman K, Damaraju N, Joshi GK. The organisational structure of protein networks: revisiting the centrality—lethality hypothesis. Syst Synth Biol. 2014;8: 73–81. 10.1007/s11693-013-9123-5 24592293 PMC3933631

[42] Guyon I, Elisseeff A. An introduction to variable and feature selection. J Mach Learn Res. 2003;3: 1157–1182.

[43] Platt JC. Fast training of support vector machines using sequential minimal optimization. Adv kernel methods. 1999; 185–208.

[44] Belkin M, Niyogi P, Sindhwani V. Manifold regularization: A geometric framework for learning from labeled and unlabeled examples. J Mach Learn Res. 2006;7: 2399–2434.

[45] Subramanian A, Sarkar RR. Perspectives on Leishmania Species and Stage-specific Adaptive Mechanisms. Trends Parasitol. 2018;34: 1068–1081. 10.1016/j.pt.2018.09.004 30318316

[46] Wei W, Ning L-W, Ye Y-N, Guo F-B. Geptop: a gene essentiality prediction tool for sequenced bacterial genomes based on orthology and phylogeny. PLoS One. 2013;8: e72343 10.1371/journal.pone.0072343 23977285 PMC3744497

[47] Matthews BW. Comparison of the predicted and observed secondary structure of T4 phage lysozyme. Biochim Biophys Acta (BBA)-Protein Struct. 1975;405: 442–451. 10.1016/0005-2795(75)90109-9 1180967

[48] Chen W-H, Minguez P, Lercher MJ, Bork P. OGEE: an online gene essentiality database. Nucleic Acids Res. 2011;40: D901—D906. 10.1093/nar/gkr986 22075992 PMC3245054

[49] Durot M, Le Fèvre F, de Berardinis V, Kreimeyer A, Vallenet D, Combe C, et al Iterative reconstruction of a global metabolic model of Acinetobacter baylyi ADP1 using high-throughput growth phenotype and gene essentiality data. BMC Syst Biol. 2008;2: 85 10.1186/1752-0509-2-85 18840283 PMC2606687

[50] Oh Y-K, Palsson BO, Park SM, Schilling CH, Mahadevan R. Genome-scale reconstruction of metabolic network in Bacillus subtilis based on high-throughput phenotyping and gene essentiality data. J Biol Chem. 2007;282: 28791–28799. 10.1074/jbc.M703759200 17573341

[51] Orth JD, Conrad TM, Na J, Lerman JA, Nam H, Feist AM, et al A comprehensive genome-scale reconstruction of Escherichia coli metabolism—2011. Mol Syst Biol. 2011;7: 535 10.1038/msb.2011.65 21988831 PMC3261703

[52] Thiele I, Vo TD, Price ND, Palsson BØ. Expanded metabolic reconstruction of Helicobacter pylori (iIT341 GSM/GPR): an in silico genome-scale characterization of single-and double-deletion mutants. J Bacteriol. 2005;187: 5818–5830. 10.1128/JB.187.16.5818-5830.2005 16077130 PMC1196094

[53] Jamshidi N, Palsson BØ. Investigating the metabolic capabilities of Mycobacterium tuberculosis H37Rv using the in silico strain iNJ 661 and proposing alternative drug targets. BMC Syst Biol. 2007;1: 26 10.1186/1752-0509-1-26 17555602 PMC1925256

[54] Bartell JA, Blazier AS, Yen P, Thøgersen JC, Jelsbak L, Goldberg JB, et al Reconstruction of the metabolic network of Pseudomonas aeruginosa to interrogate virulence factor synthesis. Nat Commun. 2017;8: 14631 10.1038/ncomms14631 28266498 PMC5344303

[55] Thiele I, Hyduke DR, Steeb B, Fankam G, Allen DK, Bazzani S, et al A community effort towards a knowledge-base and mathematical model of the human pathogen Salmonella Typhimurium LT2. BMC Syst Biol. 2011;5: 8 10.1186/1752-0509-5-8 21244678 PMC3032673

[56] Li C, Donizelli M, Rodriguez N, Dharuri H, Endler L, Chelliah V, et al BioModels Database: An enhanced, curated and annotated resource for published quantitative kinetic models. BMC Syst Biol. 2010;4: 92 10.1186/1752-0509-4-92 20587024 PMC2909940

[57] Monica LM, Palsson B, Herrgård MJ. Connecting extracellular metabolomic measurements to intracellular flux states in yeast. BMC Syst Biol. 2009;3: 37 10.1186/1752-0509-3-37 19321003 PMC2679711

[58] Yilmaz LS, Walhout AJM. A Caenorhabditis elegans genome-scale metabolic network model. Cell Syst. 2016;2: 297–311. 10.1016/j.cels.2016.04.012 27211857 PMC5387690

[59] Sigurdsson MI, Jamshidi N, Steingrimsson E, Thiele I, Palsson BØ. A detailed genome-wide reconstruction of mouse metabolism based on human Recon 1. BMC Syst Biol. 2010;4: 140 10.1186/1752-0509-4-140 20959003 PMC2978158

[60] Sharma M, Shaikh N, Yadav S, Singh S, Garg P. A systematic reconstruction and constraint-based analysis of Leishmania donovani metabolic network: identification of potential antileishmanial drug targets. Mol Biosyst. 2017;13: 955–969. 10.1039/c6mb00823b 28367572

[61] Chavali AK, Whittemore JD, Eddy JA, Williams KT, Papin JA. Systems analysis of metabolism in the pathogenic trypanosomatid Leishmania major. Mol Syst Biol. 2008;4 10.1038/msb.2008.15 18364711 PMC2290936

[62] O’Leary NA, Wright MW, Brister JR, Ciufo S, Haddad D, McVeigh R, et al Reference sequence (RefSeq) database at NCBI: current status, taxonomic expansion, and functional annotation. Nucleic Acids Res. 2015;44: D733—D745. 10.1093/nar/gkv1189 26553804 PMC4702849

[63] Subramanian A, Sarkar RR. Network structure and enzymatic evolution in Leishmania metabolism: a computational study. BIOMAT 2015: International Symposium on Mathematical and Computational Biology. 2016 pp. 1–20.

[64] del Rio G, Koschützki D, Coello G. How to identify essential genes from molecular networks? BMC Syst Biol. 2009;3: 1 10.1186/1752-0509-3-1 19822021 PMC2765966

[65] Burgard AP, Nikolaev E V, Schilling CH, Maranas CD. Flux coupling analysis of genome-scale metabolic network reconstructions. Genome Res. 2004;14: 301–312. 10.1101/gr.1926504 14718379 PMC327106

[66] Larhlimi A, David L, Selbig J, Bockmayr A. F2C2: a fast tool for the computation of flux coupling in genome-scale metabolic networks. BMC Bioinformatics. 2012;13: 57 10.1186/1471-2105-13-57 22524245 PMC3515416

[67] Barabási A-L, et al Network science. Cambridge university press; 2016 10.1017/nws.2016.2

[68] Liu X, Hong Z, Liu J, Lin Y, Rodr’iguez-Patón A, Zou Q, et al Computational methods for identifying the critical nodes in biological networks. Brief Bioinform. 2019. 10.1093/bib/bbz011 30753282

[69] Wang J, Li M, Wang H, Pan Y. Identification of essential proteins based on edge clustering coefficient. IEEE/ACM Trans Comput Biol Bioinforma. 2011;9: 1070–1080. 10.1109/TCBB.2011.147 22084147

[70] Csardi G, Nepusz T, et al The igraph software package for complex network research. InterJournal, Complex Syst. 2006;1695: 1–9.

[71] Mann S, Chen Y-PP. Bacterial genomic G+ C composition-eliciting environmental adaptation. Genomics. 2010;95: 7–15. 10.1016/j.ygeno.2009.09.002 19747541

[72] dos Reis M, Wernisch L, Savva R. Unexpected correlations between gene expression and codon usage bias from microarray data for the whole Escherichia coli K-12 genome. Nucleic Acids Res. 2003;31: 6976–6985. 10.1093/nar/gkg897 14627830 PMC290265

[73] Sharp PM, Li W-H. The codon adaptation index-a measure of directional synonymous codon usage bias, and its potential applications. Nucleic Acids Res. 1987;15: 1281–1295. 10.1093/nar/15.3.1281 3547335 PMC340524

[74] Subramanian A, Sarkar RR. Comparison of codon usage bias across Leishmania and Trypanosomatids to understand mRNA secondary structure, relative protein abundance and pathway functions. Genomics. 2015;106: 232–241. 10.1016/j.ygeno.2015.05.009 26043961

[75] Wright F. The ‘effective number of codons’ used in a gene. Gene. 1990;87: 23–29. 10.1016/0378-1119(90)90491-9 2110097

[76] Rice P, Longden I, Bleasby A. EMBOSS: the European molecular biology open software suite. Trends Genet. 2000;16: 276–277. 10.1016/s0168-9525(00)02024-2 10827456

[77] Bauer M, Schuster SM, Sayood K. The average mutual information profile as a genomic signature. BMC Bioinformatics. 2008;9: 48 10.1186/1471-2105-9-48 18218139 PMC2335307

[78] Jordan IK, Rogozin IB, Wolf YI, Koonin EV. Essential genes are more evolutionarily conserved than are nonessential genes in bacteria. Genome Res. 2002;12: 962–968. 10.1101/gr.87702 12045149 PMC1383730

[79] Scheraga HA, Rackovsky S. Global informatics and physical property selection in protein sequences. Proc Natl Acad Sci. 2016;113: 1808–1810. 10.1073/pnas.1525745113 26831093 PMC4763726

[80] Kidera A, Konishi Y, Oka M, Ooi T, Scheraga HA. Statistical analysis of the physical properties of the 20 naturally occurring amino acids. J Protein Chem. 1985;4: 23–55.

[81] Laib M, Kanevski M. A Novel Filter Algorithm for Unsupervised Feature Selection Based on a Space Filling Measure. ESANN 2018 proceedings, Eur Symp Artif Neural Networks, Comput Intell Mach Learn Bruges. 2018.

[82] Ang JC, Mirzal A, Haron H, Hamed HNA. Supervised, unsupervised, and semi-supervised feature selection: a review on gene selection. IEEE/ACM Trans Comput Biol Bioinforma. 2015;13: 971–989. 10.1109/TCBB.2015.2478454 26390495

[83] Mitra P, Murthy CA, Pal SK. Unsupervised feature selection using feature similarity. IEEE Trans Pattern Anal Mach Intell. 2002;24: 301–312.

[84] Kamada T, Kawai S, et al An algorithm for drawing general undirected graphs. Inf Process Lett. 1989;31: 7–15.

[85] Kraemer G, Reichstein M, Mahecha MD. dimRed and coRanking—unifying dimensionality reduction in R. R J. 2018;10: 342–358.

[86] Pearson K. LIII. On lines and planes of closest fit to systems of points in space. London, Edinburgh, Dublin Philos Mag J Sci. 1901;2: 559–572.

[87] Torgerson WS. Multidimensional scaling: I. Theory and method. Psychometrika. 1952;17: 401–419.

[88] Fruchterman TMJ, Reingold EM. Graph drawing by force-directed placement. Softw Pract Exp. 1991;21: 1129–1164.

[89] Hyvarinen A. Fast and robust fixed-point algorithms for independent component analysis. IEEE Trans Neural Networks. 1999;10: 626–634. 10.1109/72.761722 18252563

[90] Chapelle O, Scholkopf B, Zien A. Semi-supervised learning (chapelle, o. et al., eds.; 2006)[book reviews]. IEEE Trans Neural Networks. 2009;20: 542.

[91] Krijthe JH. RSSL: Semi-supervised Learning in R. International Workshop on Reproducible Research in Pattern Recognition. 2016 pp. 104–115.

[92] Rousseeuw PJ. Silhouettes: a graphical aid to the interpretation and validation of cluster analysis. J Comput Appl Math. 1987;20: 53–65.

[93] Ashburner M, Ball CA, Blake JA, Botstein D, Butler H, Cherry JM, et al Gene ontology: tool for the unification of biology. Nat Genet. 2000;25: 25 10.1038/75556 10802651 PMC3037419

[94] Consortium GO. The gene ontology resource: 20 years and still GOing strong. Nucleic Acids Res. 2018;47: D330—D338. 10.1093/nar/gky1055 PMC6323945 30395331

[95] Consortium U. UniProt: a worldwide hub of protein knowledge. Nucleic Acids Res. 2018;47: D506—D515. 10.1093/nar/gky1049 PMC6323992 30395287

[96] Huang DW, Sherman BT, Lempicki RA. Systematic and integrative analysis of large gene lists using DAVID bioinformatics resources. Nat Protoc. 2009;4: 44 10.1038/nprot.2008.211 19131956

[97] Kanehisa M, Sato Y, Furumichi M, Morishima K, Tanabe M. New approach for understanding genome variations in KEGG. Nucleic Acids Res. 2018;47: D590—D595. 10.1093/nar/gky962 PMC6324070 30321428

[98] Breiman L. Random forests. Mach Learn. 2001;45: 5–32.

[99] Friedman N, Geiger D, Goldszmidt M. Bayesian network classifiers. Mach Learn. 1997;29: 131–163.

[100] Hosmer DW Jr, Lemeshow S, Sturdivant RX. Applied logistic regression. John Wiley & Sons; 2013.

[101] Quinlan JR, et al Bagging, boosting, and C4. 5. AAAI/IAAI, Vol 1 1996 pp. 725–730.

[102] Jones NG, Catta-Preta CMC, Lima APCA, Mottram JC. Genetically validated drug targets in Leishmania: current knowledge and future prospects. ACS Infect Dis. 2018;4: 467–477. 10.1021/acsinfecdis.7b00244 29384366 PMC5902788

