# Supplementary figures and images for: Essential gene prediction using limited gene essentiality information–An integrative semi-supervised machine learning strategy
Source: PLoS One. 2020 Nov 30;15(11):e0242943. doi: 10.1371/journal.pone.0242943 (PMC7703937; doi:10.1371/journal.pone.0242943)

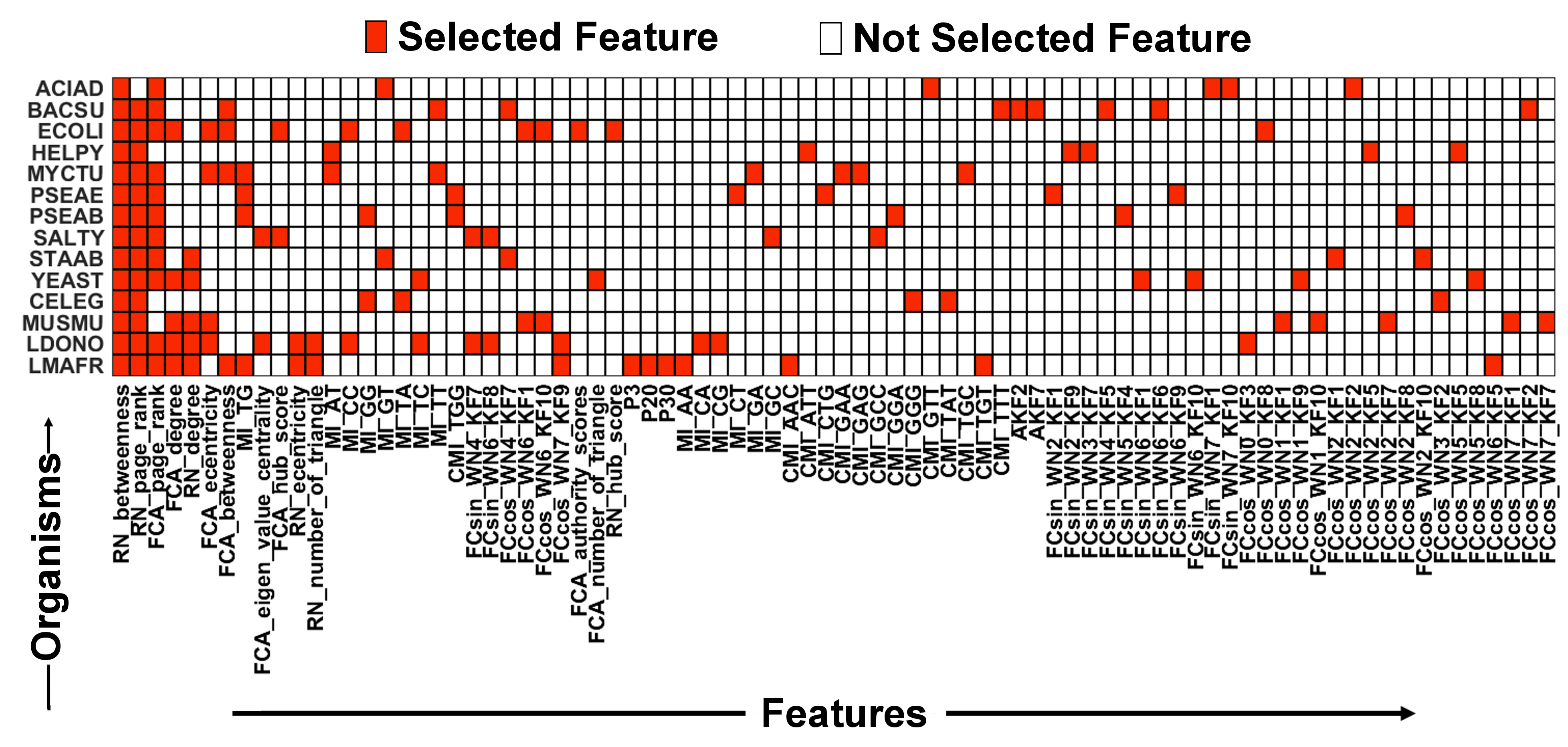

Supplement: S1 Fig — Red cells indicate features selected by the feature selection algorithm in the corresponding organism. White cells show the feature that is not selected or is redundant. (TIF) [file pone.0242943.s001.tif]

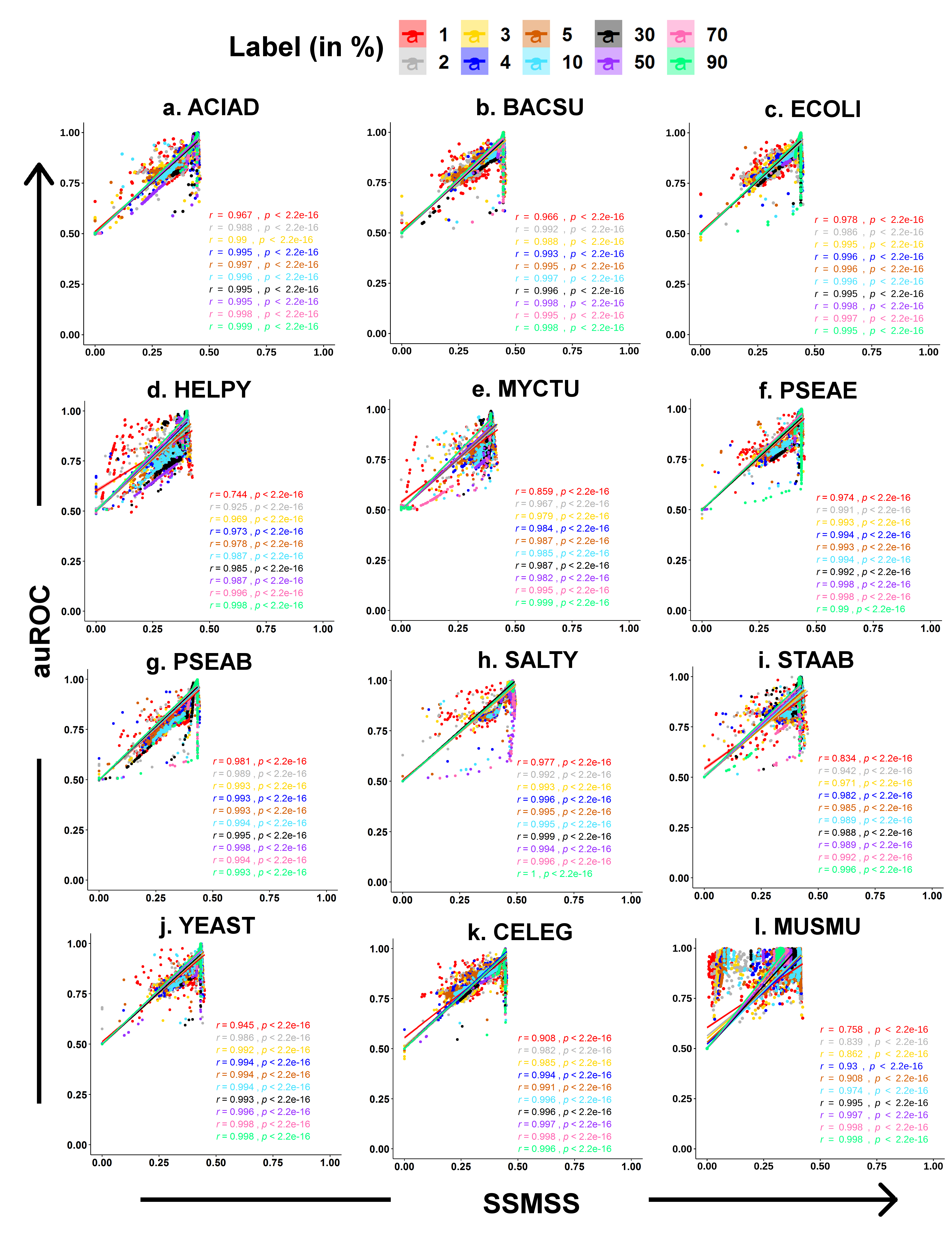

Supplement: S2 Fig — Scatter plots is demonstrating an association between auROC and SSMSS in each labeled category data sets in different model parameters conditions for twelve organisms. The X-axis represents the score (SSMSS), and Y-axis represents the corresponding auROC. To represent each category, ten different colors are used. (TIF) [file pone.0242943.s002.tif]

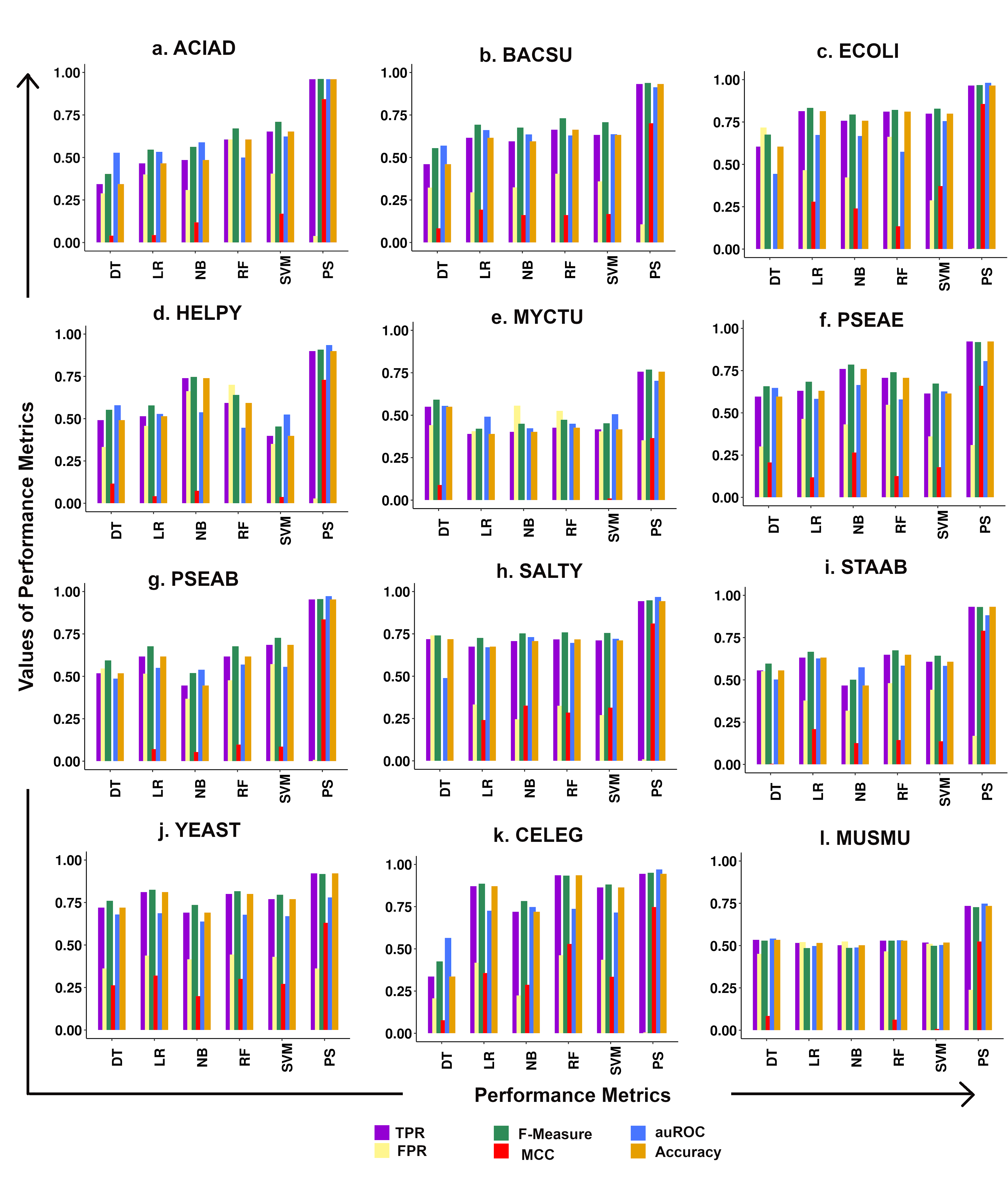

Supplement: S3 Fig — Comparison of the performance of proposed strategy (PS) with supervised classifiers [i.e., Decision Tree (DT), Logistic regression (LR), Naive Bayes (NB), Random Forest (RF) and our own previously reported Supervised essential gene prediction pipeline] based on 1% labeled data on twelve organisms. The X-axis represents the different types of performance metrics for machine learning strategies, the Y-axis represents the value of performance metrics. Six different color codes were used to represent six different performance metrics. (TIF) [file pone.0242943.s003.tif]

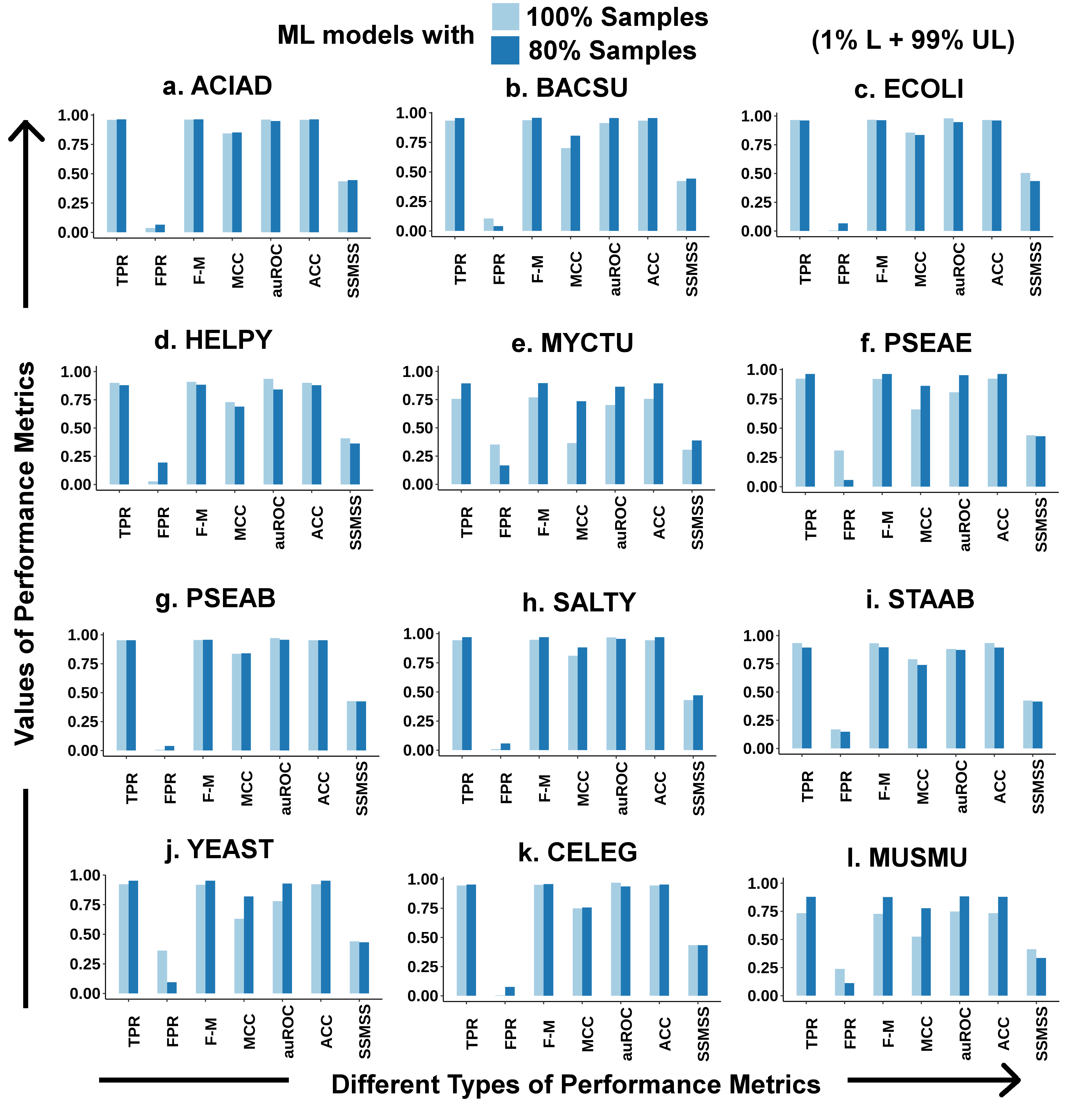

Supplement: S4 Fig — Average predictive performance of the best 100 models on 80% training data set and performance of whole training data set containing the Limited Labeled (L = 1%) and remaining Unlabeled (UL) data for six supervised metrics (i.e., TPR, FPR, F-measure, MCC, auROC, accuracy) and SSMSS for each labeled type. The X-axis represents the different performance metrics, the Y-axis represents the value of performance metrics. (TIF) [file pone.0242943.s004.tif]
