# Supplementary material for: Essential gene prediction using limited gene essentiality information–An integrative semi-supervised machine learning strategy
Source: PLoS One. 2020 Nov 30;15(11):e0242943. doi: 10.1371/journal.pone.0242943 (PMC7703937; doi:10.1371/journal.pone.0242943)
Supplement: S1 Table — List of curated 289 features for essential gene prediction. (DOCX) [file pone.0242943.s005.docx]

**Table S1. List of curated 289 features**

| **Feature Types** | **Features name** | **Abbreviation of features name** | **# of features** |
| --- | --- | --- | --- |
| **Topological analysis of reaction and flux-coupled sub-network** | | | |
| Reaction Network | Degree Centrality | RN_degree | 8 |
|  | Eigenvalue Centrality | RN_eigen_value_centrality |  |
|  | Eccentricity | RN_ecentricity |  |
|  | Hub Score | RN_hub_score |  |
|  | Authority Score | RN_authority_scores |  |
|  | Page Rank | RN_page_rank |  |
|  | Betweenness Centrality | RN_betweenness |  |
|  | Number of triangle | RN_number_of_triangle |  |
| Flux Coupled Network | Degree Centrality | FCA_degree | 8 |
|  | Eigenvalue Centrality | FCA_eigen_value_centrality |  |
|  | Eccentricity | FCA_ecentricity |  |
|  | Hub Score | FCA_hub_score |  |
|  | Authority Score | FCA_authority_scores |  |
|  | Page Rank | FCA_page_rank |  |
|  | Betweenness Centrality | FCA_betweenness |  |
|  | Number of triangle | FCA_number_of_triangle |  |
| **Features derived from the coding nucleotide sequence** | | | |
| Derived features | Nucleotide content | A3, T3, G3, C3 | 4 |
|  | Effective Number of Codons | ENC | 1 |
|  | Codon Adaptation Index | CAI | 1 |
| Information-theoretic features | Mutual Information (MI) | MI_(X,Y)  where $X,Y\in\{A,T,G,C\}$ | 16 |
|  | Conditional Mutual Information (CMI) | CMI_(X,Y,Z)  where $X,Y,Z\in\{A,T,G,C\}$ | 64 |
| **Features derived from protein sequence** | | | |
| Derived features | Frequencies of the twenty amino acids | Alanine, Cysteine, AsparticAcid, GlutamicAcid, Phenylalanine, Glycine, Histidine, Isoleucine, Lysine, Leucine, Asparagine, Proline, Glutamate, Arginine, Serine, Threonine, Valine, Tryptophan, Tyrosine, Methionine | 20 |
|  | Protein length | PL | 1 |
|  | Paralogy based features (Paralogy score) | P3, P5, P7, P10, P20, P30 | 6 |
| Information-theoretic features | Fourier sine coefficient | $FC\sin WN_{k}\_KF_{n}$  where $1\leq k\leq7$ and $1\leq n\leq10$ | 70 |
|  | Fourier cosine coefficient | $FC\cos WN_{k}\_KF_{n}$  where $0\leq k\leq7$ and $1\leq n\leq10$ | 80 |
|  | Average Kidera Factor | $AKF_{i}$  where i= 1 to 10 | 10 |
