# Supplementary material for: Essential gene prediction using limited gene essentiality information–An integrative semi-supervised machine learning strategy
Source: PLoS One. 2020 Nov 30;15(11):e0242943. doi: 10.1371/journal.pone.0242943 (PMC7703937; doi:10.1371/journal.pone.0242943)
Supplement: S2 Table — The values reported in the table represent the P-values obtained using the one-tailed Mann-Whitney U Test. (DOCX) [file pone.0242943.s006.docx]

**Table S2. Comparison of auROC of Kamada-Kawai (KK) dimension Reduction technique with PCA, MDS, FR and ICA.** The values reported in the table represent the *P*-values obtained using the one-tailed Mann-Whitney U Test.

| **Organisms** | **Scenario 6 (S6): [WOFS+DR(KK)]** | **Scenario 2 (S2):**  **[WOFS+DR(PCA)]** | **Scenario 3 (S3):**  **[WOFS+DR(MDS)]** | **Scenario 4 (S4):**  **[WOFS+DR(FR)]** | **Scenario5 (S5):**  **[WOFS+DR(ICA)]** |
| --- | --- | --- | --- | --- | --- |
| **ACIAD** |  | 2.45E-30 | 5.31E-30 | 8.77E-35 | 4.34E-30 |
| **BACSU** |  | 5.99E-29 | 2.21E-28 | 1.25E-29 | 1.87E-29 |
| **CELEG** |  | 2.09E-30 | 2.65E-30 | 1.91E-30 | 1.47E-30 |
| **ECOLI** |  | 2.36E-22 | 2.09E-22 | 4.42E-20 | 7.28E-23 |
| **HELPY** |  | 6.81E-31 | 6.81E-31 | 5.02E-36 | 2.55E-31 |
| **MUSMU** |  | 2.56E-10 | 5.55E-11 | 7.37E-10 | 2.93E-11 |
| **MYCTU** |  | 3.56E-26 | 3.27E-26 | 3.73E-26 | 1.25E-25 |
| **PSEAB** |  | 1.16E-24 | 2.63E-28 | 9.94E-24 | 1.13E-27 |
| **PSEAE** |  | 4.53E-28 | 4.53E-28 | 4.63E-30 | 1.67E-29 |
| **SALTY** |  | 9.56E-27 | 7.08E-27 | 6.06E-25 | 1.14E-26 |
| **STAAB** |  | 7.20E-31 | 2.08E-31 | 5.98E-34 | 2.22E-31 |
| **YEAST** |  | 1.23E-30 | 1.22E-30 | 3.88E-26 | 2.22E-31 |

**Note:** Null Hypothesis (H_0_) is that the auROC of Scenario 6 [S6: WOFS + DR (KK)] is not different from the auROC of Scenarios 2 to 5 for all twelve organisms. Alternative Hypothesis (H_1_) is that the auROC of Scenario 6 [S6: WOFS + DR (KK)] is greater than the auROC of Scenarios 2 to 5 for all twelve organisms.
