# Supplementary material for: Essential gene prediction using limited gene essentiality information–An integrative semi-supervised machine learning strategy
Source: PLoS One. 2020 Nov 30;15(11):e0242943. doi: 10.1371/journal.pone.0242943 (PMC7703937; doi:10.1371/journal.pone.0242943)
Supplement: S3 Table — The values reported in the table represent the P-values obtained using the one-tailed Mann-Whitney U Test. (DOCX) [file pone.0242943.s007.docx]

**Table S3. Comparison of the effect of feature selection and Kamada-Kawai (KK) dimension Reduction technique on the model performance (auROC).** The values reported in the table represent the *P*-values obtained using the one-tailed Mann-Whitney U Test.

| **Organisms** | **Scenario 1 (S1):**  **[WOFS+WODR]** | **Scenario 6 (S6):**  **[WOFS+DR(KK)]** | **Scenario 7 (S7):**  **[WFS (UFS)+DR(KK)]** |
| --- | --- | --- | --- |
| ACIAD |  | 5.42E-31 | 4.26E-32 |
| BACSU |  | 4.43E-30 | 1.95E-30 |
| CELEG |  | 1.08E-30 | 1.08E-30 |
| ECOLI |  | 5.82E-23 | 7.41E-30 |
| HELPY |  | 4.44E-32 | 2.43E-32 |
| MUSMU |  | 1.51E-12 | 3.59E-19 |
| MYCTU |  | 2.86E-25 | 4.93E-32 |
| PSEAB |  | 1.35E-26 | 1.71E-31 |
| PSEAE |  | 7.09E-30 | 6.58E-33 |
| SALTY |  | 5.85E-27 | 9.45E-33 |
| STAAB |  | 2.26E-33 | 8.95E-34 |
| YEAST |  | 2.15E-31 | 2.15E-31 |

**Note:** Null Hypothesis (H_0_) is that the auROC of Scenario 1 [S1: WOFS + WODR] is not different from the auROC of Scenarios 6 and 7 for all twelve organisms. Alternative Hypothesis (H_1_) is that the auROC of Scenario 1 [S1: WOFS + WODR] is less than the auROC of Scenarios 6 and 7 for all twelve organisms.
