# Supplementary material for: Essential gene prediction using limited gene essentiality information–An integrative semi-supervised machine learning strategy
Source: PLoS One. 2020 Nov 30;15(11):e0242943. doi: 10.1371/journal.pone.0242943 (PMC7703937; doi:10.1371/journal.pone.0242943)
Supplement: S4 Table — The values reported in the table represent the P-values obtained using the Chi-square test. (DOCX) [file pone.0242943.s008.docx]

**Table S4. Comparison of percentage distribution of reaction into five categories from experiment vs predicted results.** The values reported in the table represent the *P*-values obtained using the Chi-square test.

| **Orgaisms** | ***P*-value** |
| --- | --- |
| ACIAD | 0.996989 |
| BACSU | 0.599506 |
| ECOLI | 0.760862 |
| HELPY | 0.335664 |
| MYCTU | 0.764309 |
| PSEAE | 0.760246 |
| PSEAB | 0.944716 |
| SALTY | 0.504629 |
| STAAB | 0.808256 |
| YEAST | 0.768774 |
| CELEG | 0.391964 |
| MUSMU | 0.018437 |

**Note:** Null Hypothesis (H_0_) is that the two distributions of reaction (experimental vs. predicted) are not different for all twelve organisms. Alternative Hypothesis (H_1_) is that the two distributions of reaction (experimental vs. predicted) are different for all twelve organisms.
