# Supplementary material for: Essential gene prediction using limited gene essentiality information–An integrative semi-supervised machine learning strategy
Source: PLoS One. 2020 Nov 30;15(11):e0242943. doi: 10.1371/journal.pone.0242943 (PMC7703937; doi:10.1371/journal.pone.0242943)
Supplement: S5 Table — (DOCX) [file pone.0242943.s009.docx]

**S5 Table. Gene essentiality information of Reaction Gene combinations in *Leishmania donovani* predicted using our proposed pipeline**

| **Abbreviation** |
| --- |
| E, Essential gene |
| N, Non-essential gene |
| UD, Undetermined (Not Known) |

^#^Experimental Gene Essentiality Information ( Jones,N.G. et al. (2018) ACS Infect. Dis., 4, 467–477.)

| **GeneReaction** | **Experiment^#^** | **Predicted** |  | **GeneReaction** | **Experiment^#^** | **Predicted** |  | **GeneReaction** | **Experiment^#^** | **Predicted** |
| --- | --- | --- | --- | --- | --- | --- | --- | --- | --- | --- |
| 4COUCOAL\|LdBPK_303150 | E | E |  | DAGCPT\|LdBPK_281250 | UD | N |  | HINDOALDtm\|LdBPK_040960 | UD | N |
| DKMD2K\|LdBPK_160590 | E | E |  | DAGCPTm\|LdBPK_010490 | UD | N |  | HINDOALDtm\|LdBPK_340120 | UD | N |
| LACDtg\|LdBPK_341110 | E | E |  | DAGCPTm\|LdBPK_010510 | UD | N |  | HINDOALDtm\|LdBPK_340130 | UD | N |
| MLTHFtm\|LdBPK_040570 | E | E |  | DAGCPTm\|LdBPK_010520 | UD | N |  | HISt6\|LdBPK_060890 | UD | N |
| PMTCOAtm\|LdBPK_120105 | E | E |  | DAGCPTm\|LdBPK_010540 | UD | N |  | HISTRS\|LdBPK_343580 | UD | N |
| THRt6\|LdBPK_181500 | UD | E |  | DAGCPTm\|LdBPK_010550 | UD | N |  | HKAm\|LdBPK_180440 | UD | N |
| THRt6\|LdBPK_181510 | UD | E |  | DAGCPTm\|LdBPK_030220 | UD | N |  | HKAm\|LdBPK_191380 | UD | N |
| THYMDtm\|LdBPK_060910 | UD | E |  | DAGCPTm\|LdBPK_130300 | UD | N |  | HMGCOARx\|LdBPK_261530 | UD | N |
| THYMDtm\|LdBPK_070150 | UD | E |  | DAGCPTm\|LdBPK_281250 | UD | N |  | HMGCOARx\|LdBPK_292420 | UD | N |
| THYMDtm\|LdBPK_282700 | UD | E |  | DAGK\|LdBPK_010490 | UD | N |  | HMGCOARx\|LdBPK_350360 | UD | N |
| THYMDtm\|LdBPK_352780 | UD | E |  | DAGK\|LdBPK_010510 | UD | N |  | HMGCOAtg\|LdBPK_323110 | UD | N |
| TKT1g\|LdBPK_341170 | UD | E |  | DAGK\|LdBPK_010520 | UD | N |  | HMGCOAtg\|LdBPK_353910 | UD | N |
| TKT2g\|LdBPK_341170 | UD | E |  | DAGK\|LdBPK_010540 | UD | N |  | HMGS\|LdBPK_180580 | UD | N |
| TMDPP\|LdBPK_302920 | UD | E |  | DAGK\|LdBPK_010550 | UD | N |  | HMGS\|LdBPK_323830 | UD | N |
| TMDPP\|LdBPK_365410 | UD | E |  | DAGK\|LdBPK_030220 | UD | N |  | HMGS\|LdBPK_332740 | UD | N |
| TMDS\|LdBPK_120200 | UD | E |  | DAGK\|LdBPK_130300 | UD | N |  | HOCHL3m\|LdBPK_260200 | UD | N |
| TPIg\|LdBPK_362740 | UD | E |  | DAGK\|LdBPK_281250 | UD | N |  | HOCHL3m\|LdBPK_341900 | UD | N |
| TRNAGLNtm\|LdBPK_290920 | UD | E |  | DAGKm\|LdBPK_261530 | UD | N |  | HOCR3m\|LdBPK_292080 | UD | N |
| TRNAGLUtm\|LdBPK_230500 | UD | E |  | DAGKm\|LdBPK_332740 | UD | N |  | HPROa\|LdBPK_070930 | UD | N |
| TRNAGLUtm\|LdBPK_364510 | UD | E |  | DAGKm\|LdBPK_361200 | UD | N |  | HPROxm\|LdBPK_210300 | UD | N |
| TROPtm\|LdBPK_040440 | UD | E |  | DATPtn\|LdBPK_010310 | UD | N |  | HPROxm\|LdBPK_210310 | UD | N |
| TRPDOXtn\|LdBPK_323110 | UD | E |  | DCCOADm\|LdBPK_363750 | UD | N |  | HPROym\|LdBPK_283250 | UD | N |
| TRPDOXtn\|LdBPK_353910 | UD | E |  | DCTPtn\|LdBPK_331150 | UD | N |  | HSD\|LdBPK_321220 | UD | N |
| TRPDRDtn\|LdBPK_040270 | UD | E |  | DCTPtn\|LdBPK_362390 | UD | N |  | HSK\|LdBPK_140700 | UD | N |
| TRPDRDtn\|LdBPK_130870 | UD | E |  | DCYTD1\|LdBPK_240870 | UD | N |  | HTDR6m\|LdBPK_240310 | UD | N |
| TRPDRDtn\|LdBPK_322690 | UD | E |  | DCYTt\|LdBPK_366670 | UD | N |  | HXANt2\|LdBPK_354790 | UD | N |
| TRPDRDtn\|LdBPK_354860 | UD | E |  | DDMAT5m\|LdBPK_180690 | UD | N |  | HXANt2\|LdBPK_363990 | UD | N |
| TRPTRS\|LdBPK_311800 | UD | E |  | DDMAT5m\|LdBPK_180700 | UD | N |  | HXANt2\|LdBPK_364000 | UD | N |
| TRYPm\|LdBPK_241910 | UD | E |  | DDMAT5m5\|LdBPK_100310 | UD | N |  | HXANtg\|LdBPK_230580 | UD | N |
| TRYR\|LdBPK_241910 | UD | E |  | DDMAT5m5\|LdBPK_332680 | UD | N |  | HXANtg\|LdBPK_230880 | UD | N |
| TYRabc\|LdBPK_010470 | UD | E |  | DESAT224_4\|LdBPK_340630 | UD | N |  | HXPRTg\|LdBPK_354660 | UD | N |
| TYRTA\|LdBPK_180440 | UD | E |  | DGDPter\|LdBPK_350840 | UD | N |  | HYDCOAtm\|LdBPK_350840 | UD | N |
| TYRTA\|LdBPK_191380 | UD | E |  | DGDPter\|LdBPK_362490 | UD | N |  | HYDCOAtm\|LdBPK_362490 | UD | N |
| TYRTA2\|LdBPK_120580 | UD | E |  | DGK1er\|LdBPK_210300 | UD | N |  | ICDHym\|LdBPK_261590 | UD | N |
| TYRTA2\|LdBPK_350840 | UD | E |  | DGK1er\|LdBPK_210310 | UD | N |  | IDPtn\|LdBPK_331150 | UD | N |
| TYRTA3\|LdBPK_260680 | UD | E |  | dGTPte\|LdBPK_140330 | UD | N |  | IDPtn\|LdBPK_362390 | UD | N |
| UAGDP\|LdBPK_050180 | UD | E |  | dGTPte\|LdBPK_271500 | UD | N |  | ILETAc\|LdBPK_180020 | UD | N |
| UAGDP\|LdBPK_250020 | UD | E |  | dGTPte\|LdBPK_310350 | UD | N |  | IMPDg\|LdBPK_251160 | UD | N |
| UDPDPS\|LdBPK_366170 | UD | E |  | dGTPte\|LdBPK_310360 | UD | N |  | IMZACtm\|LdBPK_050180 | UD | N |
| UDPGALtg\|LdBPK_230580 | UD | E |  | dGTPte\|LdBPK_311850 | UD | N |  | IMZACtm\|LdBPK_250020 | UD | N |
| UDPGALtg\|LdBPK_230880 | UD | E |  | dGTPte\|LdBPK_311860 | UD | N |  | IND3ACtm\|LdBPK_270310 | UD | N |
| UDPGtg\|LdBPK_181580 | UD | E |  | dGTPte\|LdBPK_322800 | UD | N |  | INDPYRD\|LdBPK_322440 | UD | N |
| UDPtn\|LdBPK_252480 | UD | E |  | dGTPte\|LdBPK_331510 | UD | N |  | INSter\|LdBPK_070240 | UD | N |
| UDPtn\|LdBPK_361410 | UD | E |  | dGTPte\|LdBPK_355070 | UD | N |  | INSter\|LdBPK_310010 | UD | N |
| UDPx\|LdBPK_241880 | UD | E |  | dGTPte\|LdBPK_355320 | UD | N |  | INStm\|LdBPK_060910 | UD | N |
| UDPx\|LdBPK_242110 | UD | E |  | dGTPte\|LdBPK_360450 | UD | N |  | INStm\|LdBPK_070150 | UD | N |
| UDPx\|LdBPK_272390 | UD | E |  | dGTPte\|LdBPK_364700 | UD | N |  | INStm\|LdBPK_282700 | UD | N |
| UMPK\|LdBPK_271940 | UD | E |  | dGTPte\|LdBPK_367150 | UD | N |  | INStm\|LdBPK_352780 | UD | N |
| UMPK\|LdBPK_290290 | UD | E |  | DHAPAx\|LdBPK_261530 | UD | N |  | IPDDI\|LdBPK_270740 | UD | N |
| URAtn\|LdBPK_323110 | UD | E |  | DHAPAx\|LdBPK_332740 | UD | N |  | IPDDI\|LdBPK_363630 | UD | N |
| URAtn\|LdBPK_353910 | UD | E |  | DHAPAx\|LdBPK_361200 | UD | N |  | IPP5P\|LdBPK_352790 | UD | N |
| UREAt\|LdBPK_360280 | UD | E |  | DHAPtg\|LdBPK_060580 | UD | N |  | ITPtn\|LdBPK_330250 | UD | N |
| UREAtg\|LdBPK_170410 | UD | E |  | DHCRD2\|LdBPK_141450 | UD | N |  | KYNASE1\|LdBPK_261670 | UD | N |
| UREAtg\|LdBPK_322210 | UD | E |  | DHDASCBtm\|LdBPK_060910 | UD | N |  | KYNASE1\|LdBPK_261680 | UD | N |
| URItn\|LdBPK_210990 | UD | E |  | DHDASCBtm\|LdBPK_070150 | UD | N |  | KYNASE3\|LdBPK_311800 | UD | N |
| VALt6\|LdBPK_050980 | UD | E |  | DHDASCBtm\|LdBPK_282700 | UD | N |  | LDH_D\|LdBPK_030790 | UD | N |
| VALt6\|LdBPK_170320 | UD | E |  | DHDASCBtm\|LdBPK_352780 | UD | N |  | LDH_D\|LdBPK_260300 | UD | N |
| VALt6\|LdBPK_181460 | UD | E |  | DHFOR2a\|LdBPK_340630 | UD | N |  | LDH_L\|LdBPK_321660 | UD | N |
| VALt6\|LdBPK_270300 | UD | E |  | DHFRa\|LdBPK_230410 | UD | N |  | LEUt6\|LdBPK_282530 | UD | N |
| VALt6\|LdBPK_270590 | UD | E |  | DHNPTtn\|LdBPK_323110 | UD | N |  | LEUTAm\|LdBPK_180020 | UD | N |
| VALt6\|LdBPK_282170 | UD | E |  | DHNPTtn\|LdBPK_353910 | UD | N |  | LGLUtg\|LdBPK_323110 | UD | N |
| VALt6\|LdBPK_323370 | UD | E |  | DHORTS\|LdBPK_120200 | UD | N |  | LGLUtg\|LdBPK_353910 | UD | N |
| VALt6\|LdBPK_352010 | UD | E |  | DHPEG\|LdBPK_343530 | UD | N |  | LGTHL1m\|LdBPK_070870 | UD | N |
| VALt6\|LdBPK_365620 | UD | E |  | DHPEG\|LdBPK_350320 | UD | N |  | LGTHL1m\|LdBPK_360160 | UD | N |
| VALTAc\|LdBPK_161410 | UD | E |  | DITPtn\|LdBPK_323110 | UD | N |  | LINCOAe\|LdBPK_270430 | UD | N |
| VALTRS\|LdBPK_060350 | UD | E |  | DITPtn\|LdBPK_353910 | UD | N |  | LINCOAtm\|LdBPK_220013 | UD | N |
| VTm\|LdBPK_061330 | UD | E |  | DMATTx\|LdBPK_251160 | UD | N |  | LIPOAS2\|LdBPK_332430 | UD | N |
| XANt2\|LdBPK_331010 | UD | E |  | DNTPPAn\|LdBPK_340630 | UD | N |  | LIPOT\|LdBPK_171260 | UD | N |
| XANtg\|LdBPK_221110 | UD | E |  | DOLPMTcer\|LdBPK_301910 | UD | N |  | LIPOT\|LdBPK_180990 | UD | N |
| XANtg\|LdBPK_271970 | UD | E |  | DOLPMTcer\|LdBPK_301920 | UD | N |  | LPS\|LdBPK_140520 | UD | N |
| XANtg\|LdBPK_280980 | UD | E |  | DOLPMTcer\|LdBPK_332470 | UD | N |  | LPS\|LdBPK_242340 | UD | N |
| XMPtg\|LdBPK_365650 | UD | E |  | DOLPs\|LdBPK_261530 | UD | N |  | LSTO1r\|LdBPK_180580 | UD | N |
| XTSN2t\|LdBPK_070210 | UD | E |  | DPMVDx\|LdBPK_270090 | UD | N |  | LSTO1r\|LdBPK_323830 | UD | N |
| XTSN2t\|LdBPK_312650 | UD | E |  | DRBKr\|LdBPK_320940 | UD | N |  | LSTO1r\|LdBPK_332740 | UD | N |
| XTSN2t\|LdBPK_350100 | UD | E |  | DTDPGALtg\|LdBPK_010280 | UD | N |  | LYSPLpe\|LdBPK_333420 | UD | N |
| XTSN2t\|LdBPK_351540 | UD | E |  | DTDPGALtg\|LdBPK_040240 | UD | N |  | LYSTRS\|LdBPK_160960 | UD | N |
| XYLTRED_D\|LdBPK_161340 | UD | E |  | DTDPGALtg\|LdBPK_150410 | UD | N |  | MALCOAter\|LdBPK_303560 | UD | N |
| XYLTRED_D\|LdBPK_355060 | UD | E |  | DTDPGALtg\|LdBPK_260410 | UD | N |  | MALT1\|LdBPK_241880 | UD | N |
| ZYMSTt\|LdBPK_330530 | UD | E |  | DTDPGALtg\|LdBPK_261140 | UD | N |  | MALT1\|LdBPK_242110 | UD | N |
| ZYMSTter\|LdBPK_353280 | UD | E |  | DTDPGALtg\|LdBPK_301530 | UD | N |  | MALT1\|LdBPK_272390 | UD | N |
| TAG6Ptg\|LdBPK_160560 | E | N |  | DTDPGALtg\|LdBPK_301570 | UD | N |  | MAN1ptg\|LdBPK_365780 | UD | N |
| 2H3Mtm\|LdBPK_352200 | N | N |  | DTDPGALtg\|LdBPK_301940 | UD | N |  | MAN6PI\|LdBPK_340630 | UD | N |
| ADE34t\|LdBPK_281070 | N | N |  | DTDPGALtg\|LdBPK_361740 | UD | N |  | MANS\|LdBPK_322070 | UD | N |
| NDPKn7\|LdBPK_362480 | N | N |  | DTMPtm\|LdBPK_151120 | UD | N |  | MANt2\|LdBPK_300630 | UD | N |
| PHACGLYtm\|LdBPK_191590 | N | N |  | DTMPtm\|LdBPK_151140 | UD | N |  | MAT6m\|LdBPK_282600 | UD | N |
| PYRtm\|LdBPK_040440 | N | N |  | DTTPtn\|LdBPK_160030 | UD | N |  | MCMAT3m\|LdBPK_230410 | UD | N |
| UBQtm\|LdBPK_131090 | N | N |  | DTTPtn\|LdBPK_211490 | UD | N |  | MCMAT5m\|LdBPK_262730 | UD | N |
| 13DPGt\|LdBPK_323110 | UD | N |  | DUTPDPm\|LdBPK_353060 | UD | N |  | MCMAT7m\|LdBPK_242110 | UD | N |
| 13DPGt\|LdBPK_353910 | UD | N |  | EBP2r\|LdBPK_270090 | UD | N |  | MCMAT7m\|LdBPK_332740 | UD | N |
| 14BP1P2\|LdBPK_312980 | UD | N |  | ECAPLH\|LdBPK_030190 | UD | N |  | MCOATAm\|LdBPK_283090 | UD | N |
| 1ag3p_tm\|LdBPK_050350 | UD | N |  | ECOAH13g\|LdBPK_180200 | UD | N |  | MDHg\|LdBPK_050180 | UD | N |
| 26D5MCOAtm\|LdBPK_330250 | UD | N |  | ECOAH14g\|LdBPK_332430 | UD | N |  | MDHg\|LdBPK_250020 | UD | N |
| 2H5Htm\|LdBPK_364860 | UD | N |  | ECOAH15g\|LdBPK_230300 | UD | N |  | MDRPD\|LdBPK_060370 | UD | N |
| 34HPPt2m\|LdBPK_231920 | UD | N |  | ECOAH16m\|LdBPK_271230 | UD | N |  | METS\|LdBPK_151490 | UD | N |
| 3ANTHMT\|LdBPK_355030 | UD | N |  | ECOAH2m\|LdBPK_150860 | UD | N |  | METt6\|LdBPK_141400 | UD | N |
| 3DSPHRr\|LdBPK_300650 | UD | N |  | ECOAH2m\|LdBPK_360240 | UD | N |  | METtm\|LdBPK_292910 | UD | N |
| 3HBACOAtm\|LdBPK_210980 | UD | N |  | ECOAH3m\|LdBPK_180440 | UD | N |  | MEVK\|LdBPK_261530 | UD | N |
| 3ISO3COAtm\|LdBPK_260120 | UD | N |  | ECOAH3m\|LdBPK_191380 | UD | N |  | MEVK\|LdBPK_292420 | UD | N |
| 3MBTm\|LdBPK_362670 | UD | N |  | ECOAH5m\|LdBPK_332860 | UD | N |  | MEVK\|LdBPK_350360 | UD | N |
| 3MGCOAtm\|LdBPK_242150 | UD | N |  | ECOAH7m\|LdBPK_332860 | UD | N |  | MGLYCL\|LdBPK_367290 | UD | N |
| 3PGt\|LdBPK_350330 | UD | N |  | ECOAH9m\|LdBPK_131420 | UD | N |  | MGRA\|LdBPK_330530 | UD | N |
| 4COUCOAL\|LdBPK_303160 | UD | N |  | ENO\|LdBPK_100150 | UD | N |  | MI1PP\|LdBPK_180090 | UD | N |
| 4HTHRS\|LdBPK_140710 | UD | N |  | ENO\|LdBPK_130390 | UD | N |  | MI4PP\|LdBPK_171260 | UD | N |
| 4HTHRS\|LdBPK_140740 | UD | N |  | ENO\|LdBPK_242370 | UD | N |  | MMEm\|LdBPK_261600 | UD | N |
| 4HTHRS\|LdBPK_140750 | UD | N |  | ENO\|LdBPK_321620 | UD | N |  | MMMm\|LdBPK_311150 | UD | N |
| 4HTHRS\|LdBPK_140760 | UD | N |  | ENO\|LdBPK_343280 | UD | N |  | MTAP\|LdBPK_151490 | UD | N |
| 5FOTHFtm\|LdBPK_292910 | UD | N |  | EPPP2v\|LdBPK_041170 | UD | N |  | MTHFD\|LdBPK_260310 | UD | N |
| 5MTAt\|LdBPK_353390 | UD | N |  | ETAPCT\|LdBPK_261530 | UD | N |  | MTHFR\|LdBPK_140970 | UD | N |
| 5mtryptatn\|LdBPK_040960 | UD | N |  | ETAPCT\|LdBPK_332740 | UD | N |  | MTHGXLtm\|LdBPK_061340 | UD | N |
| 5mtryptatn\|LdBPK_340120 | UD | N |  | ETAPCT\|LdBPK_361200 | UD | N |  | MTRI\|LdBPK_303290 | UD | N |
| 5mtryptatn\|LdBPK_340130 | UD | N |  | ETHAMK\|LdBPK_010490 | UD | N |  | NADHDH\|LdBPK_351480 | UD | N |
| 7dhchsterolte\|LdBPK_110520 | UD | N |  | ETHAMK\|LdBPK_010510 | UD | N |  | NADK\|LdBPK_240370 | UD | N |
| 7dhchsterolte\|LdBPK_131110 | UD | N |  | ETHAMK\|LdBPK_010520 | UD | N |  | NADK\|LdBPK_350840 | UD | N |
| A1E\|LdBPK_313080 | UD | N |  | ETHAMK\|LdBPK_010540 | UD | N |  | NADPH_CYOR\|LdBPK_141280 | UD | N |
| ACACT1rm\|LdBPK_050970 | UD | N |  | ETHAMK\|LdBPK_010550 | UD | N |  | NADS2i\|LdBPK_240370 | UD | N |
| ACACT3rm\|LdBPK_322780 | UD | N |  | ETHAMK\|LdBPK_030220 | UD | N |  | NADS2i\|LdBPK_350840 | UD | N |
| ACACT4rm\|LdBPK_342710 | UD | N |  | ETHAMK\|LdBPK_130300 | UD | N |  | NADter\|LdBPK_281960 | UD | N |
| ACACT7rm\|LdBPK_352980 | UD | N |  | ETHAMK\|LdBPK_281250 | UD | N |  | NCAMt\|LdBPK_322200 | UD | N |
| ACCOACrm\|LdBPK_340630 | UD | N |  | ETHAPT\|LdBPK_261530 | UD | N |  | NDPK1\|LdBPK_210300 | UD | N |
| accoatm\|LdBPK_281390 | UD | N |  | ETHAPT\|LdBPK_332740 | UD | N |  | NDPK1\|LdBPK_210310 | UD | N |
| ACGAM6PSi\|LdBPK_211120 | UD | N |  | ETHAPT\|LdBPK_361200 | UD | N |  | NDPK10\|LdBPK_210300 | UD | N |
| ACGAM6PSi\|LdBPK_300470 | UD | N |  | EX_5mta_e\|LdBPK_231560 | UD | N |  | NDPK10\|LdBPK_210310 | UD | N |
| ACGAM6Ptg\|LdBPK_323110 | UD | N |  | EX_a_D_glucose_e\|LdBPK_350360 | UD | N |  | NDPK2\|LdBPK_181360 | UD | N |
| ACGAM6Ptg\|LdBPK_353910 | UD | N |  | EX_ac_e\|LdBPK_303230 | UD | N |  | NDPK2\|LdBPK_251790 | UD | N |
| ACGAMPM\|LdBPK_231880 | UD | N |  | EX_AHCYS(e)\|LdBPK_110550 | UD | N |  | NDPK3\|LdBPK_210610 | UD | N |
| ACMAT1m\|LdBPK_361840 | UD | N |  | EX_AHCYS(e)\|LdBPK_140320 | UD | N |  | NDPK3\|LdBPK_362790 | UD | N |
| ACOAD10m\|LdBPK_180440 | UD | N |  | EX_AHCYS(e)\|LdBPK_310600 | UD | N |  | NDPK4\|LdBPK_291950 | UD | N |
| ACOAD10m\|LdBPK_191380 | UD | N |  | EX_AHCYS(e)\|LdBPK_310610 | UD | N |  | NDPK4\|LdBPK_312710 | UD | N |
| ACOAD8m\|LdBPK_362670 | UD | N |  | EX_ala_D_e\|LdBPK_130020 | UD | N |  | NDPK4\|LdBPK_312720 | UD | N |
| ACOAD9m\|LdBPK_180440 | UD | N |  | EX_ala_L_e\|LdBPK_310580 | UD | N |  | NDPK4\|LdBPK_323510 | UD | N |
| ACOAD9m\|LdBPK_191380 | UD | N |  | EX_arg_L_e\|LdBPK_131360 | UD | N |  | NDPK5\|LdBPK_292620 | UD | N |
| ACOAter\|LdBPK_353280 | UD | N |  | EX_asn_L_e\|LdBPK_060670 | UD | N |  | NDPK7\|LdBPK_055450 | UD | N |
| ACPexch\|LdBPK_010050 | UD | N |  | EX_asp_L_e\|LdBPK_130020 | UD | N |  | NDPK9\|LdBPK_230470 | UD | N |
| ACPexch\|LdBPK_280260 | UD | N |  | EX_b_D_glucose_e\|LdBPK_355300 | UD | N |  | NDPK9\|LdBPK_350990 | UD | N |
| ACRCOAtm\|LdBPK_260120 | UD | N |  | EX_btn_e\|LdBPK_190940 | UD | N |  | NDPKn10\|LdBPK_343160 | UD | N |
| ACSm\|LdBPK_300170 | UD | N |  | EX_btn_e\|LdBPK_190970 | UD | N |  | NDPKn10\|LdBPK_351000 | UD | N |
| ACt6\|LdBPK_151050 | UD | N |  | EX_ca2_e\|LdBPK_242200 | UD | N |  | NDPKn3\|LdBPK_141240 | UD | N |
| ACt6\|LdBPK_241700 | UD | N |  | EX_co2_e\|LdBPK_151510 | UD | N |  | NDPKn3\|LdBPK_323910 | UD | N |
| ACtm\|LdBPK_160760 | UD | N |  | EX_cys_L_e\|LdBPK_111100 | UD | N |  | NDPKn5\|LdBPK_361320 | UD | N |
| ADAer\|LdBPK_353070 | UD | N |  | EX_dAMP(e)\|LdBPK_110520 | UD | N |  | NDPKn9\|LdBPK_302990 | UD | N |
| ADAm\|LdBPK_353070 | UD | N |  | EX_dAMP(e)\|LdBPK_131110 | UD | N |  | NDPKn9\|LdBPK_303000 | UD | N |
| ADE34t\|LdBPK_030090 | UD | N |  | EX_dCMP(e)\|LdBPK_110520 | UD | N |  | NDPKn9\|LdBPK_354810 | UD | N |
| ADE34t\|LdBPK_030720 | UD | N |  | EX_dCMP(e)\|LdBPK_131110 | UD | N |  | NH4tm\|LdBPK_230310 | UD | N |
| ADE34t\|LdBPK_041140 | UD | N |  | EX_dGMP(e)\|LdBPK_060310 | UD | N |  | NICRNTK\|LdBPK_030190 | UD | N |
| ADE34t\|LdBPK_120620 | UD | N |  | EX_dGMP(e)\|LdBPK_100360 | UD | N |  | NNAM\|LdBPK_283140 | UD | N |
| ADE34t\|LdBPK_200850 | UD | N |  | EX_dGMP(e)\|LdBPK_100370 | UD | N |  | NOS\|LdBPK_030190 | UD | N |
| ADE34t\|LdBPK_212080 | UD | N |  | EX_dGMP(e)\|LdBPK_100380 | UD | N |  | NPHPPH\|LdBPK_340630 | UD | N |
| ADE34t\|LdBPK_230420 | UD | N |  | EX_dGMP(e)\|LdBPK_100390 | UD | N |  | NTRLASE4\|LdBPK_181220 | UD | N |
| ADE34t\|LdBPK_251170 | UD | N |  | EX_dGMP(e)\|LdBPK_100400 | UD | N |  | NTRLASE4\|LdBPK_181230 | UD | N |
| ADE34t\|LdBPK_261690 | UD | N |  | EX_dGMP(e)\|LdBPK_100410 | UD | N |  | O2t\|LdBPK_251980 | UD | N |
| ADE34t\|LdBPK_282880 | UD | N |  | EX_dGMP(e)\|LdBPK_100420 | UD | N |  | OCCOADm\|LdBPK_120255 | UD | N |
| ADE34t\|LdBPK_311600 | UD | N |  | EX_dGMP(e)\|LdBPK_190870 | UD | N |  | OCDMAT8m8\|LdBPK_180510 | UD | N |
| ADE34t\|LdBPK_367350 | UD | N |  | EX_dGMP(e)\|LdBPK_241810 | UD | N |  | OCMAT3m\|LdBPK_261530 | UD | N |
| ADK1\|LdBPK_303120 | UD | N |  | EX_dGMP(e)\|LdBPK_355160 | UD | N |  | OCMAT3m\|LdBPK_361200 | UD | N |
| ADK1g\|LdBPK_110100 | UD | N |  | EX_DOLP(e)\|LdBPK_110550 | UD | N |  | OCMAT3m3\|LdBPK_350850 | UD | N |
| ADMDCi\|LdBPK_312410 | UD | N |  | EX_DOLP(e)\|LdBPK_140320 | UD | N |  | OCMAT3m3\|LdBPK_351200 | UD | N |
| ADN1t\|LdBPK_050500 | UD | N |  | EX_DOLP(e)\|LdBPK_310600 | UD | N |  | ODH1mi\|LdBPK_221210 | UD | N |
| ADN1t\|LdBPK_050510 | UD | N |  | EX_DOLP(e)\|LdBPK_310610 | UD | N |  | ODH1mi\|LdBPK_281430 | UD | N |
| ADN1t\|LdBPK_052580 | UD | N |  | EX_drib_e\|LdBPK_221210 | UD | N |  | ODHmi\|LdBPK_221210 | UD | N |
| ADN1t\|LdBPK_210820 | UD | N |  | EX_dTTP(e)\|LdBPK_110550 | UD | N |  | ODHmi\|LdBPK_281430 | UD | N |
| ADN1t\|LdBPK_212140 | UD | N |  | EX_dTTP(e)\|LdBPK_140320 | UD | N |  | OHPHM\|LdBPK_302360 | UD | N |
| ADN1t\|LdBPK_240640 | UD | N |  | EX_dTTP(e)\|LdBPK_310600 | UD | N |  | OMPDCg\|LdBPK_353060 | UD | N |
| ADN1t\|LdBPK_251210 | UD | N |  | EX_dTTP(e)\|LdBPK_310610 | UD | N |  | ORNtg\|LdBPK_323110 | UD | N |
| ADN1t\|LdBPK_303660 | UD | N |  | EX_dUTP(e)\|LdBPK_110550 | UD | N |  | ORNtg\|LdBPK_353910 | UD | N |
| ADNK1er\|LdBPK_010500 | UD | N |  | EX_dUTP(e)\|LdBPK_140320 | UD | N |  | OROTtg\|LdBPK_211450 | UD | N |
| ADNK1m\|LdBPK_140350 | UD | N |  | EX_dUTP(e)\|LdBPK_310600 | UD | N |  | ORPTg\|LdBPK_271940 | UD | N |
| ADPT2\|LdBPK_140350 | UD | N |  | EX_dUTP(e)\|LdBPK_310610 | UD | N |  | ORPTg\|LdBPK_290290 | UD | N |
| ADSL1r\|LdBPK_351420 | UD | N |  | EX_etha_e\|LdBPK_190940 | UD | N |  | OXADPCOAtg\|LdBPK_181580 | UD | N |
| ADSS_i\|LdBPK_353070 | UD | N |  | EX_etha_e\|LdBPK_190970 | UD | N |  | OXADPCOAtm\|LdBPK_210980 | UD | N |
| AGMT\|LdBPK_080210 | UD | N |  | EX_fol_e\|LdBPK_310580 | UD | N |  | P5CDr\|LdBPK_230860 | UD | N |
| AGPATi\|LdBPK_261530 | UD | N |  | EX_h_e\|LdBPK_313050 | UD | N |  | PALMER\|LdBPK_366570 | UD | N |
| AGPATi\|LdBPK_292420 | UD | N |  | EX_h2o_e\|LdBPK_190940 | UD | N |  | PAPAm\|LdBPK_367290 | UD | N |
| AGPATi\|LdBPK_323830 | UD | N |  | EX_h2o_e\|LdBPK_190970 | UD | N |  | PCter\|LdBPK_321750 | UD | N |
| AGPATi\|LdBPK_332740 | UD | N |  | EX_hxan_e\|LdBPK_362510 | UD | N |  | PCtm\|LdBPK_332040 | UD | N |
| AGPATi\|LdBPK_350360 | UD | N |  | EX_hxan_e\|LdBPK_362520 | UD | N |  | PCtm\|LdBPK_365630 | UD | N |
| AGPATi\|LdBPK_363440 | UD | N |  | EX_inost_e\|LdBPK_190940 | UD | N |  | PDHe2\|LdBPK_140660 | UD | N |
| AGPATim\|LdBPK_261530 | UD | N |  | EX_inost_e\|LdBPK_190970 | UD | N |  | PDHe2\|LdBPK_140670 | UD | N |
| AGPATim\|LdBPK_292420 | UD | N |  | EX_mannan(e)\|LdBPK_310900 | UD | N |  | PDHe2\|LdBPK_140680 | UD | N |
| AGPATim\|LdBPK_323830 | UD | N |  | EX_mannan(e)\|LdBPK_310910 | UD | N |  | PEPt\|LdBPK_211450 | UD | N |
| AGPATim\|LdBPK_332740 | UD | N |  | EX_met_L_e\|LdBPK_161410 | UD | N |  | PERLLALDtm\|LdBPK_271770 | UD | N |
| AGPATim\|LdBPK_350360 | UD | N |  | EX_nh4_e\|LdBPK_355300 | UD | N |  | PERLLAtm\|LdBPK_230050 | UD | N |
| AGPATim\|LdBPK_363440 | UD | N |  | EX_PELDo(e)\|LdBPK_110550 | UD | N |  | PFK26\|LdBPK_340630 | UD | N |
| AHC\|LdBPK_303290 | UD | N |  | EX_PELDo(e)\|LdBPK_140320 | UD | N |  | PFKg\|LdBPK_140660 | UD | N |
| AHCYSter\|LdBPK_210890 | UD | N |  | EX_PELDo(e)\|LdBPK_310600 | UD | N |  | PFKg\|LdBPK_140670 | UD | N |
| AHDTtn\|LdBPK_323110 | UD | N |  | EX_PELDo(e)\|LdBPK_310610 | UD | N |  | PFKg\|LdBPK_140680 | UD | N |
| AHDTtn\|LdBPK_353910 | UD | N |  | EX_pi_e\|LdBPK_303230 | UD | N |  | PGI1\|LdBPK_060890 | UD | N |
| AHSERL5i\|LdBPK_261530 | UD | N |  | EX_ppi_e\|LdBPK_270090 | UD | N |  | PGI3\|LdBPK_060890 | UD | N |
| AHSERL5i\|LdBPK_292420 | UD | N |  | EX_pro_L_e\|LdBPK_313050 | UD | N |  | PGKg\|LdBPK_060890 | UD | N |
| AHSERL5i\|LdBPK_350360 | UD | N |  | EX_ptrc(e)\|LdBPK_110550 | UD | N |  | PGL\|LdBPK_320940 | UD | N |
| AKGDe1\|LdBPK_191490 | UD | N |  | EX_ptrc(e)\|LdBPK_140320 | UD | N |  | PGLYPtm\|LdBPK_281390 | UD | N |
| AKGDE2B\|LdBPK_270090 | UD | N |  | EX_ptrc(e)\|LdBPK_310600 | UD | N |  | PGMT\|LdBPK_060890 | UD | N |
| ALA_Lt6\|LdBPK_366670 | UD | N |  | EX_ptrc(e)\|LdBPK_310610 | UD | N |  | PGPPH\|LdBPK_010490 | UD | N |
| ALATA_L\|LdBPK_230860 | UD | N |  | EX_pyr_e\|LdBPK_151510 | UD | N |  | PGPPH\|LdBPK_010510 | UD | N |
| ALATRSi\|LdBPK_260310 | UD | N |  | EX_thym_e\|LdBPK_231560 | UD | N |  | PGPPH\|LdBPK_010520 | UD | N |
| ALCD19\|LdBPK_230860 | UD | N |  | EX_tyr_L_e\|LdBPK_221210 | UD | N |  | PGPPH\|LdBPK_010540 | UD | N |
| ALDD19m\|LdBPK_040300 | UD | N |  | EX_urea_e\|LdBPK_262240 | UD | N |  | PGPPH\|LdBPK_010550 | UD | N |
| ALDD19m\|LdBPK_040310 | UD | N |  | EX_zymst_e\|LdBPK_190940 | UD | N |  | PGPPH\|LdBPK_030220 | UD | N |
| ALDD19m\|LdBPK_231070 | UD | N |  | EX_zymst_e\|LdBPK_190970 | UD | N |  | PGPPH\|LdBPK_130300 | UD | N |
| ALDD19m\|LdBPK_272290 | UD | N |  | FA_1\|LdBPK_044270 | UD | N |  | PGPPH\|LdBPK_281250 | UD | N |
| ALDD19m\|LdBPK_350640 | UD | N |  | FA_2\|LdBPK_230310 | UD | N |  | PGtm\|LdBPK_080520 | UD | N |
| ALDD20m\|LdBPK_362070 | UD | N |  | FA_3ACPH\|LdBPK_072500 | UD | N |  | PGtm\|LdBPK_081040 | UD | N |
| ALDD32m\|LdBPK_310620 | UD | N |  | FA_3ACPH\|LdBPK_271710 | UD | N |  | PHACCOAtm\|LdBPK_300940 | UD | N |
| ALDD8xr_m\|LdBPK_140660 | UD | N |  | FA_4ACPH\|LdBPK_151050 | UD | N |  | PHACCOAtm\|LdBPK_300950 | UD | N |
| ALDD8xr_m\|LdBPK_140670 | UD | N |  | FA_4ACPH\|LdBPK_241700 | UD | N |  | PHACCOAtm\|LdBPK_343380 | UD | N |
| ALDD8xr_m\|LdBPK_140680 | UD | N |  | FA160ACPH\|LdBPK_190710 | UD | N |  | PHCYTm\|LdBPK_071260 | UD | N |
| ALDOXm\|LdBPK_261670 | UD | N |  | FA180ACPtm\|LdBPK_353730 | UD | N |  | PHE4MOi2\|LdBPK_261600 | UD | N |
| ALDOXm\|LdBPK_261680 | UD | N |  | FA181ACPH\|LdBPK_340140 | UD | N |  | PHEMEtm\|LdBPK_070820 | UD | N |
| ALDR\|LdBPK_241880 | UD | N |  | FA181ACPH\|LdBPK_340150 | UD | N |  | PHETA1\|LdBPK_341170 | UD | N |
| ALDR\|LdBPK_242110 | UD | N |  | FA181ACPH\|LdBPK_340160 | UD | N |  | PHETRS\|LdBPK_282250 | UD | N |
| ALDR\|LdBPK_272390 | UD | N |  | FA181ACPH\|LdBPK_340170 | UD | N |  | PHETRS\|LdBPK_366220 | UD | N |
| ALINCOAe\|LdBPK_262730 | UD | N |  | FA182ACPtm\|LdBPK_331660 | UD | N |  | PI45BPP\|LdBPK_180090 | UD | N |
| AMETter\|LdBPK_366570 | UD | N |  | FA1ACPtm\|LdBPK_282100 | UD | N |  | PIN3K\|LdBPK_040300 | UD | N |
| AMPDg\|LdBPK_060760 | UD | N |  | FACOAL140\|LdBPK_332860 | UD | N |  | PIN3K\|LdBPK_040310 | UD | N |
| APIND\|LdBPK_040300 | UD | N |  | FACOAL180\|LdBPK_332860 | UD | N |  | PIN3K\|LdBPK_231070 | UD | N |
| APIND\|LdBPK_040310 | UD | N |  | FACOAL182\|LdBPK_332860 | UD | N |  | PIN3K\|LdBPK_272290 | UD | N |
| APIND\|LdBPK_231070 | UD | N |  | FAS100COAr\|LdBPK_340630 | UD | N |  | PIN3K\|LdBPK_350640 | UD | N |
| APIND\|LdBPK_272290 | UD | N |  | FAS140COAr\|LdBPK_340630 | UD | N |  | PIN4K\|LdBPK_040300 | UD | N |
| APIND\|LdBPK_350640 | UD | N |  | FAS180COAr\|LdBPK_340630 | UD | N |  | PIN4K\|LdBPK_040310 | UD | N |
| APINM\|LdBPK_252550 | UD | N |  | FAS204COA\|LdBPK_340630 | UD | N |  | PIN4K\|LdBPK_231070 | UD | N |
| ARGDC\|LdBPK_323460 | UD | N |  | FAS224COAr\|LdBPK_340630 | UD | N |  | PIN4K\|LdBPK_272290 | UD | N |
| ARGNg\|LdBPK_282250 | UD | N |  | FAS60COAr\|LdBPK_340630 | UD | N |  | PIN4K\|LdBPK_350640 | UD | N |
| ARGNg\|LdBPK_366220 | UD | N |  | FBP26\|LdBPK_340630 | UD | N |  | PIt2p\|LdBPK_200630 | UD | N |
| ARGSSgr\|LdBPK_030190 | UD | N |  | FBPg\|LdBPK_051160 | UD | N |  | PLAc\|LdBPK_071260 | UD | N |
| ARGtg\|LdBPK_221110 | UD | N |  | FCL\|LdBPK_302100 | UD | N |  | PLAPc\|LdBPK_141420 | UD | N |
| ARGtg\|LdBPK_271970 | UD | N |  | FETe\|LdBPK_262730 | UD | N |  | PMANMg\|LdBPK_241880 | UD | N |
| ARGtg\|LdBPK_280980 | UD | N |  | FOLt\|LdBPK_280140 | UD | N |  | PMANMg\|LdBPK_242110 | UD | N |
| ASAT\|LdBPK_313030 | UD | N |  | FORter\|LdBPK_170300 | UD | N |  | PMANMg\|LdBPK_272390 | UD | N |
| ASCt2\|LdBPK_070330 | UD | N |  | FRDg\|LdBPK_131420 | UD | N |  | PMETM\|LdBPK_101460 | UD | N |
| ASCt2\|LdBPK_110250 | UD | N |  | FRDPtg\|LdBPK_200630 | UD | N |  | PMEVK\|LdBPK_363230 | UD | N |
| ASCt2\|LdBPK_130670 | UD | N |  | FRDPtm\|LdBPK_171580 | UD | N |  | PMEVKx\|LdBPK_190350 | UD | N |
| ASCt2\|LdBPK_270100 | UD | N |  | FTHFLr\|LdBPK_302590 | UD | N |  | PMTCOAe\|LdBPK_061110 | UD | N |
| ASCt2\|LdBPK_292260 | UD | N |  | FUMg\|LdBPK_340070 | UD | N |  | PMTCOAter\|LdBPK_060480 | UD | N |
| ASCt2\|LdBPK_300370 | UD | N |  | FUMm\|LdBPK_361200 | UD | N |  | PNS1\|LdBPK_020470 | UD | N |
| ASCt2\|LdBPK_300620 | UD | N |  | G3PDcm\|LdBPK_261530 | UD | N |  | PNS3\|LdBPK_260040 | UD | N |
| ASCt2\|LdBPK_321900 | UD | N |  | G3PDcm\|LdBPK_332740 | UD | N |  | PNTK\|LdBPK_091100 | UD | N |
| ASCt2\|LdBPK_364460 | UD | N |  | G3PDcm\|LdBPK_361200 | UD | N |  | PNTO_Rte\|LdBPK_322800 | UD | N |
| ASNN\|LdBPK_221390 | UD | N |  | G5SAm_spt\|LdBPK_060350 | UD | N |  | PNTO_Rte\|LdBPK_331510 | UD | N |
| ASNS3\|LdBPK_150440 | UD | N |  | G5SDrm\|LdBPK_330730 | UD | N |  | PNTO_Rte\|LdBPK_355070 | UD | N |
| ASNt6\|LdBPK_354790 | UD | N |  | G6PDAg\|LdBPK_050180 | UD | N |  | PNTO_Rte\|LdBPK_355320 | UD | N |
| ASNt6\|LdBPK_363990 | UD | N |  | G6PDAg\|LdBPK_250020 | UD | N |  | PNTO_Rte\|LdBPK_360450 | UD | N |
| ASNt6\|LdBPK_364000 | UD | N |  | G6PDHg\|LdBPK_366160 | UD | N |  | PNTO_Rte\|LdBPK_367150 | UD | N |
| ASNtg\|LdBPK_240790 | UD | N |  | G6Ptg\|LdBPK_160550 | UD | N |  | PNTOt2\|LdBPK_360060 | UD | N |
| ASPCT\|LdBPK_210700 | UD | N |  | GALKx\|LdBPK_241880 | UD | N |  | PPA_1\|LdBPK_271330 | UD | N |
| ASPTA1m\|LdBPK_230860 | UD | N |  | GALKx\|LdBPK_242110 | UD | N |  | PPA_1\|LdBPK_351480 | UD | N |
| ASPTA4\|LdBPK_340630 | UD | N |  | GALKx\|LdBPK_272390 | UD | N |  | PPATM\|LdBPK_260120 | UD | N |
| ASPtg\|LdBPK_111000 | UD | N |  | GALS3a\|LdBPK_241880 | UD | N |  | PPCDC\|LdBPK_030970 | UD | N |
| ASPTRS\|LdBPK_364650 | UD | N |  | GALS3a\|LdBPK_242110 | UD | N |  | PPCDC\|LdBPK_366160 | UD | N |
| ATPS\|LdBPK_070360 | UD | N |  | GALS3a\|LdBPK_272390 | UD | N |  | PPCKg\|LdBPK_262290 | UD | N |
| ATPSm\|LdBPK_141280 | UD | N |  | GAM6Ptg\|LdBPK_160540 | UD | N |  | PPDKg\|LdBPK_251160 | UD | N |
| BTCOAter\|LdBPK_353280 | UD | N |  | GAMNCt\|LdBPK_050980 | UD | N |  | PPit\|LdBPK_360060 | UD | N |
| BTCOAtm\|LdBPK_240370 | UD | N |  | GAMNCt\|LdBPK_170320 | UD | N |  | PPItg\|LdBPK_160580 | UD | N |
| BTMAT1m\|LdBPK_261530 | UD | N |  | GAMNCt\|LdBPK_181460 | UD | N |  | PPItm\|LdBPK_260030 | UD | N |
| BTMAT1m\|LdBPK_361200 | UD | N |  | GAMNCt\|LdBPK_270300 | UD | N |  | PPItn\|LdBPK_331150 | UD | N |
| BTMAT1m1\|LdBPK_351190 | UD | N |  | GAMNCt\|LdBPK_270590 | UD | N |  | PPItn\|LdBPK_362390 | UD | N |
| C14STRr\|LdBPK_150940 | UD | N |  | GAMNCt\|LdBPK_282170 | UD | N |  | PPM2\|LdBPK_180810 | UD | N |
| C14STRr\|LdBPK_171510 | UD | N |  | GAMNCt\|LdBPK_323370 | UD | N |  | PPM2\|LdBPK_240330 | UD | N |
| C3STDH1r\|LdBPK_150940 | UD | N |  | GAMNCt\|LdBPK_352010 | UD | N |  | PPM2\|LdBPK_366160 | UD | N |
| C3STDH1r\|LdBPK_171510 | UD | N |  | GAMNCtg\|LdBPK_323110 | UD | N |  | PPPGO\|LdBPK_312360 | UD | N |
| C3STDH2r\|LdBPK_302970 | UD | N |  | GAMNCtg\|LdBPK_353910 | UD | N |  | PRODXm\|LdBPK_210300 | UD | N |
| C3STDH2r\|LdBPK_350040 | UD | N |  | GAMNt\|LdBPK_292910 | UD | N |  | PRODXm\|LdBPK_210310 | UD | N |
| C4STMO1r\|LdBPK_044320 | UD | N |  | GAMNtg\|LdBPK_323110 | UD | N |  | PROm\|LdBPK_030190 | UD | N |
| C4STMO1r\|LdBPK_262520 | UD | N |  | GAMNtg\|LdBPK_353910 | UD | N |  | PROTRS\|LdBPK_360040 | UD | N |
| C4STMO1r\|LdBPK_312790 | UD | N |  | GAPD\|LdBPK_100150 | UD | N |  | PRPPSi\|LdBPK_364030 | UD | N |
| C4STMO1r\|LdBPK_350560 | UD | N |  | GAPD\|LdBPK_130390 | UD | N |  | PS_tm\|LdBPK_211490 | UD | N |
| C4STMO1r\|LdBPK_360400 | UD | N |  | GAPD\|LdBPK_242370 | UD | N |  | PSACTROPtm\|LdBPK_141460 | UD | N |
| C5STDSr\|LdBPK_020100 | UD | N |  | GAPD\|LdBPK_321620 | UD | N |  | PSD\|LdBPK_141420 | UD | N |
| C5STDSr\|LdBPK_201140 | UD | N |  | GAPD\|LdBPK_343280 | UD | N |  | PSEUTROPtm\|LdBPK_330250 | UD | N |
| C5STDSr\|LdBPK_242090 | UD | N |  | GAPt\|LdBPK_060580 | UD | N |  | PTD1INOter\|LdBPK_365160 | UD | N |
| C5STDSr\|LdBPK_301840 | UD | N |  | GCCa\|LdBPK_140700 | UD | N |  | PTHK\|LdBPK_030970 | UD | N |
| C5STDSr\|LdBPK_343750 | UD | N |  | GCCb\|LdBPK_181050 | UD | N |  | PTHK\|LdBPK_366160 | UD | N |
| C5STDSr\|LdBPK_344160 | UD | N |  | GDNBT\|LdBPK_060980 | UD | N |  | PTR1a\|LdBPK_353070 | UD | N |
| C8STIr\|LdBPK_343370 | UD | N |  | GK1er\|LdBPK_210300 | UD | N |  | PTRCt\|LdBPK_051140 | UD | N |
| CaATPAC\|LdBPK_341860 | UD | N |  | GK1er\|LdBPK_210310 | UD | N |  | PTRCt\|LdBPK_120480 | UD | N |
| CDAL2\|LdBPK_060910 | UD | N |  | GK1er\|LdBPK_362450 | UD | N |  | PTRCt\|LdBPK_180560 | UD | N |
| CDAL2\|LdBPK_282700 | UD | N |  | GLCBt2\|LdBPK_171450 | UD | N |  | PTRCt\|LdBPK_211590 | UD | N |
| CDAL2\|LdBPK_352780 | UD | N |  | GLCBtg\|LdBPK_170410 | UD | N |  | PTRCt\|LdBPK_212160 | UD | N |
| CDGPTm\|LdBPK_261530 | UD | N |  | GLCBtg\|LdBPK_322210 | UD | N |  | PTRCt\|LdBPK_230150 | UD | N |
| CDGPTm\|LdBPK_292420 | UD | N |  | GLCRtm\|LdBPK_151120 | UD | N |  | PTRCt\|LdBPK_230380 | UD | N |
| CDGPTm\|LdBPK_323830 | UD | N |  | GLCRtm\|LdBPK_151140 | UD | N |  | PTRCt\|LdBPK_281260 | UD | N |
| CDGPTm\|LdBPK_332740 | UD | N |  | GLINCOAtm\|LdBPK_330250 | UD | N |  | PTRCt\|LdBPK_282610 | UD | N |
| CDGPTm\|LdBPK_350360 | UD | N |  | GLNS_i\|LdBPK_060910 | UD | N |  | PTRCt\|LdBPK_303720 | UD | N |
| CDGPTm\|LdBPK_363440 | UD | N |  | GLNS_i\|LdBPK_282700 | UD | N |  | PTRCt\|LdBPK_320970 | UD | N |
| CDPCHOLtm\|LdBPK_242150 | UD | N |  | GLNS_i\|LdBPK_352780 | UD | N |  | PTRCt\|LdBPK_343460 | UD | N |
| CDPDSP\|LdBPK_261530 | UD | N |  | GLNtm\|LdBPK_170870 | UD | N |  | PTRCt\|LdBPK_350710 | UD | N |
| CDPDSP\|LdBPK_292420 | UD | N |  | GLNtn\|LdBPK_323110 | UD | N |  | PTRCt\|LdBPK_363250 | UD | N |
| CDPDSP\|LdBPK_323830 | UD | N |  | GLNtn\|LdBPK_353910 | UD | N |  | PTROPACE\|LdBPK_260790 | UD | N |
| CDPDSP\|LdBPK_332740 | UD | N |  | GLNTRNAtm\|LdBPK_292910 | UD | N |  | PUNP6\|LdBPK_120490 | UD | N |
| CDPDSP\|LdBPK_350360 | UD | N |  | GLNTRS\|LdBPK_060910 | UD | N |  | PUNP8I\|LdBPK_151070 | UD | N |
| CDPDSP\|LdBPK_363440 | UD | N |  | GLNTRS\|LdBPK_282700 | UD | N |  | PYDXK\|LdBPK_161410 | UD | N |
| CDPDSPm\|LdBPK_261530 | UD | N |  | GLNTRS\|LdBPK_352780 | UD | N |  | PYDXNt\|LdBPK_070700 | UD | N |
| CDPDSPm\|LdBPK_292420 | UD | N |  | GLNTRSm_i\|LdBPK_060910 | UD | N |  | PYDXNt\|LdBPK_070720 | UD | N |
| CDPDSPm\|LdBPK_323830 | UD | N |  | GLNTRSm_i\|LdBPK_282700 | UD | N |  | PYDXt\|LdBPK_221380 | UD | N |
| CDPDSPm\|LdBPK_332740 | UD | N |  | GLNTRSm_i\|LdBPK_352780 | UD | N |  | PYRt2\|LdBPK_340080 | UD | N |
| CDPDSPm\|LdBPK_350360 | UD | N |  | GLU5K\|LdBPK_322470 | UD | N |  | QDPR\|LdBPK_262500 | UD | N |
| CDPDSPm\|LdBPK_363440 | UD | N |  | GLUCYSL\|LdBPK_060910 | UD | N |  | RAFFH\|LdBPK_241880 | UD | N |
| CDPtn\|LdBPK_331150 | UD | N |  | GLUCYSL\|LdBPK_282700 | UD | N |  | RAFFH\|LdBPK_242110 | UD | N |
| CDPtn\|LdBPK_362390 | UD | N |  | GLUCYSL\|LdBPK_352780 | UD | N |  | RAFFH\|LdBPK_272390 | UD | N |
| CH4tn\|LdBPK_323110 | UD | N |  | GLUDx_M\|LdBPK_230860 | UD | N |  | RBK_Dg\|LdBPK_161340 | UD | N |
| CH4tn\|LdBPK_353910 | UD | N |  | GLUt6\|LdBPK_231270 | UD | N |  | RBK_Dg\|LdBPK_355060 | UD | N |
| CHLPCTD\|LdBPK_261530 | UD | N |  | GLUTCOAtm\|LdBPK_292910 | UD | N |  | RBLLtg\|LdBPK_060890 | UD | N |
| CHLPCTD\|LdBPK_292420 | UD | N |  | GLUTRS\|LdBPK_060910 | UD | N |  | RNDR1_n\|LdBPK_120490 | UD | N |
| CHLPCTD\|LdBPK_323830 | UD | N |  | GLUTRS\|LdBPK_282700 | UD | N |  | RNDR3_n\|LdBPK_200120 | UD | N |
| CHLPCTD\|LdBPK_332740 | UD | N |  | GLUTRS\|LdBPK_352780 | UD | N |  | RNDR4_n\|LdBPK_200110 | UD | N |
| CHLPCTD\|LdBPK_350360 | UD | N |  | GLUTRSm\|LdBPK_060910 | UD | N |  | RPE\|LdBPK_180810 | UD | N |
| CHLPCTD\|LdBPK_363440 | UD | N |  | GLUTRSm\|LdBPK_282700 | UD | N |  | RPE\|LdBPK_240330 | UD | N |
| CHLt6\|LdBPK_110210 | UD | N |  | GLUTRSm\|LdBPK_352780 | UD | N |  | RPE\|LdBPK_366160 | UD | N |
| CHOLK\|LdBPK_261530 | UD | N |  | GLYALDtm\|LdBPK_252480 | UD | N |  | RPEg\|LdBPK_200500 | UD | N |
| CHOLK\|LdBPK_292420 | UD | N |  | GLYALDtm\|LdBPK_361410 | UD | N |  | RPEg\|LdBPK_280240 | UD | N |
| CHOLK\|LdBPK_323830 | UD | N |  | GLYCt5\|LdBPK_311240 | UD | N |  | RU5PLtg\|LdBPK_323110 | UD | N |
| CHOLK\|LdBPK_332740 | UD | N |  | GLYCtg\|LdBPK_323110 | UD | N |  | RU5PLtg\|LdBPK_353910 | UD | N |
| CHOLK\|LdBPK_350360 | UD | N |  | GLYCtg\|LdBPK_353910 | UD | N |  | Ru5ptg\|LdBPK_221110 | UD | N |
| CHOLK\|LdBPK_363440 | UD | N |  | GLYKg\|LdBPK_230860 | UD | N |  | Ru5ptg\|LdBPK_271970 | UD | N |
| CLPNSm\|LdBPK_010490 | UD | N |  | GLYOX1\|LdBPK_230120 | UD | N |  | Ru5ptg\|LdBPK_280980 | UD | N |
| CLPNSm\|LdBPK_010510 | UD | N |  | GLYt6\|LdBPK_060890 | UD | N |  | S6PFH\|LdBPK_241880 | UD | N |
| CLPNSm\|LdBPK_010520 | UD | N |  | GLYt6\|LdBPK_230310 | UD | N |  | S6PFH\|LdBPK_242110 | UD | N |
| CLPNSm\|LdBPK_010540 | UD | N |  | GLYTm\|LdBPK_300940 | UD | N |  | S6PFH\|LdBPK_272390 | UD | N |
| CLPNSm\|LdBPK_010550 | UD | N |  | GLYTm\|LdBPK_300950 | UD | N |  | S7Ptg\|LdBPK_323110 | UD | N |
| CLPNSm\|LdBPK_030220 | UD | N |  | GLYTm\|LdBPK_343380 | UD | N |  | S7Ptg\|LdBPK_353910 | UD | N |
| CLPNSm\|LdBPK_130300 | UD | N |  | GMPS2\|LdBPK_354660 | UD | N |  | SAM24MTr\|LdBPK_190350 | UD | N |
| CLPNSm\|LdBPK_281250 | UD | N |  | GMPter\|LdBPK_050830 | UD | N |  | SBPP1r\|LdBPK_313030 | UD | N |
| CLPNte\|LdBPK_330310 | UD | N |  | GPAM\|LdBPK_261530 | UD | N |  | SELPS\|LdBPK_030580 | UD | N |
| CLPNte\|LdBPK_366560 | UD | N |  | GPAM\|LdBPK_332740 | UD | N |  | SELPS\|LdBPK_120850 | UD | N |
| CLPNtm\|LdBPK_242150 | UD | N |  | GPAM\|LdBPK_361200 | UD | N |  | SERD_L\|LdBPK_321220 | UD | N |
| CO2t\|LdBPK_340080 | UD | N |  | GPDDA2\|LdBPK_261530 | UD | N |  | SERPTr\|LdBPK_111010 | UD | N |
| CO2tm\|LdBPK_330250 | UD | N |  | GPDDA2\|LdBPK_332740 | UD | N |  | SERter\|LdBPK_310010 | UD | N |
| CO2tp\|LdBPK_160560 | UD | N |  | GPDDA2\|LdBPK_361200 | UD | N |  | SERtm\|LdBPK_100270 | UD | N |
| COAter\|LdBPK_353280 | UD | N |  | GRTT\|LdBPK_050180 | UD | N |  | SERtm\|LdBPK_110220 | UD | N |
| COAtg\|LdBPK_323110 | UD | N |  | GRTT\|LdBPK_250020 | UD | N |  | SGPL12r\|LdBPK_150940 | UD | N |
| COAtg\|LdBPK_353910 | UD | N |  | GSADHm\|LdBPK_332650 | UD | N |  | SGPL12r\|LdBPK_171510 | UD | N |
| COAtm\|LdBPK_242150 | UD | N |  | GSN2t\|LdBPK_342500 | UD | N |  | SHMT\|LdBPK_353130 | UD | N |
| CPPPGO\|LdBPK_313250 | UD | N |  | GSS\|LdBPK_310860 | UD | N |  | SHSL1\|LdBPK_181660 | UD | N |
| CSm\|LdBPK_131420 | UD | N |  | GSS\|LdBPK_310870 | UD | N |  | SHSL4r\|LdBPK_120580 | UD | N |
| CTL2\|LdBPK_240370 | UD | N |  | GSS\|LdBPK_312540 | UD | N |  | SHSL4r\|LdBPK_350840 | UD | N |
| CTL2\|LdBPK_350840 | UD | N |  | GTHS\|LdBPK_230860 | UD | N |  | SINCOAL\|LdBPK_140470 | UD | N |
| CTPS2n\|LdBPK_210700 | UD | N |  | GUAt2\|LdBPK_354790 | UD | N |  | SPHK21r\|LdBPK_111010 | UD | N |
| CTPtm\|LdBPK_282880 | UD | N |  | GUAt2\|LdBPK_363990 | UD | N |  | SPMDte\|LdBPK_330310 | UD | N |
| CTPtn\|LdBPK_010310 | UD | N |  | GUAt2\|LdBPK_364000 | UD | N |  | SPMDte\|LdBPK_366560 | UD | N |
| CYOO6m\|LdBPK_181310 | UD | N |  | H2St\|LdBPK_030890 | UD | N |  | SPMS\|LdBPK_310860 | UD | N |
| CYSDS\|LdBPK_311070 | UD | N |  | H3MS\|LdBPK_302360 | UD | N |  | SPMS\|LdBPK_310870 | UD | N |
| CYSt6\|LdBPK_354790 | UD | N |  | HACD1m\|LdBPK_332860 | UD | N |  | SPMS\|LdBPK_312540 | UD | N |
| CYSt6\|LdBPK_363990 | UD | N |  | HACD3m\|LdBPK_332860 | UD | N |  | SQer\|LdBPK_363230 | UD | N |
| CYSt6\|LdBPK_364000 | UD | N |  | HACD5m\|LdBPK_332860 | UD | N |  | SQLS\|LdBPK_150270 | UD | N |
| CYSTRS\|LdBPK_261530 | UD | N |  | HACD8m\|LdBPK_230300 | UD | N |  | SQLS\|LdBPK_300130 | UD | N |
| CYSTRS\|LdBPK_292420 | UD | N |  | HACD9m\|LdBPK_030190 | UD | N |  | SQLter\|LdBPK_364100 | UD | N |
| CYSTRS\|LdBPK_350360 | UD | N |  | HBCOADm\|LdBPK_030190 | UD | N |  | SRTNtn\|LdBPK_323110 | UD | N |
| CYSTS\|LdBPK_240370 | UD | N |  | HBCOAE\|LdBPK_131420 | UD | N |  | SRTNtn\|LdBPK_353910 | UD | N |
| CYSTS\|LdBPK_350840 | UD | N |  | HBUHL1m\|LdBPK_261530 | UD | N |  | ST14DMr\|LdBPK_282600 | UD | N |
| CYT1t\|LdBPK_040010 | UD | N |  | HBUHL1m\|LdBPK_361200 | UD | N |  | STRCOAtg\|LdBPK_221110 | UD | N |
| CYT1t\|LdBPK_331060 | UD | N |  | HCO3Em\|LdBPK_322070 | UD | N |  | STRCOAtg\|LdBPK_271970 | UD | N |
| CYTK1\|LdBPK_120490 | UD | N |  | HCYSMT\|LdBPK_160590 | UD | N |  | STRCOAtg\|LdBPK_280980 | UD | N |
| CYTK1\|LdBPK_343160 | UD | N |  | HDCOADm\|LdBPK_363750 | UD | N |  | STRCOAtm\|LdBPK_351490 | UD | N |
| CYTK1n\|LdBPK_080060 | UD | N |  | HDDHL5m\|LdBPK_180580 | UD | N |  | Succoatm\|LdBPK_300940 | UD | N |
| CYTK1n\|LdBPK_282380 | UD | N |  | HDDHL5m\|LdBPK_323830 | UD | N |  | Succoatm\|LdBPK_300950 | UD | N |
| CYTK1n\|LdBPK_332230 | UD | N |  | HDDHL5m\|LdBPK_332740 | UD | N |  | Succoatm\|LdBPK_343380 | UD | N |
| CYTK1n\|LdBPK_364270 | UD | N |  | HDDR5m\|LdBPK_270740 | UD | N |  | SUCCt6\|LdBPK_280140 | UD | N |
| CYTK1n\|LdBPK_366960 | UD | N |  | HDDR5m\|LdBPK_363630 | UD | N |  | SUCCtg\|LdBPK_100040 | UD | N |
| D_LACt2\|LdBPK_301550 | UD | N |  | HDMAT7m\|LdBPK_252220 | UD | N |  | SUCOGDPm\|LdBPK_261530 | UD | N |
| DADAr\|LdBPK_030030 | UD | N |  | HDMAT7m\|LdBPK_252230 | UD | N |  | SUCOGDPm\|LdBPK_292420 | UD | N |
| DADK\|LdBPK_070420 | UD | N |  | HDMAT7m\|LdBPK_363100 | UD | N |  | SUCOGDPm\|LdBPK_350360 | UD | N |
| DADKg\|LdBPK_010500 | UD | N |  | HDMAT7m7\|LdBPK_363750 | UD | N |  | T2M26DCOAtm\|LdBPK_060910 | UD | N |
| DAGAT\|LdBPK_010490 | UD | N |  | HEMEASm\|LdBPK_313250 | UD | N |  | T2M26DCOAtm\|LdBPK_070150 | UD | N |
| DAGAT\|LdBPK_010510 | UD | N |  | hemeAtm\|LdBPK_300940 | UD | N |  | T2M26DCOAtm\|LdBPK_282700 | UD | N |
| DAGAT\|LdBPK_010520 | UD | N |  | hemeAtm\|LdBPK_300950 | UD | N |  | T2M26DCOAtm\|LdBPK_352780 | UD | N |
| DAGAT\|LdBPK_010540 | UD | N |  | hemeAtm\|LdBPK_343380 | UD | N |  | TAL\|LdBPK_100560 | UD | N |
| DAGAT\|LdBPK_010550 | UD | N |  | Her\|LdBPK_353280 | UD | N |  | TCAFCOAL\|LdBPK_140470 | UD | N |
| DAGAT\|LdBPK_030220 | UD | N |  | HEX4g\|LdBPK_140770 | UD | N |  | TCINCOAL\|LdBPK_170300 | UD | N |
| DAGAT\|LdBPK_130300 | UD | N |  | HEX4g\|LdBPK_140790 | UD | N |  | TDCOAtg\|LdBPK_323110 | UD | N |
| DAGAT\|LdBPK_281250 | UD | N |  | HEX4g\|LdBPK_140800 | UD | N |  | TDCOAtg\|LdBPK_353910 | UD | N |
| DAGCPT\|LdBPK_010490 | UD | N |  | HEXg\|LdBPK_051160 | UD | N |  | TDPGDH\|LdBPK_200980 | UD | N |
| DAGCPT\|LdBPK_010510 | UD | N |  | HEXNCg\|LdBPK_342110 | UD | N |  | TDPGDH\|LdBPK_344230 | UD | N |
| DAGCPT\|LdBPK_010520 | UD | N |  | HHDHL7m\|LdBPK_261530 | UD | N |  | THFAT\|LdBPK_100430 | UD | N |
| DAGCPT\|LdBPK_010540 | UD | N |  | HHDHL7m\|LdBPK_292420 | UD | N |  | THFOCi\|LdBPK_340630 | UD | N |
| DAGCPT\|LdBPK_010550 | UD | N |  | HHDHL7m\|LdBPK_350360 | UD | N |  | THFtm\|LdBPK_191010 | UD | N |
| DAGCPT\|LdBPK_030220 | UD | N |  | HHDR7m\|LdBPK_292080 | UD | N |  | THFtm\|LdBPK_320920 | UD | N |
| DAGCPT\|LdBPK_130300 | UD | N |  | HIBHrm\|LdBPK_292250 | UD | N |  | THRLAD\|LdBPK_140710 | UD | N |
| THRLAD\|LdBPK_140760 | UD | N |  | THRt6\|LdBPK_181490 | UD | N |  |  |  |  |
| THRLAD\|LdBPK_140740 | UD | N |  | THRLAD\|LdBPK_140750 | UD | N |  |  |  |  |
