# Supplementary material for: Essential gene prediction using limited gene essentiality information–An integrative semi-supervised machine learning strategy
Source: PLoS One. 2020 Nov 30;15(11):e0242943. doi: 10.1371/journal.pone.0242943 (PMC7703937; doi:10.1371/journal.pone.0242943)
Supplement: S6 Table. Gene essentiality information of reaction gene combinations in Leishmania major predicted using the proposed pipeline — (DOCX) [file pone.0242943.s010.docx]

**S6 Table. Gene essentiality information of Reaction Gene combinations in *Leishmania major* predicted using our proposed pipeline**

| **Abbreviation** |
| --- |
| E, Essential gene |
| N, Non-essential gene |
| UD, Undetermined (Not Known) |

^#^Experimental Gene Essentiality Information ( Jones,N.G. et al. (2018) ACS Infect. Dis., 4, 467–477.)

| **GeneReaction** | **Experiment^#^** | **Predicted** |  | **GeneReaction** | **Experiment^#^** | **Predicted** |  | **GeneReaction** | **Experiment^#^** | **Predicted** |
| --- | --- | --- | --- | --- | --- | --- | --- | --- | --- | --- |
| MTHFD\|LmjF.26.0320 | E | E |  | ACOAD8m\|LmjF.27.0930 | UD | N |  | FACOAL180\|LmjF.01.0510 | UD | N |
| DHFOR2\|LmjF.06.0860 | E | E |  | ACOAD9m\|LmjF.06.0880 | UD | N |  | FACOAL181\|LmjF.13.0420 | UD | N |
| DHFOR2a\|LmjF.06.0860 | E | E |  | ACOAD9m\|LmjF.28.2510 | UD | N |  | FACOAL181\|LmjF.03.0230 | UD | N |
| DHFR\|LmjF.06.0860 | E | E |  | ACODA\|LmjF.07.0270 | UD | N |  | FACOAL181\|LmjF.01.0530 | UD | N |
| DHFRa\|LmjF.06.0860 | E | E |  | ACODA\|LmjF.20.1560 | UD | N |  | FACOAL181\|LmjF.01.0490 | UD | N |
| ADNCYC\|LmjF.28.0090 | E | E |  | ACODA\|LmjF.30.1380 | UD | N |  | FACOAL181\|LmjF.01.0510 | UD | N |
| GUACYC\|LmjF.28.0090 | E | E |  | ACODA\|LmjF.31.1810 | UD | N |  | FACOAL181\|LmjF.01.0470 | UD | N |
| MTHFC\|LmjF.26.0320 | E | E |  | ACODA\|LmjF.20.1550 | UD | N |  | FACOAL181\|LmjF.01.0520 | UD | N |
| PIN3K_LM\|LmjF.34.4530 | E | E |  | ACODA\|LmjF.20.1570 | UD | N |  | FACOAL181\|LmjF.01.0500 | UD | N |
| THFOAi\|LmjF.06.0860 | E | E |  | ACODA\|LmjF.31.1130 | UD | N |  | FACOAL182\|LmjF.01.0520 | UD | N |
| THFOCi\|LmjF.06.0860 | E | E |  | ACONTm\|LmjF.18.0510 | UD | N |  | FACOAL182\|LmjF.01.0510 | UD | N |
| TMDS\|LmjF.06.0860 | E | E |  | ACS2m\|LmjF.23.0710 | UD | N |  | FACOAL182\|LmjF.01.0470 | UD | N |
| TRYR\|LmjF.05.0350 | E | E |  | ACS2m\|LmjF.23.0540 | UD | N |  | FACOAL182\|LmjF.01.0500 | UD | N |
| TYRTRS\|LmjF.14.1370 | E | E |  | ACSm\|LmjF.23.0710 | UD | N |  | FACOAL182\|LmjF.13.0420 | UD | N |
| TYRTRS\|LmjF.14.1370 | E | E |  | ACSm\|LmjF.23.0540 | UD | N |  | FACOAL182\|LmjF.01.0490 | UD | N |
| NDPKn5\|LmjF.32.2950 | UD | E |  | ADA\|LmjF.35.2160 | UD | N |  | FACOAL182\|LmjF.03.0230 | UD | N |
| NDPKn6\|LmjF.35.3870 | UD | E |  | ADA\|LmjF.33.0235 | UD | N |  | FACOAL182\|LmjF.01.0530 | UD | N |
| NDPKn6\|LmjF.32.2950 | UD | E |  | ADAer\|LmjF.33.0235 | UD | N |  | FACOAL2\|LmjF.01.0520 | UD | N |
| NDPKn7\|LmjF.32.2950 | UD | E |  | ADAer\|LmjF.35.2160 | UD | N |  | FACOAL2\|LmjF.01.0470 | UD | N |
| NDPKn7\|LmjF.35.3870 | UD | E |  | ADAg\|LmjF.35.2160 | UD | N |  | FACOAL2\|LmjF.01.0510 | UD | N |
| NDPKn8\|LmjF.32.2950 | UD | E |  | ADAg\|LmjF.33.0235 | UD | N |  | FACOAL2\|LmjF.01.0500 | UD | N |
| NDPKn8\|LmjF.35.3870 | UD | E |  | ADAm\|LmjF.33.0235 | UD | N |  | FACOAL2\|LmjF.01.0490 | UD | N |
| NDPKn9\|LmjF.35.3870 | UD | E |  | ADAm\|LmjF.35.2160 | UD | N |  | FACOAL2\|LmjF.03.0230 | UD | N |
| NDPKn9\|LmjF.32.2950 | UD | E |  | ADK1\|LmjF.34.0110 | UD | N |  | FACOAL2\|LmjF.01.0530 | UD | N |
| NICRNTK\|LmjF.29.2150 | UD | E |  | ADK1\|LmjF.34.0120 | UD | N |  | FACOAL2\|LmjF.13.0420 | UD | N |
| NICRNTK\|LmjF.30.0600 | UD | E |  | ADK1\|LmjF.04.0960 | UD | N |  | FAS100COAr\|LmjF.14.0640 | UD | N |
| NICRNTK\|LmjF.30.0370 | UD | E |  | ADK1f\|LmjF.21.1250 | UD | N |  | FAS100COAr\|LmjF.14.0650 | UD | N |
| NICRNTK\|LmjF.32.1810 | UD | E |  | ADK1f\|LmjF.16.0020 | UD | N |  | FAS100COAr\|LmjF.14.0660 | UD | N |
| NICRNTK\|LmjF.07.0170 | UD | E |  | ADK1g\|LmjF.36.1360 | UD | N |  | FAS120COAr\|LmjF.14.0670 | UD | N |
| NICRNTK\|LmjF.11.0250 | UD | E |  | ADK1g\|LmjF.25.2370 | UD | N |  | FAS140COAr\|LmjF.14.0670 | UD | N |
| NICRNTK\|LmjF.27.0100 | UD | E |  | ADMDCi\|LmjF.30.3120 | UD | N |  | FAS160COAr\|LmjF.14.0680 | UD | N |
| NICRNTK\|LmjF.13.0780 | UD | E |  | ADMDCi\|LmjF.30.3110 | UD | N |  | FAS160COAr\|LmjF.14.0700 | UD | N |
| NICRNTK\|LmjF.36.4250 | UD | E |  | ADNCYC\|LmjF.17.0190 | UD | N |  | FAS160COAr\|LmjF.14.0705 | UD | N |
| NO3R\|LmjF.30.0610 | UD | E |  | ADNCYC\|LmjF.17.0235 | UD | N |  | FAS160COAr\|LmjF.14.0690 | UD | N |
| NPHPPH\|LmjF.31.2340 | UD | E |  | ADNCYC\|LmjF.05.1220 | UD | N |  | FAS180COAr\|LmjF.14.0705 | UD | N |
| NPHPPH\|LmjF.31.2340 | UD | E |  | ADNCYC\|LmjF.17.0237 | UD | N |  | FAS180COAr\|LmjF.14.0690 | UD | N |
| NTRLASE\|LmjF.26.2280 | UD | E |  | ADNCYC\|LmjF.17.0230 | UD | N |  | FAS180COAr\|LmjF.14.0680 | UD | N |
| NTRLASE\|LmjF.26.2280 | UD | E |  | ADNCYC\|LmjF.36.3180 | UD | N |  | FAS180COAr\|LmjF.14.0700 | UD | N |
| NTRLASE4\|LmjF.26.2280 | UD | E |  | ADNCYC\|LmjF.17.0200 | UD | N |  | FAS203COA\|LmjF.32.1160 | UD | N |
| NTRLASE4\|LmjF.26.2280 | UD | E |  | ADNK1c\|LmjF.34.3600 | UD | N |  | FAS204COA\|LmjF.32.1160 | UD | N |
| OCCOADm\|LmjF.28.2510 | UD | E |  | ADNK1c\|LmjF.30.0890 | UD | N |  | FAS224COA\|LmjF.05.1170 | UD | N |
| OCCOADm\|LmjF.06.0880 | UD | E |  | ADNK1c\|LmjF.30.0880 | UD | N |  | FAS224COAr\|LmjF.14.0710 | UD | N |
| OCDMAT8m\|LmjF.05.0520 | UD | E |  | ADNK1er\|LmjF.30.0890 | UD | N |  | FAS224COAr\|LmjF.14.0740 | UD | N |
| OCMAT3m\|LmjF.05.0520 | UD | E |  | ADNK1er\|LmjF.30.0880 | UD | N |  | FAS224COAr\|LmjF.14.0730 | UD | N |
| OCOAT1r_m\|LmjF.33.2340 | UD | E |  | ADNK1er\|LmjF.34.3600 | UD | N |  | FAS224COAr\|LmjF.14.0720 | UD | N |
| OCOAT1r_m\|LmjF.30.1930 | UD | E |  | ADNK1g\|LmjF.34.3600 | UD | N |  | FAS225COA\|LmjF.05.1170 | UD | N |
| OCOAT1r_m\|LmjF.30.1940 | UD | E |  | ADNK1g\|LmjF.30.0890 | UD | N |  | FAS60COAr\|LmjF.14.0660 | UD | N |
| ODH1mi\|LmjF.21.1430 | UD | E |  | ADNK1g\|LmjF.30.0880 | UD | N |  | FAS60COAr\|LmjF.14.0650 | UD | N |
| ODH2mi\|LmjF.21.1430 | UD | E |  | ADNK1m\|LmjF.30.0890 | UD | N |  | FAS60COAr\|LmjF.14.0640 | UD | N |
| ODHmi\|LmjF.21.1430 | UD | E |  | ADNK1m\|LmjF.30.0880 | UD | N |  | FAS80COAr\|LmjF.14.0650 | UD | N |
| OHPHM\|LmjF.35.4250 | UD | E |  | ADNK1m\|LmjF.34.3600 | UD | N |  | FAS80COAr\|LmjF.14.0660 | UD | N |
| OMPDCg\|LmjF.16.0550 | UD | E |  | ADPT2\|LmjF.26.0140 | UD | N |  | FAS80COAr\|LmjF.14.0640 | UD | N |
| ORNDC\|LmjF.12.0280 | UD | E |  | ADPTr\|LmjF.26.0140 | UD | N |  | FBAg\|LmjF.36.1260 | UD | N |
| ORPTg\|LmjF.16.0550 | UD | E |  | ADSL1r\|LmjF.04.0460 | UD | N |  | FBP26\|LmjF.36.0150 | UD | N |
| P5CDm_i\|LmjF.03.0200 | UD | E |  | ADSL2r\|LmjF.04.0460 | UD | N |  | FBP26\|LmjF.07.0760 | UD | N |
| P5CDr\|LmjF.03.0200 | UD | E |  | ADSS_i\|LmjF.13.1190 | UD | N |  | FCLT\|LmjF.17.1460 | UD | N |
| P5CRr\|LmjF.13.1680 | UD | E |  | AGDC\|LmjF.36.0040 | UD | N |  | FERCOAL\|LmjF.19.0985 | UD | N |
| P5CRrm\|LmjF.13.1680 | UD | E |  | AGMT\|LmjF.23.0070 | UD | N |  | FERCOAL\|LmjF.19.1005 | UD | N |
| PAPA_LM\|LmjF.19.1350 | UD | E |  | AGPATi_LM\|LmjF.32.1960 | UD | N |  | FRDg\|LmjF.35.1180 | UD | N |
| PAPA_LM\|LmjF.18.0440 | UD | E |  | AGPATim_LM\|LmjF.32.1960 | UD | N |  | FRDm\|LmjF.35.1190 | UD | N |
| PAPAm_LM\|LmjF.19.1350 | UD | E |  | AHC\|LmjF.36.3910 | UD | N |  | FRDm\|LmjF.35.0830 | UD | N |
| PAPAm_LM\|LmjF.18.0440 | UD | E |  | AHSERL2\|LmjF.35.3230 | UD | N |  | FTHFLr\|LmjF.30.2600 | UD | N |
| PDHe1\|LmjF.18.1380 | UD | E |  | AHSERL5i\|LmjF.36.3590 | UD | N |  | FUMg\|LmjF.29.1960 | UD | N |
| PDHe1\|LmjF.35.0050 | UD | E |  | AKAGPP_LM\|LmjF.19.1350 | UD | N |  | FUMm\|LmjF.24.0320 | UD | N |
| PDHe1\|LmjF.25.1710 | UD | E |  | AKAGPP_LM\|LmjF.18.0440 | UD | N |  | G3PDcm\|LmjF.28.0240 | UD | N |
| PDHe2\|LmjF.36.2660 | UD | E |  | AKGDE1B\|LmjF.36.3470 | UD | N |  | G3PDcm\|LmjF.20.0430 | UD | N |
| PDHe2\|LmjF.21.0550 | UD | E |  | AKGDE1B\|LmjF.27.0880 | UD | N |  | G3PDg\|LmjF.10.0510 | UD | N |
| PDHe3\|LmjF.31.2650 | UD | E |  | AKGDE2B\|LmjF.28.2420 | UD | N |  | G5SDr\|LmjF.32.3140 | UD | N |
| PDHe3\|LmjF.32.3310 | UD | E |  | AKGDe1\|LmjF.36.3470 | UD | N |  | G6PDAr\|LmjF.32.3260 | UD | N |
| PDHe3\|LmjF.29.1830 | UD | E |  | AKGDe1\|LmjF.27.0880 | UD | N |  | G6PDH1\|LmjF.34.0080 | UD | N |
| PDHe3\|LmjF.31.2640 | UD | E |  | AKGDe2\|LmjF.28.2420 | UD | N |  | G6PDHg\|LmjF.34.0080 | UD | N |
| PDHe3\|LmjF.31.2640 | UD | E |  | ALAR\|LmjF.23.1480 | UD | N |  | GALKx\|LmjF.35.2740 | UD | N |
| PETOHM_LM\|LmjF.31.2290 | UD | E |  | ALATA_L\|LmjF.12.0630 | UD | N |  | GALS3a\|LmjF.18.0090 | UD | N |
| PETOHMm_LM\|LmjF.31.2290 | UD | E |  | ALATA_Lm\|LmjF.12.0630 | UD | N |  | GAPD\|LmjF.35.4750 | UD | N |
| PFK26\|LmjF.03.0800 | UD | E |  | ALATRSi\|LmjF.22.1540 | UD | N |  | GAPDg\|LmjF.36.2350 | UD | N |
| PFK26\|LmjF.26.0310 | UD | E |  | ALA_Dt6\|LmjF.22.0230 | UD | N |  | GAPDg\|LmjF.30.2980 | UD | N |
| PFKg\|LmjF.29.2510 | UD | E |  | ALA_Dt6\|LmjF.14.0320 | UD | N |  | GAPDg\|LmjF.30.2970 | UD | N |
| PGCDr\|LmjF.03.0030 | UD | E |  | ALA_Dt6\|LmjF.27.0670 | UD | N |  | GCCa\|LmjF.26.0030 | UD | N |
| PGDH\|LmjF.35.3340 | UD | E |  | ALA_Dt6\|LmjF.11.0520 | UD | N |  | GCCb\|LmjF.36.3800 | UD | N |
| PGI1\|LmjF.12.0530 | UD | E |  | ALA_Lt6\|LmjF.11.0520 | UD | N |  | GCCb\|LmjF.36.3810 | UD | N |
| PGI2\|LmjF.12.0530 | UD | E |  | ALA_Lt6\|LmjF.22.0230 | UD | N |  | GCCbm\|LmjF.36.3800 | UD | N |
| PGI3\|LmjF.12.0530 | UD | E |  | ALA_Lt6\|LmjF.14.0320 | UD | N |  | GCCbm\|LmjF.36.3810 | UD | N |
| PGK\|LmjF.20.0110 | UD | E |  | ALA_Lt6\|LmjF.27.0670 | UD | N |  | GHMT\|LmjF.14.1320 | UD | N |
| PGK\|LmjF.20.0110 | UD | E |  | ALCD19\|LmjF.23.0360 | UD | N |  | GHMT\|LmjF.28.2370 | UD | N |
| PGK\|LmjF.20.0100 | UD | E |  | ALCD19\|LmjF.30.2090 | UD | N |  | GK1\|LmjF.36.2260 | UD | N |
| PGK\|LmjF.30.3380 | UD | E |  | ALCD3\|LmjF.23.0360 | UD | N |  | GK1\|LmjF.33.1090 | UD | N |
| PGKg\|LmjF.20.0100 | UD | E |  | ALDD19m\|LmjF.25.1120 | UD | N |  | GK1er\|LmjF.33.1090 | UD | N |
| PGKg\|LmjF.30.3380 | UD | E |  | ALDD20m\|LmjF.25.1120 | UD | N |  | GK1er\|LmjF.36.2260 | UD | N |
| PGKg\|LmjF.20.0110 | UD | E |  | ALDD2xm\|LmjF.25.1120 | UD | N |  | GLCAt2\|LmjF.33.0290 | UD | N |
| PGKg\|LmjF.20.0110 | UD | E |  | ALDD32m\|LmjF.25.1120 | UD | N |  | GLCAt2\|LmjF.36.6280 | UD | N |
| PGL\|LmjF.26.2700 | UD | E |  | ALDD4xm\|LmjF.25.1120 | UD | N |  | GLCAt2\|LmjF.36.6290 | UD | N |
| PGLg\|LmjF.26.2700 | UD | E |  | ALDD6xm\|LmjF.25.1120 | UD | N |  | GLCAt2\|LmjF.36.6300 | UD | N |
| PGM\|LmjF.33.2110 | UD | E |  | ALDD8xr_m\|LmjF.25.1120 | UD | N |  | GLCBt2\|LmjF.36.6300 | UD | N |
| PGM\|LmjF.36.4070 | UD | E |  | ALDOXm\|LmjF.25.1120 | UD | N |  | GLCBt2\|LmjF.36.6290 | UD | N |
| PGM\|LmjF.28.2220 | UD | E |  | ALDR\|LmjF.31.2880 | UD | N |  | GLCBt2\|LmjF.36.6280 | UD | N |
| PGM\|LmjF.36.6650 | UD | E |  | AMPDg\|LmjF.13.0980 | UD | N |  | GLCBt2\|LmjF.33.0290 | UD | N |
| PGM\|LmjF.08.0060 | UD | E |  | AMPDg\|LmjF.32.2550 | UD | N |  | GLNS_i\|LmjF.06.0370 | UD | N |
| PGMT\|LmjF.21.0640 | UD | E |  | AMPDg\|LmjF.04.0280 | UD | N |  | GLNTRS\|LmjF.15.1440 | UD | N |
| PHCYT_LM\|LmjF.26.1620 | UD | E |  | AMPDg\|LmjF.35.4800 | UD | N |  | GLNTRSm_i\|LmjF.15.1440 | UD | N |
| PHCYTm_LM\|LmjF.26.1620 | UD | E |  | APIND\|LmjF.27.0090 | UD | N |  | GLNt\|LmjF.27.0670 | UD | N |
| PHETA1\|LmjF.35.0820 | UD | E |  | APINM\|LmjF.27.0090 | UD | N |  | GLNt\|LmjF.14.0320 | UD | N |
| PHETA1\|LmjF.36.2360 | UD | E |  | APase\|LmjF.25.1960 | UD | N |  | GLNt\|LmjF.22.0230 | UD | N |
| PHETA1m\|LmjF.24.0370 | UD | E |  | ARGNg\|LmjF.35.1480 | UD | N |  | GLNt\|LmjF.11.0520 | UD | N |
| PHETRS\|LmjF.32.0870 | UD | E |  | ARGSSr\|LmjF.23.0260 | UD | N |  | GLU5K\|LmjF.26.2710 | UD | N |
| PHETRS\|LmjF.19.1040 | UD | E |  | ARGTRS\|LmjF.27.1310 | UD | N |  | GLUCYSL\|LmjF.18.1660 | UD | N |
| PHEt6\|LmjF.22.0230 | UD | E |  | ARGt\|LmjF.14.0320 | UD | N |  | GLUDx [m]\|LmjF.15.1010 | UD | N |
| PHEt6\|LmjF.14.0320 | UD | E |  | ARGt\|LmjF.22.0230 | UD | N |  | GLUDy\|LmjF.28.2910 | UD | N |
| PHEt6\|LmjF.27.0670 | UD | E |  | ARGt\|LmjF.27.0670 | UD | N |  | GLUKg\|LmjF.36.2320 | UD | N |
| PHEt6\|LmjF.11.0520 | UD | E |  | ARGt\|LmjF.11.0520 | UD | N |  | GLUKg\|LmjF.21.0250 | UD | N |
| PI45BPP_LM\|LmjF.35.0040 | UD | E |  | ARMT\|LmjF.12.1270 | UD | N |  | GLUKg\|LmjF.21.0240 | UD | N |
| PI45BPP_LM\|LmjF.30.2950 | UD | E |  | ARMT\|LmjF.03.0600 | UD | N |  | GLUTRS\|LmjF.30.3240 | UD | N |
| PI4P5K_LM\|LmjF.34.3090 | UD | E |  | ASAT\|LmjF.31.1780 | UD | N |  | GLUTRSm\|LmjF.30.3240 | UD | N |
| PIN3K_LM\|LmjF.24.2010 | UD | E |  | ASCBPm\|LmjF.34.0070 | UD | N |  | GLUt6\|LmjF.11.0520 | UD | N |
| PIN3K_LM\|LmjF.02.0120 | UD | E |  | ASNN\|LmjF.15.0390 | UD | N |  | GLUt6\|LmjF.27.0670 | UD | N |
| PIN3K_LM\|LmjF.20.1120 | UD | E |  | ASNNg\|LmjF.36.4430 | UD | N |  | GLUt6\|LmjF.14.0320 | UD | N |
| PIN3K_LM\|LmjF.30.1850 | UD | E |  | ASNS3\|LmjF.26.0830 | UD | N |  | GLUt6\|LmjF.22.0230 | UD | N |
| PIN3K_LM\|LmjF.34.3940 | UD | E |  | ASNTRS\|LmjF.34.2340 | UD | N |  | GLYCKr\|LmjF.02.0500 | UD | N |
| PIN4K_LM\|LmjF.34.3590 | UD | E |  | ASNTRS\|LmjF.34.2340 | UD | N |  | GLYKg\|LmjF.35.3080 | UD | N |
| PINOS_LM\|LmjF.26.2480 | UD | E |  | ASNt6\|LmjF.11.0520 | UD | N |  | GLYOX1\|LmjF.12.0220 | UD | N |
| PIt6\|LmjF.03.0500 | UD | E |  | ASNt6\|LmjF.14.0320 | UD | N |  | GLYPATm\|LmjF.25.0020 | UD | N |
| PIt6\|LmjF.10.0030 | UD | E |  | ASNt6\|LmjF.22.0230 | UD | N |  | GLYPATm\|LmjF.05.0180 | UD | N |
| PIt6\|LmjF.10.1300 | UD | E |  | ASNt6\|LmjF.27.0670 | UD | N |  | GLYPHATm\|LmjF.05.0180 | UD | N |
| PItm\|LmjF.05.0290 | UD | E |  | ASPCT\|LmjF.16.0540 | UD | N |  | GLYPHATm\|LmjF.25.0020 | UD | N |
| PItm\|LmjF.35.4420 | UD | E |  | ASPTA1\|LmjF.35.0820 | UD | N |  | GLYTRS\|LmjF.36.3840 | UD | N |
| PItm\|LmjF.35.4430 | UD | E |  | ASPTA1m\|LmjF.24.0370 | UD | N |  | GLYt6\|LmjF.14.0320 | UD | N |
| PLAc_LM\|LmjF.35.3020 | UD | E |  | ASPTA3\|LmjF.35.0820 | UD | N |  | GLYt6\|LmjF.22.0230 | UD | N |
| PLAe_LM\|LmjF.35.3020 | UD | E |  | ASPTA4\|LmjF.35.0820 | UD | N |  | GLYt6\|LmjF.27.0670 | UD | N |
| PMANM\|LmjF.36.1960 | UD | E |  | ASPTA5m\|LmjF.24.0370 | UD | N |  | GLYt6\|LmjF.11.0520 | UD | N |
| PMANMg\|LmjF.34.3780 | UD | E |  | ASPTRS\|LmjF.21.0895 | UD | N |  | GMPS2\|LmjF.22.0110 | UD | N |
| PMETM_LM\|LmjF.31.3120 | UD | E |  | ASPTRS\|LmjF.30.0460 | UD | N |  | GPAM_LM\|LmjF.34.1090 | UD | N |
| PMETMm_LM\|LmjF.31.3120 | UD | E |  | ASPt6\|LmjF.14.0320 | UD | N |  | GPAMm_LM\|LmjF.34.1090 | UD | N |
| PMEVK\|LmjF.15.1460 | UD | E |  | ASPt6\|LmjF.22.0230 | UD | N |  | GPDDA1\|LmjF.28.2100 | UD | N |
| PMEVKx\|LmjF.15.1460 | UD | E |  | ASPt6\|LmjF.27.0670 | UD | N |  | GPDDA1\|LmjF.36.5960 | UD | N |
| PNS1\|LmjF.29.2800 | UD | E |  | ASPt6\|LmjF.11.0520 | UD | N |  | GPDDA2\|LmjF.36.5960 | UD | N |
| PNS2\|LmjF.29.2800 | UD | E |  | ATPM\|LmjF.36.4630 | UD | N |  | GPDDA2\|LmjF.28.2100 | UD | N |
| PNS3\|LmjF.29.2800 | UD | E |  | ATPS\|LmjF.18.1510 | UD | N |  | GRTT\|LmjF.22.1360 | UD | N |
| PNS4\|LmjF.29.2800 | UD | E |  | ATPS\|LmjF.18.1520 | UD | N |  | GRTTx\|LmjF.22.1360 | UD | N |
| PNTK\|LmjF.28.0140 | UD | E |  | ATPS3v\|LmjF.18.0560 | UD | N |  | GSADHm\|LmjF.03.0200 | UD | N |
| PNTK2\|LmjF.28.0140 | UD | E |  | ATPS3v\|LmjF.23.0340 | UD | N |  | GTHP\|LmjF.26.0810 | UD | N |
| PPA\|LmjF.03.0910 | UD | E |  | ATPS3v\|LmjF.30.3660 | UD | N |  | GTHP\|LmjF.26.0820 | UD | N |
| PPA_1\|LmjF.31.1220 | UD | E |  | ATPS3v\|LmjF.35.0700 | UD | N |  | GTHP\|LmjF.36.3010 | UD | N |
| PPA_1v\|LmjF.11.0210 | UD | E |  | ATPS3v\|LmjF.21.1800 | UD | N |  | GTHP\|LmjF.26.0800 | UD | N |
| PPCDC\|LmjF.30.1540 | UD | E |  | ATPS3v\|LmjF.21.1790 | UD | N |  | GTHPm\|LmjF.26.0820 | UD | N |
| PPCKg\|LmjF.27.1805 | UD | E |  | ATPS3v\|LmjF.21.1340 | UD | N |  | GTHPm\|LmjF.26.0810 | UD | N |
| PPCKg\|LmjF.27.1810 | UD | E |  | ATPS3v\|LmjF.23.1510 | UD | N |  | GTHPm\|LmjF.26.0800 | UD | N |
| PPCOACm\|LmjF.28.0490 | UD | E |  | ATPS3v\|LmjF.28.2430 | UD | N |  | GTHPm\|LmjF.36.3010 | UD | N |
| PPCOACm\|LmjF.01.0050 | UD | E |  | ATPS3v\|LmjF.34.3670 | UD | N |  | GTHPx\|LmjF.36.3010 | UD | N |
| PPDKg\|LmjF.11.1000 | UD | E |  | ATPS3v\|LmjF.05.1140 | UD | N |  | GTHPx\|LmjF.26.0800 | UD | N |
| PPPGO\|LmjF.06.1280 | UD | E |  | ATPS3v\|LmjF.36.3100 | UD | N |  | GTHPx\|LmjF.26.0820 | UD | N |
| PROTRS\|LmjF.18.1210 | UD | E |  | ATPS3v\|LmjF.12.0520 | UD | N |  | GTHPx\|LmjF.26.0810 | UD | N |
| PROt6\|LmjF.27.0670 | UD | E |  | ATPS3v\|LmjF.28.1160 | UD | N |  | GTHS\|LmjF.14.0910 | UD | N |
| PROt6\|LmjF.22.0230 | UD | E |  | ATPS3v\|LmjF.23.0130 | UD | N |  | GUACYC\|LmjF.17.0190 | UD | N |
| PROt6\|LmjF.14.0320 | UD | E |  | ATPSm\|LmjF.30.3600 | UD | N |  | GUACYC\|LmjF.05.1220 | UD | N |
| PROt6\|LmjF.11.0520 | UD | E |  | ATPSm\|LmjF.25.1180 | UD | N |  | GUACYC\|LmjF.17.0235 | UD | N |
| PRPPSi\|LmjF.08.0510 | UD | E |  | ATPSm\|LmjF.25.1170 | UD | N |  | GUACYC\|LmjF.17.0230 | UD | N |
| PRPPSi\|LmjF.08.1130 | UD | E |  | ATPSm\|LmjF.21.1770 | UD | N |  | GUACYC\|LmjF.17.0237 | UD | N |
| PRPPSig\|LmjF.36.5390 | UD | E |  | ATPSm\|LmjF.05.0500 | UD | N |  | GUACYC\|LmjF.36.3180 | UD | N |
| PRPPSig\|LmjF.33.1930 | UD | E |  | ATPSm\|LmjF.21.0740 | UD | N |  | GUACYC\|LmjF.17.0200 | UD | N |
| PSD_LM\|LmjF.35.4590 | UD | E |  | ATPSm\|LmjF.24.0630 | UD | N |  | GUAD\|LmjF.29.0867 | UD | N |
| PSDm_LM\|LmjF.35.4590 | UD | E |  | ATPSm\|LmjF.05.0510 | UD | N |  | GUAPRTr\|LmjF.26.0140 | UD | N |
| PSUDS\|LmjF.26.0420 | UD | E |  | ATPSm\|LmjF.26.0460 | UD | N |  | H3MS\|LmjF.36.5360 | UD | N |
| PSUDS\|LmjF.36.1660 | UD | E |  | BNZPPH\|LmjF.25.1960 | UD | N |  | H3MS2m\|LmjF.29.2350 | UD | N |
| PSUDS\|LmjF.30.1550 | UD | E |  | BTCOADm\|LmjF.06.0880 | UD | N |  | HACD1m\|LmjF.36.1140 | UD | N |
| PTHK\|LmjF.28.0140 | UD | E |  | BTCOADm\|LmjF.28.2510 | UD | N |  | HACD1m\|LmjF.26.1550 | UD | N |
| PTROPACE\|LmjF.25.0020 | UD | E |  | BTMAT1m\|LmjF.05.0520 | UD | N |  | HACD2m\|LmjF.36.1140 | UD | N |
| PTROPACE\|LmjF.05.0180 | UD | E |  | BTNL1\|LmjF.31.1070 | UD | N |  | HACD2m\|LmjF.26.1550 | UD | N |
| PUNP8I\|LmjF.29.2800 | UD | E |  | C14STRr\|LmjF.32.2320 | UD | N |  | HACD3m\|LmjF.36.1140 | UD | N |
| PYDAMK\|LmjF.30.1250 | UD | E |  | C24STRer\|LmjF.33.0680 | UD | N |  | HACD3m\|LmjF.26.1550 | UD | N |
| PYDXK\|LmjF.30.1250 | UD | E |  | C3STDH1r\|LmjF.06.0350 | UD | N |  | HACD4m\|LmjF.36.1140 | UD | N |
| PYDXNK\|LmjF.30.1250 | UD | E |  | C3STDH2r\|LmjF.06.0350 | UD | N |  | HACD4m\|LmjF.26.1550 | UD | N |
| PYK\|LmjF.35.0020 | UD | E |  | C4STMO1r\|LmjF.36.2540 | UD | N |  | HACD5m\|LmjF.36.1140 | UD | N |
| PYK\|LmjF.35.0030 | UD | E |  | C4STMO2r\|LmjF.36.2540 | UD | N |  | HACD5m\|LmjF.26.1550 | UD | N |
| PYRZAMn\|LmjF.26.0210 | UD | E |  | C5STDSr\|LmjF.31.0590 | UD | N |  | HACD6m\|LmjF.26.1550 | UD | N |
| PYRZAMn\|LmjF.34.2140 | UD | E |  | C8STIr\|LmjF.29.2140 | UD | N |  | HACD6m\|LmjF.36.1140 | UD | N |
| RAFFH\|LmjF.23.0870 | UD | E |  | CA2abc\|LmjF.04.0010 | UD | N |  | HACD7m\|LmjF.26.1550 | UD | N |
| RAFFH\|LmjF.27.2340 | UD | E |  | CDAL\|LmjF.16.0590 | UD | N |  | HACD7m\|LmjF.36.1140 | UD | N |
| RAFFH\|LmjF.23.0880 | UD | E |  | CDGPTm_LM\|LmjF.07.0200 | UD | N |  | HACD8m\|LmjF.36.1140 | UD | N |
| RAFFH\|LmjF.04.0310 | UD | E |  | CDPDSP_LM\|LmjF.14.1200 | UD | N |  | HACD8m\|LmjF.26.1550 | UD | N |
| RBK_Dg\|LmjF.36.0060 | UD | E |  | CDPDSPm_LM\|LmjF.14.1200 | UD | N |  | HACD9g\|LmjF.24.2030 | UD | N |
| RBLKg\|LmjF.36.0060 | UD | E |  | CHLPCTD\|LmjF.18.1330 | UD | N |  | HACD9g\|LmjF.33.2600 | UD | N |
| RNDR1(n)\|LmjF.28.0890 | UD | E |  | CHLSTIr\|LmjF.16.1340 | UD | N |  | HACD9m\|LmjF.26.1550 | UD | N |
| RNDR1(n)\|LmjF.27.2050 | UD | E |  | CHOLK\|LmjF.35.1470 | UD | N |  | HACD9m\|LmjF.36.1140 | UD | N |
| RNDR1(n)\|LmjF.22.1290 | UD | E |  | CLPNSm_LM\|LmjF.34.2110 | UD | N |  | HACOADm\|LmjF.26.1550 | UD | N |
| RNDR2(n)\|LmjF.28.0890 | UD | E |  | CPPPGO\|LmjF.06.1270 | UD | N |  | HACOADm\|LmjF.36.1140 | UD | N |
| RNDR2(n)\|LmjF.27.2050 | UD | E |  | CSm\|LmjF.18.0680 | UD | N |  | HBCOADm\|LmjF.36.1140 | UD | N |
| RNDR2(n)\|LmjF.22.1290 | UD | E |  | CSm\|LmjF.18.0670 | UD | N |  | HBCOAE\|LmjF.26.1550 | UD | N |
| RNDR3(n)\|LmjF.28.0890 | UD | E |  | CTL2\|LmjF.14.0460 | UD | N |  | HBUR1m\|LmjF.24.2030 | UD | N |
| RNDR3(n)\|LmjF.27.2050 | UD | E |  | CTL2\|LmjF.32.2640 | UD | N |  | HBUR1m\|LmjF.27.2440 | UD | N |
| RNDR3(n)\|LmjF.22.1290 | UD | E |  | CTL2\|LmjF.32.2640 | UD | N |  | HCYSMT\|LmjF.36.6310 | UD | N |
| RNDR4(n)\|LmjF.22.1290 | UD | E |  | CTPS1n\|LmjF.20.0560 | UD | N |  | HDCOADm\|LmjF.06.0880 | UD | N |
| RNDR4(n)\|LmjF.28.0890 | UD | E |  | CTPS2n\|LmjF.20.0560 | UD | N |  | HDCOADm\|LmjF.28.2510 | UD | N |
| RNDR4(n)\|LmjF.27.2050 | UD | E |  | CYOO6m\|LmjF.28.2680 | UD | N |  | HDDR5m\|LmjF.24.2030 | UD | N |
| RPE\|LmjF.33.1570 | UD | E |  | CYOO6m\|LmjF.31.1570 | UD | N |  | HDDR5m\|LmjF.27.2440 | UD | N |
| RPEg\|LmjF.35.3680 | UD | E |  | CYOO6m\|LmjF.12.0670 | UD | N |  | HDER4m\|LmjF.24.2030 | UD | N |
| RPI\|LmjF.28.1970 | UD | E |  | CYOO6m\|LmjF.04.1130 | UD | N |  | HDER4m\|LmjF.27.2440 | UD | N |
| S6PFH\|LmjF.23.0870 | UD | E |  | CYOO6m\|LmjF.03.0100 | UD | N |  | HDMAT7m\|LmjF.05.0520 | UD | N |
| S6PFH\|LmjF.27.2340 | UD | E |  | CYOO6m\|LmjF.26.1710 | UD | N |  | HEMAT2m\|LmjF.05.0520 | UD | N |
| S6PFH\|LmjF.23.0880 | UD | E |  | CYOO6m\|LmjF.25.1130 | UD | N |  | HEMEASm\|LmjF.28.2680 | UD | N |
| S6PFH\|LmjF.04.0310 | UD | E |  | CYOO6m\|LmjF.36.6995 | UD | N |  | HEMEOSm\|LmjF.23.1520 | UD | N |
| SAM24MTr\|LmjF.36.2380 | UD | E |  | CYOO6m\|LmjF.20.0840 | UD | N |  | HEX4g\|LmjF.21.0250 | UD | N |
| SBPP1r\|LmjF.32.2290 | UD | E |  | CYOO6m\|LmjF.03.0740 | UD | N |  | HEX4g\|LmjF.21.0240 | UD | N |
| SBPP3\|LmjF.19.1350 | UD | E |  | CYOO6m\|LmjF.23.0370 | UD | N |  | HEX7g\|LmjF.21.0240 | UD | N |
| SBPP3\|LmjF.18.0440 | UD | E |  | CYOO6m\|LmjF.21.1710 | UD | N |  | HEX7g\|LmjF.21.0250 | UD | N |
| SBTD_D\|LmjF.33.0520 | UD | E |  | CYOR_u6m\|LmjF.35.1380 | UD | N |  | HEXg\|LmjF.36.2320 | UD | N |
| SERAT\|LmjF.34.2850 | UD | E |  | CYOR_u6m\|LmjF.07.0060 | UD | N |  | HEXg\|LmjF.21.0250 | UD | N |
| SERTRS\|LmjF.11.0100 | UD | E |  | CYOR_u6m\|LmjF.35.1540 | UD | N |  | HEXg\|LmjF.21.0240 | UD | N |
| SERt6\|LmjF.11.0520 | UD | E |  | CYOR_u6m\|LmjF.31.2580 | UD | N |  | HHDR7m\|LmjF.24.2030 | UD | N |
| SERt6\|LmjF.22.0230 | UD | E |  | CYSBS\|LmjF.17.0250 | UD | N |  | HHDR7m\|LmjF.27.2440 | UD | N |
| SERt6\|LmjF.14.0320 | UD | E |  | CYSS\|LmjF.36.3590 | UD | N |  | HHYR2m\|LmjF.27.2440 | UD | N |
| SERt6\|LmjF.27.0670 | UD | E |  | CYSTL\|LmjF.32.2640 | UD | N |  | HHYR2m\|LmjF.24.2030 | UD | N |
| SGPL12r\|LmjF.30.2350 | UD | E |  | CYSTL\|LmjF.32.2640 | UD | N |  | HIACL\|LmjF.29.0600 | UD | N |
| SHSL1\|LmjF.35.3230 | UD | E |  | CYSTL\|LmjF.14.0460 | UD | N |  | HIBHrm\|LmjF.32.3670 | UD | N |
| SHSL2r\|LmjF.35.3230 | UD | E |  | CYSTRS\|LmjF.12.0250 | UD | N |  | HIBHrm\|LmjF.32.3660 | UD | N |
| SHSL4r\|LmjF.35.3230 | UD | E |  | CYSTS\|LmjF.17.0250 | UD | N |  | HIBHrm\|LmjF.32.3650 | UD | N |
| SINCOAL\|LmjF.19.1005 | UD | E |  | CYSt6\|LmjF.11.0520 | UD | N |  | HISTRS\|LmjF.30.0630 | UD | N |
| SINCOAL\|LmjF.19.0985 | UD | E |  | CYSt6\|LmjF.22.0230 | UD | N |  | HISt6\|LmjF.11.0520 | UD | N |
| SLCBK1\|LmjF.18.0440 | UD | E |  | CYSt6\|LmjF.14.0320 | UD | N |  | HISt6\|LmjF.14.0320 | UD | N |
| SLCBK1\|LmjF.19.1350 | UD | E |  | CYSt6\|LmjF.27.0670 | UD | N |  | HISt6\|LmjF.22.0230 | UD | N |
| SLCYSS\|LmjF.36.3590 | UD | E |  | CYTD1\|LmjF.17.0360 | UD | N |  | HISt6\|LmjF.27.0670 | UD | N |
| SO4t6\|LmjF.28.1690 | UD | E |  | CYTDK1\|LmjF.31.2470 | UD | N |  | HKAm\|LmjF.25.2010 | UD | N |
| SPHK21r\|LmjF.32.2290 | UD | E |  | CYTDK2\|LmjF.31.2470 | UD | N |  | HMGCOARi\|LmjF.30.3190 | UD | N |
| SPHPLr\|LmjF.30.2350 | UD | E |  | CYTDK3\|LmjF.31.2470 | UD | N |  | HMGCOARi_x\|LmjF.30.3190 | UD | N |
| SPMS\|LmjF.04.0580 | UD | E |  | CYTDK4\|LmjF.31.2470 | UD | N |  | HOCR3m\|LmjF.27.2440 | UD | N |
| SQLMer\|LmjF.13.1620 | UD | E |  | CYTDK5\|LmjF.31.2470 | UD | N |  | HOCR3m\|LmjF.24.2030 | UD | N |
| SQLS\|LmjF.31.2940 | UD | E |  | CYTDK6\|LmjF.31.2470 | UD | N |  | HODR8m\|LmjF.27.2440 | UD | N |
| SQLSg\|LmjF.31.2940 | UD | E |  | CYTDK7\|LmjF.31.2470 | UD | N |  | HODR8m\|LmjF.24.2030 | UD | N |
| SRTMT\|LmjF.12.1270 | UD | E |  | CYTDK8\|LmjF.31.2470 | UD | N |  | HOXPRx\|LmjF.30.0180 | UD | N |
| SRTMT\|LmjF.23.1200 | UD | E |  | CYTDK9\|LmjF.31.2470 | UD | N |  | HP5CDm\|LmjF.03.0200 | UD | N |
| SSALym\|LmjF.36.1760 | UD | E |  | CaATPAC\|LmjF.07.0630 | UD | N |  | HPDOAi\|LmjF.34.4330 | UD | N |
| STFH\|LmjF.04.0310 | UD | E |  | CaATPAC\|LmjF.07.0650 | UD | N |  | HPDOCi\|LmjF.34.4330 | UD | N |
| STFH\|LmjF.23.0870 | UD | E |  | DADAr\|LmjF.35.2160 | UD | N |  | HPROa\|LmjF.13.1680 | UD | N |
| STFH\|LmjF.23.0880 | UD | E |  | DADAr\|LmjF.33.0235 | UD | N |  | HPROam\|LmjF.13.1680 | UD | N |
| STFH\|LmjF.27.2340 | UD | E |  | DADK\|LmjF.34.0110 | UD | N |  | HPROxm\|LmjF.03.0200 | UD | N |
| SUCD1rm\|LmjF.24.1630 | UD | E |  | DADK\|LmjF.04.0960 | UD | N |  | HPROym\|LmjF.03.0200 | UD | N |
| SUCD2_u6m\|LmjF.15.0990 | UD | E |  | DADK\|LmjF.34.0120 | UD | N |  | HSD\|LmjF.07.0260 | UD | N |
| SUCOGDPm\|LmjF.36.2950 | UD | E |  | DADKf\|LmjF.21.1250 | UD | N |  | HSK\|LmjF.30.3080 | UD | N |
| SUCOGDPm\|LmjF.25.2140 | UD | E |  | DADKf\|LmjF.16.0020 | UD | N |  | HTDR6m\|LmjF.24.2030 | UD | N |
| SUCOGDPm\|LmjF.25.2130 | UD | E |  | DADKg\|LmjF.36.1360 | UD | N |  | HTDR6m\|LmjF.27.2440 | UD | N |
| SUCR1\|LmjF.23.0880 | UD | E |  | DADKg\|LmjF.25.2370 | UD | N |  | HXCOADm\|LmjF.06.0880 | UD | N |
| SUCR1\|LmjF.23.0870 | UD | E |  | DAGAT_LM\|LmjF.09.1040 | UD | N |  | HXCOADm\|LmjF.28.2510 | UD | N |
| SUCR1\|LmjF.27.2340 | UD | E |  | DAGCPT_LM\|LmjF.36.5900 | UD | N |  | HXPRTg\|LmjF.21.0845 | UD | N |
| SUCR1\|LmjF.04.0310 | UD | E |  | DAGCPTm_LM\|LmjF.36.5900 | UD | N |  | I14BP1P\|LmjF.31.2920 | UD | N |
| TA6PK\|LmjF.02.0030 | UD | E |  | DAGK_LM\|LmjF.16.1290 | UD | N |  | ICDHym\|LmjF.33.2550 | UD | N |
| TA6PK\|LmjF.25.2440 | UD | E |  | DAGK_LM\|LmjF.35.5370 | UD | N |  | ICDHym\|LmjF.10.0290 | UD | N |
| TAL\|LmjF.16.0760 | UD | E |  | DAGKm_LM\|LmjF.16.1290 | UD | N |  | ILETAm\|LmjF.27.2030 | UD | N |
| TCAFCOAL\|LmjF.19.0985 | UD | E |  | DAGKm_LM\|LmjF.35.5370 | UD | N |  | ILETRS\|LmjF.18.1220 | UD | N |
| TCAFCOAL\|LmjF.19.1005 | UD | E |  | DCCOADm\|LmjF.28.2510 | UD | N |  | ILETRS\|LmjF.36.5620 | UD | N |
| TCINCOAL\|LmjF.19.1005 | UD | E |  | DCCOADm\|LmjF.06.0880 | UD | N |  | ILETRS\|LmjF.36.5620 | UD | N |
| TCINCOAL\|LmjF.19.0985 | UD | E |  | DCYTD1\|LmjF.17.0360 | UD | N |  | ILEt6\|LmjF.35.4410 | UD | N |
| TDCOADm\|LmjF.06.0880 | UD | E |  | DDCOADm\|LmjF.06.0880 | UD | N |  | IMPDg\|LmjF.17.0725 | UD | N |
| TDCOADm\|LmjF.28.2510 | UD | E |  | DDCOADm\|LmjF.28.2510 | UD | N |  | IMPDg\|LmjF.19.1560 | UD | N |
| TDPGDH\|LmjF.26.2230 | UD | E |  | DDMAT5m\|LmjF.05.0520 | UD | N |  | INDPYRD\|LmjF.34.3250 | UD | N |
| THDSTIL\|LmjF.27.0090 | UD | E |  | DEMAT4m\|LmjF.05.0520 | UD | N |  | IPCS_LM\|LmjF.35.4990 | UD | N |
| THFAT\|LmjF.36.3810 | UD | E |  | DESAT180_9\|LmjF.14.0510 | UD | N |  | IPDDI\|LmjF.35.5330 | UD | N |
| THFAT\|LmjF.36.3800 | UD | E |  | DESAT180_9\|LmjF.24.2250 | UD | N |  | IPDDIg\|LmjF.35.5330 | UD | N |
| THFATm\|LmjF.36.3810 | UD | E |  | DESAT181_12\|LmjF.33.3270 | UD | N |  | IPDPUPT\|LmjF.13.0020 | UD | N |
| THFATm\|LmjF.36.3800 | UD | E |  | DESAT182_15\|LmjF.10.0010 | UD | N |  | IPP5P\|LmjF.11.1010 | UD | N |
| THFGLUS\|LmjF.36.2610 | UD | E |  | DESAT182_6\|LmjF.36.6950 | UD | N |  | IPP5P2\|LmjF.11.1010 | UD | N |
| THRA\|LmjF.01.0480 | UD | E |  | DESAT183_6\|LmjF.36.6950 | UD | N |  | KEPTATm\|LmjF.25.0020 | UD | N |
| THRLAD\|LmjF.01.0480 | UD | E |  | DESAT203_5\|LmjF.07.1090 | UD | N |  | KEPTATm\|LmjF.05.0180 | UD | N |
| THRS\|LmjF.14.0350 | UD | E |  | DESAT204_5\|LmjF.07.1090 | UD | N |  | KYNASE1\|LmjF.26.2240 | UD | N |
| THRTRS\|LmjF.35.1410 | UD | E |  | DESAT224_4\|LmjF.14.1340 | UD | N |  | KYNASE2\|LmjF.26.2240 | UD | N |
| THRt6\|LmjF.11.0520 | UD | E |  | DESAT225_4\|LmjF.14.1340 | UD | N |  | KYNASE3\|LmjF.26.2240 | UD | N |
| THRt6\|LmjF.27.0670 | UD | E |  | DGK1\|LmjF.36.2260 | UD | N |  | LCAD\|LmjF.36.5180 | UD | N |
| THRt6\|LmjF.22.0230 | UD | E |  | DGK1\|LmjF.33.1090 | UD | N |  | LCAD\|LmjF.30.2900 | UD | N |
| THRt6\|LmjF.14.0320 | UD | E |  | DGK1er\|LmjF.33.1090 | UD | N |  | LDH_D\|LmjF.27.2020 | UD | N |
| TKT1\|LmjF.24.2060 | UD | E |  | DGK1er\|LmjF.36.2260 | UD | N |  | LDH_D\|LmjF.29.0280 | UD | N |
| TKT1g\|LmjF.24.2060 | UD | E |  | DHAK\|LmjF.10.0420 | UD | N |  | LDH_Dg\|LmjF.29.0280 | UD | N |
| TKT2\|LmjF.24.2060 | UD | E |  | DHCRD1\|LmjF.26.1700 | UD | N |  | LDH_Dg\|LmjF.27.2020 | UD | N |
| TKT2g\|LmjF.24.2060 | UD | E |  | DHCRD1\|LmjF.26.1690 | UD | N |  | LEUTAm\|LmjF.27.2030 | UD | N |
| TMDK1m\|LmjF.21.1210 | UD | E |  | DHCRD1\|LmjF.26.1670 | UD | N |  | LEUTRS\|LmjF.13.1100 | UD | N |
| TNMT\|LmjF.12.1270 | UD | E |  | DHCRD1\|LmjF.26.1680 | UD | N |  | LEUTRS\|LmjF.13.1100 | UD | N |
| TNMT\|LmjF.23.1200 | UD | E |  | DHCRD2\|LmjF.26.1690 | UD | N |  | LEUt6\|LmjF.35.4410 | UD | N |
| TPIg\|LmjF.24.0850 | UD | E |  | DHCRD2\|LmjF.26.1700 | UD | N |  | LGTHL1\|LmjF.35.3010 | UD | N |
| TROPACE\|LmjF.05.0180 | UD | E |  | DHCRD2\|LmjF.26.1670 | UD | N |  | LGTHL1\|LmjF.35.3010 | UD | N |
| TROPACE\|LmjF.25.0020 | UD | E |  | DHCRD2\|LmjF.26.1680 | UD | N |  | LGTHL1m\|LmjF.35.3010 | UD | N |
| TRPTRS\|LmjF.29.0060 | UD | E |  | DHORD1\|LmjF.16.0530 | UD | N |  | LGTHL1m\|LmjF.35.3010 | UD | N |
| TRPTRS\|LmjF.23.0300 | UD | E |  | DHORTS\|LmjF.16.0580 | UD | N |  | LNSTLSr\|LmjF.06.0650 | UD | N |
| TRPabc\|LmjF.35.5350 | UD | E |  | DHPEG\|LmjF.30.2090 | UD | N |  | LPS2_LM\|LmjF.31.2460 | UD | N |
| TRPabc\|LmjF.36.6830 | UD | E |  | DKMD2K\|LmjF.34.4600 | UD | N |  | LPS2_LM\|LmjF.31.0840 | UD | N |
| TRPabc\|LmjF.31.1800 | UD | E |  | DKMD2K\|LmjF.20.0970 | UD | N |  | LPS2_LM\|LmjF.31.0830 | UD | N |
| TRPabc\|LmjF.27.1580 | UD | E |  | DM5ATm\|LmjF.25.0020 | UD | N |  | LPS_LM\|LmjF.31.2460 | UD | N |
| TRPabc\|LmjF.33.1420 | UD | E |  | DM5ATm\|LmjF.05.0180 | UD | N |  | LPS_LM\|LmjF.31.0840 | UD | N |
| TRPabc\|LmjF.36.0420 | UD | E |  | DMATT\|LmjF.22.1360 | UD | N |  | LPS_LM\|LmjF.31.0830 | UD | N |
| TRPabc\|LmjF.31.1820 | UD | E |  | DMATTx\|LmjF.22.1360 | UD | N |  | LSTO1r\|LmjF.23.1300 | UD | N |
| TRPabc\|LmjF.35.5360 | UD | E |  | DMQMT\|LmjF.35.4250 | UD | N |  | LSTO2r\|LmjF.23.1300 | UD | N |
| TRPabc\|LmjF.31.1790 | UD | E |  | DNMPPAn\|LmjF.24.2280 | UD | N |  | LYSPLpc_LM\|LmjF.24.1840 | UD | N |
| TRPabc\|LmjF.27.0680 | UD | E |  | DNMPPAn\|LmjF.10.0170 | UD | N |  | LYSPLpe_LM\|LmjF.24.1840 | UD | N |
| TRYP\|LmjF.15.1120 | UD | E |  | DNMPPAn\|LmjF.13.0500 | UD | N |  | LYSTRS\|LmjF.15.0230 | UD | N |
| TRYP\|LmjF.15.1060 | UD | E |  | DNMPPAn\|LmjF.32.1530 | UD | N |  | LYSTRS\|LmjF.30.0130 | UD | N |
| TRYP\|LmjF.15.1160 | UD | E |  | DNMPPAn\|LmjF.34.2610 | UD | N |  | LYSt6\|LmjF.11.0520 | UD | N |
| TRYP\|LmjF.15.1080 | UD | E |  | DNTPPAn\|LmjF.32.1530 | UD | N |  | LYSt6\|LmjF.27.0670 | UD | N |
| TRYP\|LmjF.15.1100 | UD | E |  | DNTPPAn\|LmjF.34.2610 | UD | N |  | LYSt6\|LmjF.14.0320 | UD | N |
| TRYP\|LmjF.15.1140 | UD | E |  | DNTPPAn\|LmjF.24.2280 | UD | N |  | LYSt6\|LmjF.22.0230 | UD | N |
| TRYPg\|LmjF.15.1040 | UD | E |  | DNTPPAn\|LmjF.10.0170 | UD | N |  | MALCODC\|LmjF.07.0730 | UD | N |
| TRYPm\|LmjF.23.0040 | UD | E |  | DNTPPAn\|LmjF.13.0500 | UD | N |  | MALT1\|LmjF.18.0090 | UD | N |
| TRYS\|LmjF.27.1870 | UD | E |  | DPCOAK\|LmjF.22.1530 | UD | N |  | MAN1PT1\|LmjF.23.0110 | UD | N |
| TRYS\|LmjF.23.0460 | UD | E |  | DPMVD\|LmjF.18.0020 | UD | N |  | MAN6PI\|LmjF.32.1580 | UD | N |
| TRYS\|LmjF.36.4300 | UD | E |  | DPMVDx\|LmjF.18.0020 | UD | N |  | MAT6m\|LmjF.05.0520 | UD | N |
| TYRTA\|LmjF.36.2360 | UD | E |  | DRBKr\|LmjF.27.0420 | UD | N |  | MCC\|LmjF.31.3130 | UD | N |
| TYRTA\|LmjF.35.0820 | UD | E |  | DRPAr\|LmjF.06.1070 | UD | N |  | MCC\|LmjF.11.0590 | UD | N |
| TYRTA2\|LmjF.35.0820 | UD | E |  | DSATer\|LmjF.31.1780 | UD | N |  | MCMAT2m\|LmjF.33.2720 | UD | N |
| TYRTA2\|LmjF.36.2360 | UD | E |  | DTDPG4Ex\|LmjF.33.2300 | UD | N |  | MCMAT3m\|LmjF.33.2720 | UD | N |
| TYRabc\|LmjF.31.1800 | UD | E |  | DTMPK\|LmjF.10.0060 | UD | N |  | MCMAT4m\|LmjF.33.2720 | UD | N |
| TYRabc\|LmjF.27.1580 | UD | E |  | DURIK1m\|LmjF.21.1210 | UD | N |  | MCMAT5m\|LmjF.33.2720 | UD | N |
| TYRabc\|LmjF.33.1420 | UD | E |  | DUTPDPm\|LmjF.06.0560 | UD | N |  | MCMAT6m\|LmjF.33.2720 | UD | N |
| TYRabc\|LmjF.36.0420 | UD | E |  | DUTPDPn\|LmjF.06.0560 | UD | N |  | MCMAT7m\|LmjF.33.2720 | UD | N |
| TYRabc\|LmjF.31.1820 | UD | E |  | EAPT_LM\|LmjF.36.5900 | UD | N |  | MCMAT8m\|LmjF.33.2720 | UD | N |
| TYRabc\|LmjF.31.1790 | UD | E |  | EBP1r\|LmjF.16.1340 | UD | N |  | MDH\|LmjF.28.2860 | UD | N |
| TYRabc\|LmjF.35.5360 | UD | E |  | EBP2r\|LmjF.16.1340 | UD | N |  | MDHg\|LmjF.19.0710 | UD | N |
| TYRabc\|LmjF.27.0680 | UD | E |  | ECAPLH\|LmjF.26.2700 | UD | N |  | MDHm\|LmjF.34.0150 | UD | N |
| TYRabc\|LmjF.35.5350 | UD | E |  | ECOAH13g\|LmjF.18.0580 | UD | N |  | MDHm\|LmjF.34.0160 | UD | N |
| TYRabc\|LmjF.36.6830 | UD | E |  | ECOAH13g\|LmjF.32.3680 | UD | N |  | MDHm\|LmjF.34.0140 | UD | N |
| UAGDPr\|LmjF.33.2520 | UD | E |  | ECOAH13g\|LmjF.33.2600 | UD | N |  | MDHm\|LmjF.34.0130 | UD | N |
| UDPDPS\|LmjF.13.0020 | UD | E |  | ECOAH13m\|LmjF.35.0360 | UD | N |  | MDRPD\|LmjF.28.1840 | UD | N |
| UDPG4Ex\|LmjF.33.2300 | UD | E |  | ECOAH13m\|LmjF.29.2310 | UD | N |  | ME1x\|LmjF.24.0770 | UD | N |
| UDPGALM\|LmjF.18.0200 | UD | E |  | ECOAH13m\|LmjF.26.1550 | UD | N |  | METAT\|LmjF.30.3520 | UD | N |
| UPPRTr\|LmjF.34.1040 | UD | E |  | ECOAH14g\|LmjF.18.0580 | UD | N |  | METAT\|LmjF.30.3500 | UD | N |
| URIK1\|LmjF.31.2470 | UD | E |  | ECOAH14g\|LmjF.32.3680 | UD | N |  | METS\|LmjF.07.0090 | UD | N |
| URIK2\|LmjF.31.2470 | UD | E |  | ECOAH14g\|LmjF.33.2600 | UD | N |  | METTRSm\|LmjF.21.0810 | UD | N |
| URIK3\|LmjF.31.2470 | UD | E |  | ECOAH14m\|LmjF.26.1550 | UD | N |  | METt6\|LmjF.14.0320 | UD | N |
| URIK4\|LmjF.31.2470 | UD | E |  | ECOAH14m\|LmjF.29.2310 | UD | N |  | METt6\|LmjF.22.0230 | UD | N |
| URIK5\|LmjF.31.2470 | UD | E |  | ECOAH14m\|LmjF.35.0360 | UD | N |  | METt6\|LmjF.27.0670 | UD | N |
| URIK6\|LmjF.31.2470 | UD | E |  | ECOAH15g\|LmjF.33.2600 | UD | N |  | METt6\|LmjF.11.0520 | UD | N |
| URIK7\|LmjF.31.2470 | UD | E |  | ECOAH15g\|LmjF.18.0580 | UD | N |  | MEVK\|LmjF.31.0560 | UD | N |
| URIK8\|LmjF.31.2470 | UD | E |  | ECOAH15g\|LmjF.32.3680 | UD | N |  | MEVKx\|LmjF.31.0560 | UD | N |
| URIK9\|LmjF.31.2470 | UD | E |  | ECOAH15m\|LmjF.29.2310 | UD | N |  | MFAPS_LM\|LmjF.31.3120 | UD | N |
| URIRHn\|LmjF.18.0480 | UD | E |  | ECOAH15m\|LmjF.26.1550 | UD | N |  | MFAPSm_LM\|LmjF.31.3120 | UD | N |
| VALTAm\|LmjF.27.2030 | UD | E |  | ECOAH15m\|LmjF.35.0360 | UD | N |  | MGLYCL_LM\|LmjF.31.1140 | UD | N |
| VALTRS\|LmjF.30.3130 | UD | E |  | ECOAH16m\|LmjF.35.0360 | UD | N |  | MI1PP\|LmjF.17.1390 | UD | N |
| VALt6\|LmjF.35.4410 | UD | E |  | ECOAH16m\|LmjF.26.1550 | UD | N |  | MI1PSB\|LmjF.14.1360 | UD | N |
| XANMT\|LmjF.23.1200 | UD | E |  | ECOAH16m\|LmjF.29.2310 | UD | N |  | MI3PP\|LmjF.17.1390 | UD | N |
| XANMT\|LmjF.12.1270 | UD | E |  | ECOAH17m\|LmjF.35.0360 | UD | N |  | MI4PP\|LmjF.17.1390 | UD | N |
| XPRTgr\|LmjF.21.0850 | UD | E |  | ECOAH17m\|LmjF.26.1550 | UD | N |  | MMEm\|LmjF.26.0020 | UD | N |
| XYLKg\|LmjF.36.0260 | UD | E |  | ECOAH17m\|LmjF.29.2310 | UD | N |  | MMMm\|LmjF.27.0300 | UD | N |
| TYRTRS\|LmjF.14.1370 | E | N |  | ECOAH1m\|LmjF.26.1550 | UD | N |  | MSTa\|LmjF.05.0970 | UD | N |
| FBPg\|LmjF.04.1160 | N | N |  | ECOAH1m\|LmjF.29.2310 | UD | N |  | MTAP\|LmjF.05.0830 | UD | N |
| GALU\|LmjF.18.0990 | N | N |  | ECOAH1m\|LmjF.35.0360 | UD | N |  | MTHFC\|LmjF.30.2600 | UD | N |
| GF6PTAr\|LmjF.06.0950 | N | N |  | ECOAH2m\|LmjF.26.1550 | UD | N |  | MTHPTGHM\|LmjF.31.0010 | UD | N |
| ST14DMr\|LmjF.11.1100 | N | N |  | ECOAH2m\|LmjF.29.2310 | UD | N |  | MTRI\|LmjF.36.4930 | UD | N |
| MTHFD\|LmjF.30.2600 | UD | N |  | ECOAH2m\|LmjF.35.0360 | UD | N |  | NADH2-u6cm\|LmjF.17.0270 | UD | N |
| DHAPAx_LM\|LmjF.34.1090 | UD | N |  | ECOAH3m\|LmjF.29.2310 | UD | N |  | NADH2-u6cm\|LmjF.27.0740 | UD | N |
| DHFS\|LmjF.36.2610 | UD | N |  | ECOAH3m\|LmjF.26.1550 | UD | N |  | NADH2-u6cm\|LmjF.05.0980 | UD | N |
| MTHFR\|LmjF.36.6390 | UD | N |  | ECOAH3m\|LmjF.35.0360 | UD | N |  | NADH2-u6cm\|LmjF.18.1480 | UD | N |
| MTHFR1\|LmjF.36.6390 | UD | N |  | ECOAH4m\|LmjF.26.1550 | UD | N |  | NADH2-u6cm\|LmjF.36.5380 | UD | N |
| PHE4MOi\|LmjF.28.1280 | UD | N |  | ECOAH4m\|LmjF.29.2310 | UD | N |  | NADH2-u6cm\|LmjF.27.0290 | UD | N |
| PHE4MOi2\|LmjF.28.1280 | UD | N |  | ECOAH4m\|LmjF.35.0360 | UD | N |  | NADHDH\|LmjF.18.1480 | UD | N |
| SERPTr\|LmjF.34.3740 | UD | N |  | ECOAH5m\|LmjF.35.0360 | UD | N |  | NADHDH\|LmjF.27.0740 | UD | N |
| SERPTr\|LmjF.35.0320 | UD | N |  | ECOAH5m\|LmjF.26.1550 | UD | N |  | NADHDH\|LmjF.05.0980 | UD | N |
| 14BP1P2\|LmjF.31.2920 | UD | N |  | ECOAH5m\|LmjF.29.2310 | UD | N |  | NADHDH\|LmjF.17.0270 | UD | N |
| 2MBDTm\|LmjF.05.0180 | UD | N |  | ECOAH6m\|LmjF.35.0360 | UD | N |  | NADHDH\|LmjF.27.0290 | UD | N |
| 2MBDTm\|LmjF.25.0020 | UD | N |  | ECOAH6m\|LmjF.26.1550 | UD | N |  | NADK\|LmjF.06.0460 | UD | N |
| 2MPTm\|LmjF.05.0180 | UD | N |  | ECOAH6m\|LmjF.29.2310 | UD | N |  | NADS2i\|LmjF.32.1670 | UD | N |
| 2MPTm\|LmjF.25.0020 | UD | N |  | ECOAH7m\|LmjF.26.1550 | UD | N |  | NCATD\|LmjF.27.0090 | UD | N |
| 3ANTHMT\|LmjF.12.1270 | UD | N |  | ECOAH7m\|LmjF.29.2310 | UD | N |  | NCHEXH\|LmjF.26.0210 | UD | N |
| 3ANTHMT\|LmjF.23.1200 | UD | N |  | ECOAH7m\|LmjF.35.0360 | UD | N |  | NCHEXH\|LmjF.34.2140 | UD | N |
| 3DSPHRr\|LmjF.35.0330 | UD | N |  | ECOAH9m\|LmjF.29.2310 | UD | N |  | NCTPPRT\|LmjF.33.0960 | UD | N |
| 3MBTm\|LmjF.05.0180 | UD | N |  | ECOAH9m\|LmjF.26.1550 | UD | N |  | NDPK1\|LmjF.35.3870 | UD | N |
| 3MBTm\|LmjF.25.0020 | UD | N |  | ECOAH9m\|LmjF.35.0360 | UD | N |  | NDPK1\|LmjF.32.2950 | UD | N |
| 4COUCOAL\|LmjF.19.1005 | UD | N |  | ENO\|LmjF.32.3760 | UD | N |  | NDPK10\|LmjF.32.2950 | UD | N |
| 4COUCOAL\|LmjF.19.0985 | UD | N |  | ENO\|LmjF.14.1160 | UD | N |  | NDPK10\|LmjF.35.3870 | UD | N |
| 4HGLSDm\|LmjF.03.0200 | UD | N |  | ENPHE1\|LmjF.36.5910 | UD | N |  | NDPK2\|LmjF.32.2950 | UD | N |
| 4HTHRS\|LmjF.14.0350 | UD | N |  | EPPP2\|LmjF.01.0310 | UD | N |  | NDPK2\|LmjF.35.3870 | UD | N |
| 5OPROS\|LmjF.18.1040 | UD | N |  | EPPP2v\|LmjF.01.0310 | UD | N |  | NDPK3\|LmjF.35.3870 | UD | N |
| A1E\|LmjF.35.0970 | UD | N |  | ETAPCT\|LmjF.32.0890 | UD | N |  | NDPK3\|LmjF.32.2950 | UD | N |
| A1E\|LmjF.23.0430 | UD | N |  | ETAPCTm\|LmjF.32.0890 | UD | N |  | NDPK4\|LmjF.35.3870 | UD | N |
| A1Eg\|LmjF.35.0980 | UD | N |  | ETHAMK\|LmjF.27.1420 | UD | N |  | NDPK4\|LmjF.32.2950 | UD | N |
| A1Eg\|LmjF.34.3380 | UD | N |  | ETHAPT_LM\|LmjF.36.5900 | UD | N |  | NDPK5\|LmjF.35.3870 | UD | N |
| ACACT10m_i\|LmjF.31.1640 | UD | N |  | ETHAPTm_LM\|LmjF.36.5900 | UD | N |  | NDPK5\|LmjF.32.2950 | UD | N |
| ACACT10m_i\|LmjF.31.1630 | UD | N |  | FACOAL1\|LmjF.03.0230 | UD | N |  | NDPK6\|LmjF.32.2950 | UD | N |
| ACACT10m_i\|LmjF.23.0690 | UD | N |  | FACOAL1\|LmjF.01.0490 | UD | N |  | NDPK6\|LmjF.35.3870 | UD | N |
| ACACT1rm\|LmjF.31.1640 | UD | N |  | FACOAL1\|LmjF.01.0530 | UD | N |  | NDPK7\|LmjF.32.2950 | UD | N |
| ACACT1rm\|LmjF.31.1630 | UD | N |  | FACOAL1\|LmjF.13.0420 | UD | N |  | NDPK7\|LmjF.35.3870 | UD | N |
| ACACT1rm\|LmjF.23.0690 | UD | N |  | FACOAL1\|LmjF.01.0470 | UD | N |  | NDPK8\|LmjF.35.3870 | UD | N |
| ACACT2rm\|LmjF.31.1640 | UD | N |  | FACOAL1\|LmjF.01.0510 | UD | N |  | NDPK8\|LmjF.32.2950 | UD | N |
| ACACT2rm\|LmjF.23.0690 | UD | N |  | FACOAL1\|LmjF.01.0520 | UD | N |  | NDPK9\|LmjF.35.3870 | UD | N |
| ACACT2rm\|LmjF.31.1630 | UD | N |  | FACOAL1\|LmjF.01.0500 | UD | N |  | NDPK9\|LmjF.32.2950 | UD | N |
| ACACT3rm\|LmjF.31.1640 | UD | N |  | FACOAL140\|LmjF.13.0420 | UD | N |  | NDPKn1\|LmjF.32.2950 | UD | N |
| ACACT3rm\|LmjF.23.0690 | UD | N |  | FACOAL140\|LmjF.01.0490 | UD | N |  | NDPKn1\|LmjF.35.3870 | UD | N |
| ACACT3rm\|LmjF.31.1630 | UD | N |  | FACOAL140\|LmjF.03.0230 | UD | N |  | NDPKn10\|LmjF.35.3870 | UD | N |
| ACACT4rm\|LmjF.31.1630 | UD | N |  | FACOAL140\|LmjF.01.0530 | UD | N |  | NDPKn10\|LmjF.32.2950 | UD | N |
| ACACT4rm\|LmjF.23.0690 | UD | N |  | FACOAL140\|LmjF.01.0500 | UD | N |  | NDPKn2\|LmjF.35.3870 | UD | N |
| ACACT4rm\|LmjF.31.1640 | UD | N |  | FACOAL140\|LmjF.01.0510 | UD | N |  | NDPKn2\|LmjF.32.2950 | UD | N |
| ACACT5rm\|LmjF.31.1630 | UD | N |  | FACOAL140\|LmjF.01.0470 | UD | N |  | NDPKn3\|LmjF.35.3870 | UD | N |
| ACACT5rm\|LmjF.23.0690 | UD | N |  | FACOAL140\|LmjF.01.0520 | UD | N |  | NDPKn3\|LmjF.32.2950 | UD | N |
| ACACT5rm\|LmjF.31.1640 | UD | N |  | FACOAL160\|LmjF.03.0230 | UD | N |  | NDPKn4\|LmjF.35.3870 | UD | N |
| ACACT6rm\|LmjF.31.1640 | UD | N |  | FACOAL160\|LmjF.01.0530 | UD | N |  | NDPKn4\|LmjF.32.2950 | UD | N |
| ACACT6rm\|LmjF.23.0690 | UD | N |  | FACOAL160\|LmjF.01.0490 | UD | N |  | NDPKn5\|LmjF.35.3870 | UD | N |
| ACACT6rm\|LmjF.31.1630 | UD | N |  | FACOAL160\|LmjF.13.0420 | UD | N |  |  |  |  |
| ACACT7rm\|LmjF.31.1640 | UD | N |  | FACOAL160\|LmjF.01.0520 | UD | N |  |  |  |  |
| ACACT7rm\|LmjF.23.0690 | UD | N |  | FACOAL160\|LmjF.01.0510 | UD | N |  |  |  |  |
| ACACT7rm\|LmjF.31.1630 | UD | N |  | FACOAL160\|LmjF.01.0470 | UD | N |  |  |  |  |
| ACCOACrm\|LmjF.31.2970 | UD | N |  | FACOAL160\|LmjF.01.0500 | UD | N |  |  |  |  |
| ACGAM6PSi\|LmjF.28.3005 | UD | N |  | FACOAL180\|LmjF.13.0420 | UD | N |  |  |  |  |
| ACGAMPM\|LmjF.07.0805 | UD | N |  | FACOAL180\|LmjF.03.0230 | UD | N |  |  |  |  |
| ACMAT1m\|LmjF.33.2720 | UD | N |  | FACOAL180\|LmjF.01.0530 | UD | N |  |  |  |  |
| ACOAD10m\|LmjF.06.0880 | UD | N |  | FACOAL180\|LmjF.01.0490 | UD | N |  |  |  |  |
| ACOAD10m\|LmjF.28.2510 | UD | N |  | FACOAL180\|LmjF.01.0500 | UD | N |  |  |  |  |
| ACOAD11m\|LmjF.06.0880 | UD | N |  | FACOAL180\|LmjF.01.0520 | UD | N |  |  |  |  |
| ACOAD11m\|LmjF.28.2510 | UD | N |  | FACOAL180\|LmjF.01.0470 | UD | N |  |  |  |  |
|  |  |  |  |  |  |  |  |  |  |  |
