# Supplementary material for: Essential gene prediction using limited gene essentiality information–An integrative semi-supervised machine learning strategy
Source: PLoS One. 2020 Nov 30;15(11):e0242943. doi: 10.1371/journal.pone.0242943 (PMC7703937; doi:10.1371/journal.pone.0242943)
Supplement: S7 Table — (DOCX) [file pone.0242943.s011.docx]

**S7 Table. Gene Ontology (Molecular Function) terms of the predicted essential genes in *Leishmania donovani***

| **Gene ontology (Molecular Function)** | **Number of Genes** | **Gene List (Uniprot IDs)** |
| --- | --- | --- |
| ATP binding [GO:0005524] | 11 | [E9BKD6,E9BCR2,E9BG78,E9BUS2,E9BP61,E9BSR1,E9BDY2,E9BTY3,E9BI02,E9BTK0,E9BT25] |
| oxidoreductase activity [GO:0016491] | 8 | [E9BFX7,E9BJC7,E9BH73,E9BD53,E9BUR9,E9BPY2,E9BJC4,E9BKH5] |
| AMP deaminase activity [GO:0003876] | 4 | [E9B7Z3,E9BBG1,E9BP20,E9BT05] |
| flavin adenine dinucleotide binding [GO:0050660] | 4 | [E9B8V1,E9B911,E9BJZ0,E9BSE8] |
| metal ion binding [GO:0046872] | 4 | [E9BCR2,E9B8I8,E9BD53,E9BIZ2] |
| NADH dehydrogenase (ubiquinone) activity [GO:0008137] | 3 | [E9B8I8,E9BDX7,E9BIZ2] |
| catalytic activity [GO:0003824] | 3 | [E9BB03,E9BD62,E9BNX3] |
| pyridoxal phosphate binding [GO:0030170] | 3 | [E9BSJ9,E9BB38,E9BRW0] |
| 2 iron, 2 sulfur cluster binding [GO:0051537] | 2 | [E9BS30,E9BD53] |
| 4 iron, 4 sulfur cluster binding [GO:0051539] | 2 | [E9B8I8,E9BIZ2] |
| FAD binding [GO:0071949] | 2 | [E9BJC4,E9BKH5] |
| NAD+ kinase activity [GO:0003951] | 2 | [E9BI81,E9BT25] |
| acyl-CoA dehydrogenase activity [GO:0003995] | 2 | [E9B8V1,E9BSE8] |
| adenylate kinase activity [GO:0004017] | 2 | [E9BI02,E9BTK0] |
| diacylglycerol kinase activity [GO:0004143] | 2 | [E9BCY8,E9BT25] |
| electron transfer activity [GO:0009055] | 2 | [E9B917,E9BDX7] |
| hydrolase activity [GO:0016787] | 2 | [E9BH53,E9B7B1] |
| nucleoside diphosphate kinase activity [GO:0004550] | 2 | [E9BP61,E9BSR1] |
| oxidoreductase activity, acting on the CH-CH group of donors [GO:0016627] | 2 | [E9B911,E9BJZ0] |
| oxidoreductase activity, acting on the aldehyde or oxo group of donors, NAD or NADP as acceptor [GO:0016620] | 2 | [E9BM35,E9BUP9] |
| transaminase activity [GO:0008483] | 2 | [E9BB38,E9BRW0] |
| transferase activity, transferring glycosyl groups [GO:0016757] | 2 | [E9BQR2,E9BF85] |
| ubiquinol-cytochrome-c reductase activity [GO:0008121] | 2 | [E9BN35,E9BS30] |
| zinc ion binding [GO:0008270] | 2 | [E9BKN9,E9BPY2] |
| (S)-2-(5-amino-1-(5-phospho-D-ribosyl)imidazole-4-carboxamido)succinate AMP-lyase (fumarate-forming) activity [GO:0070626] | 1 | [E9B810] |
| 3-beta-hydroxy-delta5-steroid dehydrogenase activity [GO:0003854] | 1 | [E9B8P6] |
| AMP binding [GO:0016208] | 1 | [E9BG78] |
| FMN binding [GO:0010181] | 1 | [E9B8I8] |
| N-acetyltransferase activity [GO:0008080] | 1 | [E9BHA6] |
| N6-(1,2-dicarboxyethyl)AMP AMP-lyase (fumarate-forming) activity [GO:0004018] | 1 | [E9B810] |
| NAD binding [GO:0051287] | 1 | [E9B8I8] |
| O-acyltransferase activity [GO:0008374] | 1 | [E9BQR8] |
| acetate-CoA ligase activity [GO:0003987] | 1 | [E9BG78] |
| acireductone synthase activity [GO:0043874] | 1 | [E9BUX4] |
| argininosuccinate synthase activity [GO:0004055] | 1 | [E9BCR2] |
| carbamoyl-phosphate synthase (glutamine-hydrolyzing) activity [GO:0004088] | 1 | [E9BCR2] |
| coproporphyrinogen oxidase activity [GO:0004109] | 1 | [E9B8Z2] |
| guanine deaminase activity [GO:0008892] | 1 | [E9BKN9] |
| heme binding [GO:0020037] | 1 | [E9B917] |
| iron-sulfur cluster binding [GO:0051536] | 1 | [E9BDX7] |
| kinase activity [GO:0016301] | 1 | [E9BT87] |
| lyase activity [GO:0016829] | 1 | [E9BSJ9] |
| magnesium ion binding [GO:0000287] | 1 | [E9BUX4] |
| nicotinate phosphoribosyltransferase activity [GO:0004516] | 1 | [E9BQ30] |
| nicotinate-nucleotide diphosphorylase (carboxylating) activity [GO:0004514] | 1 | [E9BQ30] |
| phosphotransferase activity, alcohol group as acceptor [GO:0016773] | 1 | [E9BT87] |
| proton-exporting ATPase activity, phosphorylative mechanism [GO:0008553] | 1 | [E9BDY2] |
| quinone binding [GO:0048038] | 1 | [E9BIZ2] |
| ribonucleoside-diphosphate reductase activity, thioredoxin disulfide as acceptor [GO:0004748] | 1 | [E9BKD6] |
| tetrahydrofolylpolyglutamate synthase activity [GO:0004326] | 1 | [E9BTY3] |
| transferase activity [GO:0016740] | 1 | [E9B822] |
| transferase activity, transferring acyl groups [GO:0016746] | 1 | [E9B8A8] |
