# Supplementary material for: Essential gene prediction using limited gene essentiality information–An integrative semi-supervised machine learning strategy
Source: PLoS One. 2020 Nov 30;15(11):e0242943. doi: 10.1371/journal.pone.0242943 (PMC7703937; doi:10.1371/journal.pone.0242943)
Supplement: S8 Table — (DOCX) [file pone.0242943.s012.docx]

**S8 Table. Gene Ontology (Molecular Function) terms of the predicted essential genes in *Leishmania major***

| **Gene ontology (Molecular Function)** | **Number of Genes** | **Gene List (Uniprot IDs)** |
| --- | --- | --- |
| ATP binding [GO:0005524] | 43 | [Q9U1E1,E9AFL1,Q4Q614,Q4Q8H8,Q4Q273,Q4QD33,Q4QD34,Q4Q6V1,E9AEB3,Q4Q1G0,E9ADK8,Q4QBE4,Q4QDS1,Q4Q288,E9AC86,Q4QCT4,Q4Q2E8,Q4Q2N5,E9AE77,Q4Q7P6,Q4Q7S2,Q4Q598,Q4QIS7,Q4QH55,E9ACY7,Q4QG76,Q4Q0Y9,Q4Q8Q6,Q4QF34,Q4QH70,E9ADF8,E9ADF9,Q4QGX9,Q4QC75,Q4Q1C4,Q4Q5J5,Q4QDB1,Q4Q6X7,E9AEW4,E9ABZ4,Q4QFJ7,E9ACM5,Q4Q9G0] |
| magnesium ion binding [GO:0000287] | 13 | [Q4QCF1,E9ACN6,Q4QH59,Q4Q1C4,E9AEH9,E9AEI0,Q4QDB1,Q4QCC2,Q4Q2G5,Q4QIB8,Q4QI56,Q4Q0M2,Q4Q3Z4] |
| amino acid transmembrane transporter activity [GO:0015171] | 12 | [E9AD44,Q4QBX3,E9AG08,Q4Q072,Q4Q682,E9ADD7,Q4Q445,Q4Q236,Q4Q680,E9AG09,Q4Q683,E9AD45] |
| metal ion binding [GO:0046872] | 10 | [E9AEB3,Q4QAC4,Q4Q7B0,Q4QGX9,Q4QC75,Q4Q7P5,Q4Q431,E9ABZ4,E9AFJ2,Q4Q842] |
| protein serine/threonine kinase activity [GO:0004674] | 10 | [Q4Q288,E9AC86,Q4QCT4,Q4Q2E8,Q4Q7P6,Q4Q598,Q4QIS7,Q4QH55,E9ACY7,Q4Q0Y9] |
| flavin adenine dinucleotide binding [GO:0050660] | 9 | [Q4QFZ2,Q4QAG8,Q4QJG7,Q4Q5Z6,Q4Q4U1,E9AE44,Q4Q5Z7,Q4QIY9,Q4Q812] |
| kinase activity [GO:0016301] | 7 | [Q4Q614,E9ADF8,E9ADF9,Q4QGX9,E9AEH9,E9AEI0,Q4QIB8] |
| inorganic phosphate transmembrane transporter activity [GO:0005315] | 6 | [Q4QJH3,E9AFR9,E9AFS0,E9ACJ5,Q4QHL7,Q4QH82] |
| pyridoxal phosphate binding [GO:0030170] | 6 | [Q4Q1I5,Q4FX34,E9AFE7,Q4QAU4,Q4Q758,Q4Q159] |
| ADP binding [GO:0043531] | 5 | [Q4QD33,Q4QD34,Q4Q6V1,E9AEH9,E9AEI0] |
| electron transfer activity [GO:0009055] | 5 | [Q4QAG8,Q4QJG7,Q4Q5Z6,Q4Q4U1,E9AE44] |
| thioredoxin peroxidase activity [GO:0008379] | 5 | [Q4QBH2,Q4QF80,Q4QF68,Q4QF76,Q4QF74] |
| 1-phosphatidylinositol-3-kinase activity [GO:0016303] | 4 | [Q4QAC9,Q4QCT4,Q4Q7B0,Q4Q2E8] |
| RNA binding [GO:0003723] | 4 | [Q4QDB1,Q4Q9E9,Q4Q1Q8,Q4Q7E9] |
| beta-fructofuranosidase activity [GO:0004564] | 4 | [Q4QB76,E9ACV4,Q4QB75,Q9XTP3] |
| hydrolase activity [GO:0016787] | 4 | [E9AG08,E9AG09,E9AFR8,Q4Q546] |
| ligase activity [GO:0016874] | 4 | [Q711P7,Q4QBC8,Q4Q0Y4,E9ABZ4] |
| oxidoreductase activity [GO:0016491] | 4 | [Q4QBL8,Q5EEK0,Q4QAG8,Q4Q7P5] |
| peroxiredoxin activity [GO:0051920] | 4 | [Q4QF80,Q4QF68,Q4QF76,Q4QF74] |
| ribose phosphate diphosphokinase activity [GO:0004749] | 4 | [Q4QIB8,Q4QI56,Q4Q0M2,Q4Q3Z4] |
