# Supplementary material for: Essential gene prediction using limited gene essentiality information–An integrative semi-supervised machine learning strategy
Source: PLoS One. 2020 Nov 30;15(11):e0242943. doi: 10.1371/journal.pone.0242943 (PMC7703937; doi:10.1371/journal.pone.0242943)
Supplement: S9 Table — (DOCX) [file pone.0242943.s013.docx]

**S9 Table. KEGG Pathway enrichment of the predicted essential genes in *Leishmania donovani***

| **Term** | **PValue** | **Genes** |
| --- | --- | --- |
| ldo01100:Metabolic pathways | 1.25198E-11 | LDBPK_230580, LDBPK_061330, LDBPK_352010, LDBPK_350100, LDBPK_181460, LDBPK_362740, LDBPK_290920, LDBPK_170410, LDBPK_341110, LDBPK_060910, LDBPK_271970, LDBPK_323110, LDBPK_280980, LDBPK_270590, LDBPK_270300, LDBPK_120580, LDBPK_050180, LDBPK_160590, LDBPK_070210, LDBPK_040440, LDBPK_272390, LDBPK_365650, LDBPK_353910, LDBPK_350840, LDBPK_040570, LDBPK_361410, LDBPK_331010, LDBPK_252480, LDBPK_050980, LDBPK_354860, LDBPK_355060, LDBPK_120105, LDBPK_322690, LDBPK_230880, LDBPK_170320, LDBPK_351540, LDBPK_312650 |
| ldo00190:Oxidative phosphorylation | 2.23446E-07 | LDBPK_352010, LDBPK_181510, LDBPK_365620, LDBPK_070210, LDBPK_350100, LDBPK_050980, LDBPK_181460, LDBPK_170320, LDBPK_270590, LDBPK_351540, LDBPK_270300, LDBPK_312650 |
| ldo00230:Purine metabolism | 0.000647072 | LDBPK_252480, LDBPK_361410, LDBPK_271970, LDBPK_040440, LDBPK_354860, LDBPK_323110, LDBPK_280980, LDBPK_322690, LDBPK_353910, LDBPK_290920 |
| ldo01110:Biosynthesis of secondary metabolites | 0.002288788 | LDBPK_230580, LDBPK_050180, LDBPK_061330, LDBPK_040440, LDBPK_353910, LDBPK_350840, LDBPK_060910, LDBPK_361410, LDBPK_252480, LDBPK_354860, LDBPK_355060, LDBPK_120105, LDBPK_323110, LDBPK_230880, LDBPK_322690 |
| ldo01130:Biosynthesis of antibiotics | 0.004028807 | LDBPK_230580, LDBPK_050180, LDBPK_252480, LDBPK_361410, LDBPK_060910, LDBPK_040440, LDBPK_354860, LDBPK_323110, LDBPK_120105, LDBPK_322690, LDBPK_230880, LDBPK_353910, LDBPK_350840 |
| ldo00240:Pyrimidine metabolism | 0.006067033 | LDBPK_170410, LDBPK_160590, LDBPK_341110, LDBPK_271970, LDBPK_323110, LDBPK_280980, LDBPK_353910 |
| ldo00250:Alanine, aspartate and glutamate metabolism | 0.024948804 | LDBPK_120580, LDBPK_160590, LDBPK_040440, LDBPK_350840 |
| ldo00480:Glutathione metabolism | 0.038593104 | LDBPK_040570, LDBPK_271970, LDBPK_120105, LDBPK_280980 |
| ldo00620:Pyruvate metabolism | 0.082188661 | LDBPK_230580, LDBPK_120200, LDBPK_271940, LDBPK_230880 |
| ldo00330:Arginine and proline metabolism | 0.098738561 | LDBPK_040570, LDBPK_120105, LDBPK_350840 |
| ldo00640:Propanoate metabolism | 0.098738561 | LDBPK_230580, LDBPK_060910, LDBPK_230880 |
