# Supplementary material for: Essential gene prediction using limited gene essentiality information–An integrative semi-supervised machine learning strategy
Source: PLoS One. 2020 Nov 30;15(11):e0242943. doi: 10.1371/journal.pone.0242943 (PMC7703937; doi:10.1371/journal.pone.0242943)
Supplement: S10 Table — (DOCX) [file pone.0242943.s014.docx]

**S10 Table. KEGG Pathway enrichment of the predicted essential genes in *Leishmania major***

| **Term** | **PValue** | **Genes** |
| --- | --- | --- |
| lma01100:Metabolic pathways | 8.16878E-39 | LMJF_33_2300, LMJF_35_0030, LMJF_15_1060, LMJF_36_1760, LMJF_23_0880, LMJF_35_4590, LMJF_06_1280, LMJF_33_1570, LMJF_06_0880, LMJF_21_1430, LMJF_36_1960, LMJF_21_1210, LMJF_27_2340, LMJF_26_2480, LMJF_31_2940, LMJF_23_0870, LMJF_31_2640, LMJF_15_1120, LMJF_24_2060, LMJF_30_1250, LMJF_05_0180, LMJF_16_0550, LMJF_32_2950, LMJF_36_2360, LMJF_29_2800, LMJF_30_2350, LMJF_24_2010, LMJF_25_1710, LMJF_20_0110, LMJF_15_1080, LMJF_34_2850, LMJF_25_2140, LMJF_34_3590, LMJF_26_1620, LMJF_20_0100, LMJF_31_2650, LMJF_34_3780, LMJF_36_3590, LMJF_06_0860, LMJF_13_1620, LMJF_08_1130, LMJF_30_1540, LMJF_04_0580, LMJF_30_2950, LMJF_05_0350, LMJF_35_0020, LMJF_03_0200, LMJF_12_0530, LMJF_21_0550, LMJF_36_2660, LMJF_31_3120, LMJF_20_1120, LMJF_36_2610, LMJF_25_2130, LMJF_15_1100, LMJF_29_2510, LMJF_12_0280, LMJF_36_2380, LMJF_22_1290, LMJF_24_1630, LMJF_14_0350, LMJF_15_1160, LMJF_24_0370, LMJF_27_2050, LMJF_13_1680, LMJF_26_0210, LMJF_34_1040, LMJF_36_0260, LMJF_27_1870, LMJF_36_3810, LMJF_11_1000, LMJF_28_0890, LMJF_36_0060, LMJF_03_0030, LMJF_33_1930, LMJF_31_2470, LMJF_29_1830, LMJF_30_1850, LMJF_33_2520, LMJF_08_0060, LMJF_35_0820, LMJF_30_3380, LMJF_35_3340, LMJF_31_2290, LMJF_15_1040, LMJF_35_0050, LMJF_36_5390, LMJF_36_3800, LMJF_08_0510, LMJF_16_0760, LMJF_15_1140, LMJF_23_0040, LMJF_27_2030, LMJF_15_1460, LMJF_18_0440, LMJF_36_6650, LMJF_28_1970, LMJF_01_0050, LMJF_24_0850, LMJF_35_3020, LMJF_34_3090, LMJF_32_3310, LMJF_36_2950, LMJF_35_3680, LMJF_27_1805, LMJF_35_4250, LMJF_28_0140, LMJF_21_0640, LMJF_27_1810, LMJF_01_0480, LMJF_28_0490, LMJF_18_1380, LMJF_35_3870 |
| lma01110:Biosynthesis of secondary metabolites | 4.55232E-27 | LMJF_35_0030, LMJF_36_2380, LMJF_24_1630, LMJF_14_0350, LMJF_35_4590, LMJF_06_1280, LMJF_24_0370, LMJF_33_1570, LMJF_06_0880, LMJF_21_1430, LMJF_13_1680, LMJF_36_1960, LMJF_36_3810, LMJF_31_2940, LMJF_31_2640, LMJF_33_1930, LMJF_29_1830, LMJF_24_2060, LMJF_05_0180, LMJF_08_0060, LMJF_35_0820, LMJF_32_2950, LMJF_30_3380, LMJF_35_3340, LMJF_31_2290, LMJF_36_2360, LMJF_35_0050, LMJF_36_3800, LMJF_36_5390, LMJF_08_0510, LMJF_25_1710, LMJF_20_0110, LMJF_16_0760, LMJF_34_2850, LMJF_25_2140, LMJF_27_2030, LMJF_15_1460, LMJF_18_0440, LMJF_20_0100, LMJF_26_1620, LMJF_36_6650, LMJF_28_1970, LMJF_31_2650, LMJF_34_3780, LMJF_36_3590, LMJF_08_1130, LMJF_13_1620, LMJF_24_0850, LMJF_35_3020, LMJF_35_0020, LMJF_32_3310, LMJF_21_0550, LMJF_12_0530, LMJF_36_2660, LMJF_36_2950, LMJF_35_3680, LMJF_27_1805, LMJF_35_4250, LMJF_31_3120, LMJF_21_0640, LMJF_27_1810, LMJF_25_2130, LMJF_01_0480, LMJF_18_1380, LMJF_35_3870, LMJF_29_2510, LMJF_12_0280 |
| lma01130:Biosynthesis of antibiotics | 1.40204E-27 | LMJF_35_0030, LMJF_36_2380, LMJF_24_1630, LMJF_24_0370, LMJF_33_1570, LMJF_06_0880, LMJF_21_1430, LMJF_13_1680, LMJF_36_3810, LMJF_31_2940, LMJF_03_0030, LMJF_31_2640, LMJF_33_1930, LMJF_29_1830, LMJF_24_2060, LMJF_05_0180, LMJF_33_2520, LMJF_35_0820, LMJF_08_0060, LMJF_32_2950, LMJF_30_3380, LMJF_35_3340, LMJF_36_2360, LMJF_35_0050, LMJF_36_3800, LMJF_36_5390, LMJF_26_2230, LMJF_08_0510, LMJF_25_1710, LMJF_20_0110, LMJF_16_0760, LMJF_25_2140, LMJF_27_2030, LMJF_15_1460, LMJF_20_0100, LMJF_36_6650, LMJF_28_1970, LMJF_31_2650, LMJF_34_3780, LMJF_01_0050, LMJF_36_3590, LMJF_13_1620, LMJF_08_1130, LMJF_24_0850, LMJF_35_3020, LMJF_35_0020, LMJF_21_0550, LMJF_12_0530, LMJF_32_3310, LMJF_36_2660, LMJF_36_2950, LMJF_35_3680, LMJF_27_1805, LMJF_21_0640, LMJF_27_1810, LMJF_25_2130, LMJF_01_0480, LMJF_28_0490, LMJF_18_1380, LMJF_35_3870, LMJF_29_2510, LMJF_12_0280 |
| lma01200:Carbon metabolism | 2.24838E-24 | LMJF_30_3380, LMJF_35_3340, LMJF_35_0030, LMJF_36_5390, LMJF_36_3800, LMJF_08_0510, LMJF_24_1630, LMJF_25_1710, LMJF_20_0110, LMJF_16_0760, LMJF_24_0370, LMJF_33_1570, LMJF_06_0880, LMJF_34_2850, LMJF_25_2140, LMJF_20_0100, LMJF_36_6650, LMJF_28_1970, LMJF_31_2650, LMJF_01_0050, LMJF_36_3590, LMJF_08_1130, LMJF_24_0850, LMJF_36_3810, LMJF_35_0020, LMJF_11_1000, LMJF_12_0530, LMJF_32_3310, LMJF_21_0550, LMJF_36_2660, LMJF_36_2950, LMJF_35_3680, LMJF_27_1805, LMJF_03_0030, LMJF_31_2640, LMJF_33_1930, LMJF_29_1830, LMJF_27_1810, LMJF_25_2130, LMJF_24_2060, LMJF_28_0490, LMJF_18_1380, LMJF_08_0060, LMJF_35_0820, LMJF_29_2510 |
| lma01230:Biosynthesis of amino acids | 6.17941E-13 | LMJF_30_3380, LMJF_35_0030, LMJF_36_2360, LMJF_36_5390, LMJF_08_0510, LMJF_20_0110, LMJF_16_0760, LMJF_14_0350, LMJF_24_0370, LMJF_33_1570, LMJF_34_2850, LMJF_27_2030, LMJF_13_1680, LMJF_36_6650, LMJF_20_0100, LMJF_28_1970, LMJF_36_3590, LMJF_08_1130, LMJF_24_0850, LMJF_35_0020, LMJF_35_3680, LMJF_03_0030, LMJF_33_1930, LMJF_24_2060, LMJF_01_0480, LMJF_35_0820, LMJF_08_0060, LMJF_29_2510 |
| lma00010:Glycolysis / Gluconeogenesis | 5.84107E-12 | LMJF_30_3380, LMJF_35_0030, LMJF_35_0020, LMJF_32_3310, LMJF_12_0530, LMJF_21_0550, LMJF_36_2660, LMJF_25_1710, LMJF_20_0110, LMJF_27_1805, LMJF_31_2640, LMJF_21_0640, LMJF_29_1830, LMJF_27_1810, LMJF_36_6650, LMJF_20_0100, LMJF_31_2650, LMJF_34_3780, LMJF_18_1380, LMJF_24_0850, LMJF_08_0060, LMJF_29_2510 |
| lma00480:Glutathione metabolism | 9.24113E-09 | LMJF_35_3340, LMJF_05_0350, LMJF_15_1040, LMJF_27_1870, LMJF_15_1060, LMJF_28_0890, LMJF_22_1290, LMJF_15_1160, LMJF_15_1080, LMJF_15_1140, LMJF_23_0040, LMJF_27_2050, LMJF_15_1120, LMJF_04_0580, LMJF_15_1100, LMJF_12_0280 |
| lma00230:Purine metabolism | 0.035214819 | LMJF_35_0030, LMJF_36_5390, LMJF_29_2800, LMJF_35_0020, LMJF_28_0890, LMJF_08_0510, LMJF_22_1290, LMJF_33_1930, LMJF_27_2050, LMJF_21_0640, LMJF_34_3780, LMJF_08_1130, LMJF_35_3870, LMJF_32_2950 |
| lma00020:Citrate cycle (TCA cycle) | 2.30064E-06 | LMJF_31_2640, LMJF_29_1830, LMJF_27_1810, LMJF_25_2140, LMJF_25_2130, LMJF_31_2650, LMJF_32_3310, LMJF_21_0550, LMJF_36_2660, LMJF_36_2950, LMJF_18_1380, LMJF_25_1710, LMJF_24_1630, LMJF_27_1805 |
| lma00280:Valine, leucine and isoleucine degradation | 5.07784E-07 | LMJF_35_0050, LMJF_32_3310, LMJF_31_2640, LMJF_30_1940, LMJF_29_1830, LMJF_06_0880, LMJF_21_1430, LMJF_27_2030, LMJF_31_2650, LMJF_05_0180, LMJF_28_0490, LMJF_01_0050, LMJF_30_1930, LMJF_33_2340 |
| lma00030:Pentose phosphate pathway | 1.43273E-09 | LMJF_35_3340, LMJF_36_5390, LMJF_12_0530, LMJF_08_0510, LMJF_16_0760, LMJF_35_3680, LMJF_33_1930, LMJF_33_1570, LMJF_21_0640, LMJF_28_1970, LMJF_24_2060, LMJF_34_3780, LMJF_08_1130, LMJF_29_2510 |
| lma00620:Pyruvate metabolism | 1.59149E-05 | LMJF_35_0030, LMJF_35_0020, LMJF_11_1000, LMJF_32_3310, LMJF_21_0550, LMJF_36_2660, LMJF_25_1710, LMJF_27_1805, LMJF_31_2640, LMJF_29_1830, LMJF_27_1810, LMJF_31_2650, LMJF_18_1380 |
| lma00260:Glycine, serine and threonine metabolism | 4.21184E-06 | LMJF_31_2640, LMJF_29_1830, LMJF_36_3800, LMJF_36_3810, LMJF_36_6650, LMJF_31_2650, LMJF_01_0480, LMJF_32_3310, LMJF_08_0060, LMJF_14_0350, LMJF_03_0030 |
| lma00240:Pyrimidine metabolism | 0.046498998 | LMJF_31_2470, LMJF_27_2050, LMJF_28_0890, LMJF_06_0860, LMJF_22_1290, LMJF_35_3870, LMJF_16_0550, LMJF_21_1210, LMJF_32_2950, LMJF_34_1040 |
| lma00630:Glyoxylate and dicarboxylate metabolism | 0.01064073 | LMJF_31_2640, LMJF_29_1830, LMJF_36_3800, LMJF_36_3810, LMJF_31_2650, LMJF_32_3310, LMJF_01_0050, LMJF_28_0490 |
| lma04070:Phosphatidylinositol signaling system | 0.0019012 | LMJF_34_3590, LMJF_26_1620, LMJF_34_3090, LMJF_30_1850, LMJF_24_2010, LMJF_26_2480, LMJF_30_2950, LMJF_20_1120 |
| lma00562:Inositol phosphate metabolism | 0.000202568 | LMJF_34_3590, LMJF_34_3090, LMJF_30_1850, LMJF_24_2010, LMJF_26_2480, LMJF_24_0850, LMJF_30_2950, LMJF_20_1120 |
| lma00052:Galactose metabolism | 0.003688894 | LMJF_33_2300, LMJF_21_0640, LMJF_34_3780, LMJF_23_0880, LMJF_27_2340, LMJF_29_2510, LMJF_23_0870 |
| lma00564:Glycerophospholipid metabolism | 0.031259793 | LMJF_31_2290, LMJF_26_1620, LMJF_18_0440, LMJF_26_2480, LMJF_35_4590, LMJF_31_3120 |
| lma00330:Arginine and proline metabolism | 0.010260779 | LMJF_24_0370, LMJF_13_1680, LMJF_03_0200, LMJF_35_0820, LMJF_04_0580, LMJF_12_0280 |
