# Supplementary material for: Essential gene prediction using limited gene essentiality information–An integrative semi-supervised machine learning strategy
Source: PLoS One. 2020 Nov 30;15(11):e0242943. doi: 10.1371/journal.pone.0242943 (PMC7703937; doi:10.1371/journal.pone.0242943)
Supplement: S1 Text — This supplementary text contains source code for the proposed machine learning strategy, including codes for (a) Training data set preparation and integration of heterogeneous features; (b) Feature selection based on the space-filling concept; (c) Dimension reduction using forced directed graph layout; (d) Semi-supervised classifier LapSVM. (DOCX) [file pone.0242943.s015.docx]

**S1 Text**

# Source Code of Proposed Machine learning strategy

The proposed pipeline has four following steps:-

1. Training data set preparation and integration of heterogeneous features [Script No: 1-25]
2. Feature selection based on the space-filling concept [Script No: 26]
3. Dimension reduction using forced directed graph layout [Script No: 27]
4. Semi-supervised classifier: LapSVM [Script No: 28]

Programming languages (e.g. PERL, MATLAB, R) are used to develop the proposed strategy. Script Number of each step is given in square bracket. Inputs for the pipeline are the genome-scale reconstructed metabolic networks, the fasta files containing the coding nucleotide sequences of the genes, protein sequences of these target organisms and limited gene essentiality information from experiment.

**#***********************************************************************************************

**Script No: 1**

**Script Name: derived_information_from_RN.m**

**Programming Language: MATLAB**

**Description: Fetching basic information(e.g. reactions name, genes name, and GPR relationship) from the reconstructed metabolic network**

**Input: Reconstructed Genome Scale Metabolic Model**

**Output: StochioMetric_Matrix.csv # Stoichiometric matrix**

**reactionname.csv # Reactions Name**

**genename.csv # Genes Name**

**GPR_Relation.csv # GPR relationship**

**#***********************************************************************************************

clc**;**

% Input: Reconstructed Genome Scale Metabolic Model

load**(**'<NewOrganismMetabolicModel>.mat'**);**

model**=<**NewOrganismMetabolicModel**>;**

Full**=**full**(**model**.**S**);**

a**=**char**(**model**.**rxns**);**

b**=**char**(**model**.**mets**);**

% Fetching Stoichiometric matrix from Reconstructed Genome Scale Metabolic Model

csvwrite**(**'StochioMetric_Matrix.csv'**,**model**.**S**)**

% Fetching Reactions Name from Reconstructed Genome Scale Metabolic Model

fid **=** fopen**(**'reactionname.csv'**,**'w'**);**

**for** row **=** 1**:**size**(**model**.**rxns**,**1**)**

fprintf**(**fid**,** '%s\n'**,**model**.**rxns**{**row**,**1**});**

**end**

fclose**(**fid**);**

% Fetching Genes Name from Reconstructed Genome Scale Metabolic Model

fid1 **=** fopen**(**'genename.csv'**,**'w'**);**

**for** row **=** 1**:**size**(**model**.**genes**,**1**)**

fprintf**(**fid1**,** '%s\n'**,**model**.**genes**{**row**,**1**});**

**end**

fclose**(**fid1**);**

% Fetching GPR relationship from Reconstructed Genome Scale Metabolic Model

fid2 **=** fopen**(**'GPR_Relation.csv'**,**'w'**);**

**for** row **=** 1**:**size**(**model**.**grRules**,**1**)**

fprintf**(**fid1**,** '%s\n'**,**model**.**grRules**{**row**,**1**});**

**end**

fclose**(**fid2**);**

**#*********************************************************************************

**Script No: 2**

**Script Name: construct_RN_for_Network_Analysis.pl**

**Programming Language: PERL**

**Description: Generate network file(net) for topological analysis**

**Input: StochioMetric_Matrix.csv # Stoichiometric matrix**

**reactionname.csv # Reaction Name**

**Exchanges.csv #Exchange reaction**

**Output: Reaction_Name_WO_Exchange.csv # Reactions Name without Exchanges**

**RN_Network.net # Reaction Network**

**#*********************************************************************************

**use** strict**;**

**use** warnings**;**

#--------------------Input Reaction Name----------------- **open(**FH2**,**"<reactionname.csv"**);**

**my** @reaction1**=**<FH2>**;**

#print "@reaction\n";

#-------Remove Exchange Reaction From Reaction Name----------

**open(**FH8**,**"<Exchanges.csv"**);**

**my** @reacd**=**<FH8>**;**

#-------------------------------------------------------------

**my** @index**;**

#--------------------Input Stochiometric Metric--------------

**open(**FH1**,**"<StochioMetric_Matrix.csv"**);**

**my** @sm1**=**<FH1>**;**

#--------------Input Stochiometric Metric----------------------

**open(**FH1**,**"<StochioMetric_Matrix.csv"**);**

**my** @sm**=**<FH1>**;**

#--------------------------------------------------------------

**my** @split_x**=split(**/,/**,**$sm1**[**0**]);**

**for(my** $i**=**0**;**$i**<**@split_x**;**$i**++){**

$index**[**$i**]=**0**;**

**}**

**for(my** $x**=**0**;**$x**<**@reacd**;**$x**++){**

**for(my** $x1**=**0**;**$x1**<**@reaction1**;**$x1**++)**

**{**

**chomp(**$reacd**[**$x**]);**

**chomp(**$reaction1**[**$x1**]);**

**if(**$reacd**[**$x**] eq** $reaction1**[**$x1**])**

**{**

$index**[**$x1**]=**1**;**

**}**

**}**

**}**

**my** $sindex**=**@index**;**

**open(**OUTS**,**">RSM.csv"**);**

**for(my** $x**=**0**;**$x**<**@sm**;**$x**++)**

**{**

**my** @split_x**=split(**/,/**,**$sm**[**$x**]);**

**for(my** $x1**=**0**;**$x1**<**@index**;**$x1**++)**

**{**

**if(**$index**[**$x1**]==**0**&&**$x1**<**$sindex**-**1**)**

**{**

**chomp(**$split_x**[**$x1**]);**

**print** OUTS "$split_x[$x1],"**;**

**}**

**if(**$index**[**$x1**]==**0**&&**$x1**==**$sindex**-**1**)**

**{**

**chomp(**$split_x**[**$x1**]);**

**print** OUTS "$split_x[$x1]"**;**

**}**

**}**

**print** OUTS "\n"**;**  **}**

**close(**OUTS**);**

#-----------------------------------------------

**open(**FH3**,**"<RSM.csv"**);**

**my** @s**=**<FH3>**;**

**my** $sr**=**@s**;**

#------------------------------------------------

**open(**OUT6**,**">Reaction_Name_WO_Exchange.csv"**);**

**for(my** $x2**=**0**;**$x2**<**@index**;**$x2**++)**

**{**

**if(**$index**[**$x2**]==**0**&&**$x2**<**$sindex**)**

**{**

**print** OUT6 "$reaction1[$x2]\n"**;**

**}**

**}**

**close(**OUT6**);**

#----------Output for .net file-----------------

**open(**OUT**,**">RN_Network.net"**);**

**my** @ss**;**

**my** $sz**;**

**for(my** $i**=**0**;**$i**<**$sr**;**$i**++)**

**{**

**chomp(**$s**[**$i**]);**

**my** @srow**=split(**/\,/**,**$s**[**$i**]);**

$sz**=**@srow**;**

**for(my** $j**=**0**;**$j**<**@srow**;**$j**++)**

**{**

$ss**[**$i**][**$j**]=**$srow**[**$j**];**

**}**

**}**

**my** @reacM**;**

**for(my** $i**=**0**;**$i**<**$sz**;**$i**++)**

**{**

**for(my** $j**=**0**;**$j**<**$sz**;**$j**++)**

**{**

$reacM**[**$i**][**$j**]=**0**;**

**}**

**}**

**for(my** $i**=**0**;**$i**<**$sz**;**$i**++)**

**{**

**for(my** $j**=**0**;**$j**<**$sr**;**$j**++)**

**{**

**if(**$ss**[**$j**][**$i**]!=**0**)**

**{**

**for(my** $k**=**0**;**$k**<**$sz**;**$k**++)**

**{**

**if(**$ss**[**$j**][**$k**]!=**0**)**

**{**

$reacM**[**$i**][**$k**]=**1**;**

**}**

**}**

**}**

**}**

**}**

#--------Input Coupled Reaction Matrix---------------

**open(**FH6**,**"<Reaction_Name_WO_Exchange.csv"**);**

**my** @reaction**=**<FH6>**;**

**my** $l**=**@reaction**;**

**print** "------------$l \n"**;**

**print** OUT "*Vertices $l\n"**;**

**for(my** $i**=**0**;**$i**<**$l**;**$i**++)**

**{**

**chomp(**$reaction**[**$i**]);**

**my** $ver**=**$i**+**1**;**

**print** OUT "$ver\t$reaction[$i]\n"**;**

**}**

**print** OUT "\n*Edges\n"**;**

#----------------------------------------------------

**for(my** $i**=**0**;**$i**<**$sz**;**$i**++)**

**{**

**for(my** $j**=**$i**+**1**;**$j**<**$sz**;**$j**++)**

**{**

**if(**$reacM**[**$i**][**$j**]!=**0**)**

**{**

**my** $v1**=**$i**+**1**;**

**my** $v2**=**$j**+**1**;**

**print** "$v1 $v2\n"**;**

**print** OUT "$v1\t$v2\n"**;**

**}**

**}**

**}**

**`rm RSM.csv`;**

**#*********************************************************************************

**Script No: 3**

**Script Name: Network_Analysis_of_RN.R**

**Programming Language: R**

**Description: Topological analysis from reaction network**

**Input: Reaction_Name_WO_Exchange.csv # Reactions Name without Exchanges**

**RN_Network.net # Reaction Network**

**Output: NF_RN.csv # Matrix with Topological Features of Reaction Network**

**#*********************************************************************************

#Topological features from reaction network

library**(**"igraph"**)**

g **<-** read_graph**(**'RN_Network.net', format **=** c**(** "pajek"**))**

reacname **=** read.csv**(**'Reaction_Name_WO_Exchange.csv'**)**

colnames**(**reacname**)<-**"reacname"

RN_degree**<-** degree**(**g**)**

evc**<-** evcent**(**g**)**

RN_eigen_value_centrality**=**evc**$**vector

RN_ecentricity**<-**eccentricity**(**g**)**

RN_hub_score **<-** hub_score**(**g, weights**=NA)$**vector

RN_authority_scores **<-** authority_score**(**g, weights**=NA)$**vector

RN_page_rank **<-** page.rank**(**g**)$**vector

RN_betweenness **<-**betweenness**(**g**)**

RN_number_of_triangle **<-** adjacent.triangles**(**g**)**

Reac_Name**<-**as.character**(**reacname**$**reacname**)**

node_stats_df **<-** cbind**(**Reac_Name,RN_degree,RN_eigen_value_centrality,RN_ecentricity,RN_hub_score,RN_aut hority_scores,RN_page_rank,RN_betweenness,RN_number_of_triangle**)**

write.csv**(**node_stats_df, file **=** "NF_RN.csv",quote **= FALSE**,eol **=** "\n",row.names**=FALSE)**

**#*********************************************************************************

**Script No: 4**

**Script Name: FCA_Analysis.m**

**Programming Language: MATLAB**

**Description: Flux coupling analysis (FCA)**

**Input: Reconstructed Genome Scale Metabolic Model**

**Output: Coupled_Reaction_Adjacency_Matrix.csv**

**Coupled_Reaction.csv #Coupled Reaction Name**

**#*********************************************************************************

%Using F2C2 for finding reactions that are coupled to each other

clc**;**

load**(**'<NewOrganismMetabolicModel>.mat'**);**

model**=**NewOrganismMetabolicModel**;**

Full**=**full**(**model**.**S**);**

a**=**char**(**model**.**rxns**);**

b**=**char**(**model**.**mets**);**

model2**=**struct**(**'stoichiometricMatrix'**,**Full**,**'reversibilityVector'**,**model**.**rev**,**'Reactions'**,** a**,**'Metabolites'**,**b**);**

**[**t1**,**t2**]=**F2C2**(**'glpk'**,** model2**);**

k**=**1**;**

**[**p**,**q**]=**size**(**t1**);**

**[**r**,**s**]=**size**(**t2**);**

**for** i**=**1**:**s

**if** t2**(**1**,**i**)==**0

reacname**(**k**,**1**)=**model**.**rxns**(**i**,**1**);** k**=**k**+**1**;**

**end**

**end**

f2c2var**=**t1**;**

**for** i**=**1**:**p

f2c2var**(**i**,**i**)=**0**;**

**end**

coupled**=**zeros**(**p**,**q**);**

**for** x**=**1**:**p

**for** y**=**1**:**p

**if** f2c2var**(**x**,**y**)~=**0

coupled**(**x**,**y**)=**1**;**

**end**

**end**

**end**

csvwrite**(**'Coupled_Reaction_Adjacency_Matrix.csv'**,**coupled**);**

fid **=** fopen**(**'Coupled_Reaction.csv'**,**'w'**);**

**for** row **=** 1**:**size**(**reacname**,**1**)**

fprintf**(**fid**,** '%s\n'**,**reacname**{**row**,**1**});**

**end**

fclose**(**fid**);**

**#**********************************************************************************

**Script No: 5**

**Script Name:** **construct_FCA_Network_for_Network_Analysis.pl**

**Programming Language: PERL**

**Description: Generate network file(net) for topological analysis**

**Input: Coupled_Reaction_Adjacency_Matrix.csv**

**Coupled_Reaction.csv**

**Output: FCA_Coupled_Network.net**

**#**********************************************************************************

**use** strict**;**

**use** warnings**;**

#--------Input Coupled Reaction Matrix-------------

**open(**FH1**,** "<Coupled_Reaction_Adjacency_Matrix.csv"**);**

**my** @coupled**=**<FH1>**;**

#------Input Reaction Matrix Name-----------------

**open(**FH2**,**"<Coupled_Reaction.csv"**);**

**my** @reaction**=**<FH2>**;**

#------Output for .net file-------------------------

**open(**OUT**,**">FCA_Coupled_Network.net"**);**

**my** $l**=**@coupled**;**

**print** OUT "*Vertices $l\n"**;**

**for(my** $i**=**0**;**$i**<**@reaction**;**$i**++)** **{**

**my** $ver**=**$i**+**1**;**

**print** OUT "$ver\t$reaction[$i]"**;** **}**

**print** OUT "\n*Edges\n"**;**

**for(my** $i**=**0**;**$i**<**@coupled**;**$i**++)** **{**

**chomp(**$coupled**[**$i**]);**

**my** @temp**=split(**/\,/**,**$coupled**[**$i**]);**

**for(my** $j**=**$i**;**$j**<**@temp**;**$j**++)** **{**

**if(**$temp**[**$j**]==**1**)**

**{**

**my** $v1**=**$i**+**1**;**

**my** $v2**=**$j**+**1**;**

**print** OUT "$v1\t$v2\n"**;**

**}**

**}**

**}**

**#*********************************************************************************

**Script No: 6**

**Script Name: Network_Analysis_of_FCA.R**

**Programming Language: R**

**Description: Topological analysis on Fluxed Coupled network**

**Input: FCA_Coupled_Network.net**

**Output: NF_FCA.csv # Matrix with Topological Features of FCA Network**

**#*********************************************************************************

library**(**"igraph"**)**

g **<-** read_graph**(**'FCA_Coupled_Network.net', format **=** c**(** "pajek"**))**

reacname **=** read.csv**(**'Coupled_Reaction.csv'**)**

colnames**(**reacname**)<-**"reacname"

FCA_degree**<-** degree**(**g**)**

evc**<-** evcent**(**g**)**

FCA_eigen_value_centrality**=**evc**$**vector

FCA_ecentricity**<-**eccentricity**(**g**)**

FCA_hub_score **<-** hub_score**(**g, weights**=NA)$**vector

FCA_authority_scores **<-** authority_score**(**g, weights**=NA)$**vector

FCA_page_rank **<-** page.rank**(**g**)$**vector

FCA_betweenness **<-**betweenness**(**g**)**

FCA_number_of_triangle **<-** adjacent.triangles**(**g**)**

Reac_Name**<-**as.character**(**reacname**$**reacname**)**

node_stats_df **<-** cbind**(**Reac_Name,FCA_degree,FCA_eigen_value_centrality,FCA_ecentricity,FCA_hub_score,FCA_authority_scores,FCA_page_rank,FCA_betweenness,FCA_number_of_triangle**)**

write.csv**(**node_stats_df, file **=** "NF_FCA.csv",quote **= FALSE**,eol **=** "\n",row.names**=FALSE)**

**#**********************************************************************************

**Script No: 7**

**Script Name: RXN_Gene_From_GPR.pl**

**Programming Language: PERL**

**Description: Generate reaction-gene combinations from GPR relation to integrate topological features and sequenced based features**

**Input: GPR_Relation.csv # GPR Relation from metabolic network**

**reactionname.csv #Reaction Name**

**Output: Reac_Gene.csv #** **Reaction-gene combinations**

**#**********************************************************************************

#!/usr/bin/perl

**use** strict**;**

**use** warnings**;**

**open(**OUT**,**">Reac_Gene.csv"**);**

**open(**FH1**,** "<reactionname.csv"**);**

**my** @reac**=**<FH1>**;**

**open(**FH2**,** "<GPR_Relation.csv"**);**

**my** @GPR**=**<FH2>**;**

**print** OUT "Reac_Gene\n"**;**

**for(my** $i**=**0**;**$i**<**@reac**;**$i**++)**

**{**

**chomp(**$reac**[**$i**]);**

**chomp(**$GPR**[**$i**]);**

**if(**$GPR**[**$i**] ne** ''**)**

**{**

$GPR**[**$i**] =~** tr/[or|and]\(\)|s+\r\n//d**;**

**print** "$GPR[$i]\n"**;**

**my** @temp1 **= split** ' '**,** $GPR**[**$i**];**

**my** $sz**=**@temp1**;**

**if(**$sz**>=**1**)**

**{**

**my** @temp3**;**

**for(my** $p**=**0**;**$p**<**@temp1**;**$p**++)**

**{**

**push(**@temp3**,**$temp1**[**$p**]);**

**}**

**my** %count**;**

**foreach my** $x **(**@temp3**)**

**{**

$count**{**$x**}++;**

**}**

**foreach my** $x **(keys** %count**)**

**{**

**print** OUT "$reac[$i]|$x\n"**;**

**}**

**}**

**}**

**}**

**#***********************************************************************************

**Script No: 8**

**Script Name: Process_nucleotide_sequence.pl**

**Programming Language: PERL**

**Description: Prepare nucleotide coding sequence with short header based on locus id of gene which are available in reconstructed metabolic network**

**Input: nucleotide_cds_from_genomic_new_organisms.fasta**

**Output:** **nucleotide_cds_RN_SH.fasta # FASATA file of nucleotide coding sequences with short header**

**nucleotide_cds_LH.fasta # FASATA file of nucleotide coding sequences with long header**

**#***********************************************************************************

#!/usr/bin/perl

**use** strict**;**

**use** warnings**;**

**open(**FH1**,** "<genename.csv"**);**

**my** @keytable**=**<FH1>**;**

**open(**FH2**,** "<nucleotide_cds_from_genomic_new_organisms.fasta"**);** #Input

**my** @file2**=**<FH2>**;**

**my** $sz**=**@file2**;**

**open(**OUT1**,**"> nucleotide_cds_RN_SH.fasta"**);**

**open(**OUT2**,**"> nucleotide_cds_LH.fasta"**);**

**open(**OUT3**,**">NOT_match_nucleotide_RN.fasta"**);**

**for(my** $k**=**0**;**$k**<**@keytable**;**$k**++)**

**{**

**print** "----------------$k+1 \n"**;**

$keytable**[**$k**] =~** s/^\s+//g**;**

$keytable**[**$k**] =~** s/\s+$//g**;**

**chomp(**$keytable**[**$k**]);**

**print** "$keytable[$k]\n"**;**

**my** $flag**=**0**;**

**my** $count**=**0**;**

**for(my** $i**=**0**;**$i**<**@file2**;)**

**{**

**my** $seqh1**;**

**my** $seqh2**;**

**my** $seq**;**

**if(**$file2**[**$i**]=~**/$keytable[$k]/**)**

**{**

**if((**$file2**[**$i**]=~**/protein_id\=$keytable[$k]/**) && (**$count**==**0**))**

**{**

**if(**$keytable**[**$k**] ne** ''**)**

**{**

$flag**=**1**;**

$count**=**$count**+**1**;**

**print** OUT2 $file2**[**$i**];**

**print** OUT1 ">$keytable[$k]\n"**;**

**while(**$file2**[**$i**+**1**]&&(**$i**+**1**)<**$sz**)**

**{**

**if((**$file2**[**$i**+**1**]=~**/\>/**))**

**{**

**last;**

**}**

**else**

**{**

$seq**.=**$file2**[**$i**+**1**];**

$i**++;**

**}**

**}**

$seq **=~** s/^\s+//**;**

$seq **=~** s/\s+$//**;**

**print** OUT1 "$seq\n"**;**

**print** OUT2 "$seq\n"**;**

**}**

**}**

**}**

$i**=**$i**+**1**;**

**}**

**if(**$flag**==**0**)**

**{**

**print** OUT3 "$keytable[$k],NA"**;**

**}**

**}**

**#**********************************************************************************

**Script No: 9**

**Script Name: Process_peptide_sequence.pl**

**Programming Language: PERL**

**Description: Prepare peptide sequence with short header based on locus id of gene which are available in reconstructed metabolic network**

**Input: protein_sequence_of_new_organisms.fasta**

**Output:** **Protein_RN_SH.fasta # FASATA file of protein sequences with short header**

**Protein_RN_LH.fasta # FASATA file of protein sequences with long header**

**#***********************************************************************************

#!/usr/bin/perl

**use** strict**;**

**use** warnings**;**

**open(**FH1**,** "<genename.csv"**);**

**my** @keytable**=**<FH1>**;**

**open(**FH2**,** "<protein_sequence_of_new_organisms.fasta"**);**

**my** @file2**=**<FH2>**;**

**my** $sz**=**@file2**;**

**open(**OUT1**,**">Protein_RN_SH.fasta"**);**

**open(**OUT2**,**">Protein_RN_LH.fasta"**);**

**open(**OUT3**,**">NOT_match_Protein_RN.fasta"**);**

**for(my** $k**=**0**;**$k**<**@keytable**;**$k**++)**  **{**

**print** "----------------$k+1 \n"**;**

$keytable**[**$k**] =~** s/^\s+//g**;**

$keytable**[**$k**] =~** s/\s+$//g**;**

#`sh release_share_memory.sh`;

**chomp(**$keytable**[**$k**]);**

**print** "$keytable[$k]\n"**;**

**my** $flag**=**0**;**

**my** $count**=**0**;**

**for(my** $i**=**0**;**$i**<**@file2**;)**

**{**

**my** $seqh1**;**

**my** $seqh2**;**

**my** $seq**;**

**if(**$file2**[**$i**]=~**/$keytable[$k]/**)**

**{**

**if((**$file2**[**$i**]=~**/$keytable[$k]/**) && (**$count**==**0**))**

**{**

**if(**$keytable**[**$k**] ne** ''**)** **{**

$flag**=**1**;**

$count**=**$count**+**1**;**

**print** OUT2 $file2**[**$i**];**

**print** OUT1 ">$keytable[$k]\n"**;**

**while(**$file2**[**$i**+**1**]&&(**$i**+**1**)<**$sz**)**

**{**

**if((**$file2**[**$i**+**1**]=~**/\>/**))**

**{**

**last;**

**}**

**else**

**{**

$seq**.=**$file2**[**$i**+**1**];**

$i**++;**

**}**

**}**

$seq **=~** s/^\s+//**;**

$seq **=~** s/\s+$//**;**

**print** OUT1 "$seq\n"**;**

**print** OUT2 "$seq\n"**;**

**}**

**}**

**}**

$i**=**$i**+**1**;**

**}**

**if(**$flag**==**0**)**

**{**

**print** OUT3 "$keytable[$k],NA"**;**

**}**

**}**

**#************************************************************************************

**Script No: 10**

**Script Name: Process_Ribosomal_Neucleotide.pl**

**Programming Language: PERL**

**Description: Prepare ribosomal sequence with short header based on locus id of gene**

**Input: nucleotide_cds_from_genomic_new_organisms.fasta**

**Output: nucleotide_cds_Ribosomal.fasta**

**#************************************************************************************

#!/usr/bin/perl

**use** strict**;**

**use** warnings**;**

**open(**FH2**,** "<nucleotide_cds_from_genomic_new_organisms.fasta"**);**

**my** @file2**=**<FH2>**;**

**my** $sz**=**@file2**;**

**open(**OUT2**,**"> nucleotide_cds_Ribosomal.fasta"**);**

**for(my** $i**=**0**;**$i**<**@file2**;)**

**{**

**my** $seq**;**

**if(**$file2**[**$i**]=~**/riboso/**)**

**{**

**print** OUT2 $file2**[**$i**];**

**while(**$file2**[**$i**+**1**]&&(**$i**+**1**)<**$sz**)**

**{**

**if((**$file2**[**$i**+**1**]=~**/\>/**))**

**{**

**last;**

**}**

**else**

**{**

$seq**.=**$file2**[**$i**+**1**];**

$i**++;**

**}**

**}**

**print** OUT2 "$seq"**;**

**}**

$i**=**$i**+**1**;**

**}**

**#**********************************************************************************

**Script No: 11**

**Script Name: CAI.pl**

**Programming Language: EMBOSS package and PERL**

**Description: Codon Adaptation Index(CAI) calculation from nucleotide coding sequences**

**Input: nucleotide_cds_Ribosomal.fasta**

**nucleotide_cds_RN_SH.fasta**

**Output:** **SF_CAI.csv**

**#**********************************************************************************

**#CAI Calculation from EMBOSS Package**

**`cusp -sequence <** **nucleotide_cds_Ribosomal.fasta> -outfile <Neucletide_cds_Ribosomal.cut>`;**

**`cai -seqall <** **nucleotide_cds_RN_SH.fasta> -cfile <** **nucleotide_cds_Ribosomal.cut > -outfile CAI.txt`;**

#!/usr/bin/perl

**use** strict**;**

**use** warnings**;**

**open(**FH2**,** "<CAI.txt"**);**

**open(**OUT**,** ">SF_CAI.csv"**);**

**my** @file2**=**<FH2>**;**

**print** OUT "GeneID,CAI\n"**;**

**for(my** $i**=**0**;**$i**<**@file2**;**$i**++)**

**{**

**chomp(**$file2**[**$i**]);**

$file2**[**$i**] =~** tr/[Sequence\:|CAI\:]//d**;**

**my** @temp**=split(**" "**,**$file2**[**$i**]);**

**print** OUT "$temp[0],$temp[1]\n"**;**

**}**

**#**********************************************************************************

**Script No: 12**

**Script Name: ENC.pl**

**Programming Language: EMBOSS package and PERL**

**Description: Effective Number of Codons (ENC) Calculation**

**Input: nucleotide_cds_RN_SH.fasta**

**Output: SF_ENC**

**#**************************************************************************

**`chips -seqall <** **nucleotide_cds_RN_SH.fasta> -sum No –outfile <OrganismName>.chips`;**

#!/usr/bin/perl

**use** strict**;**

**use** warnings**;**

**open(**FH2**,** "< OrganismName.chips"**);**

**open(**OUT**,** ">SF_ENC.csv"**);**

**my** @file2**=**<FH2>**;**

**print** OUT "GeneID,ENC\n"**;**

**for(my** $i**=**0**;**$i**<**@file2**;**$i**++)**

**{**

**chomp(**$file2**[**$i**]);**

**my** @temp**=split(**" "**,**$file2**[**$i**]);**

**print** OUT "$temp[0],$temp[3]\n"**;**

**}**

**#**********************************************************************************

**Script No: 13**

**Script Name: nucleotide_content.pl**

**Programming Language: PERL**

**Description: Frequencies of the A, T, G, and C nucleotides at the 3rd synonymous position of codons (A3, T3, G3, C3) of coding sequences**

**Input: nucleotide_cds_RN_SH.fasta**

**Output: SF_A3_G3_C3_T3.csv**

**#**********************************************************************************

#!/usr/bin/perl

**use** strict**;**

**use** warnings**;**

**open(**FH2**,** "< nucleotide_cds_RN_SH.fasta"**);**

**open(**OUT**,**">SF_A3_G3_C3_T3.csv"**);**

**print** OUT "GeneID,A3,T3,G3,C3\n"**;**

**my** @file2**=**<FH2>**;**

**my** $sz**=**@file2**;**

**my** $j**=**0**;**

**for(my** $i**=**0**;**$i**<**@file2**;)**

**{**

**my** $seqh**;**

**my** $seq**;**

**if(**$file2**[**$i**]=~**/\>/**)**

**{**

**chomp(**$file2**[**$i**]);**

$file2**[**$i**] =~** tr/\>//d**;**

$seqh**=**$file2**[**$i**];**

**print** OUT "$file2[$i],"**;**

**while(**$file2**[**$i**+**1**])**

**{**

**if((**$file2**[**$i**+**1**]=~**/\>/**))**

**{**

**last;**

**}**

**else**

**{**

$seq**.=**$file2**[**$i**+**1**];**

$i**++;**

**}**

**}**

**}**

$i**=**$i**+**1**;**

**chomp(**$seqh**);**

$seq **=~** tr/[B|D-F|H-S|U-Z|\*]|s+\r\n//d**;**

**my** @splitseq**=split(**""**,**$seq**);**

**my** $seq_sz**=**@splitseq**;** # Individual Sequence Size

#---------Calculation Of Sequence Length------------

**if(**$seq_sz**%**3**==**0**)**

**{**

**print** "$seqh,$seq_sz,Correct\n"**;**

**}**

**else**

**{**

**print** "$seqh,$seq_sz,Problem\n"**;**

**}**

#----------Calculation Of G3--------------------------

**my** $index**=**0**;**

**my** $count_A**=**0**;**

**my** $count_T**=**0**;**

**my** $count_G**=**0**;**

**my** $count_C**=**0**;**

**for(my** $i1**=**0**;**$i1**<**$seq_sz**;**$i1**=**$i1**+**3**)**

**{**

**for(my** $j1**=**0**;**$j1**<**3**&&**$index**<**$seq_sz**;**$j1**++)**

**{**

**if(**$j1**==**2**)**

**{**

**if(**$splitseq**[**$index**] eq** 'A'**)**

**{**

$count_A**++;**

**}**

**if(**$splitseq**[**$index**] eq** 'T'**)**

**{**

$count_T**++;**

**}**

**if(**$splitseq**[**$index**] eq** 'G'**)**

**{**

$count_G**++;**

**}**

**if(**$splitseq**[**$index**] eq** 'C'**)**

**{**

|  |  | $count_C**++;** |
| --- | --- | --- |
|  |  | **}** |
|  |  | **}** |
|  |  | $index**++;** |
|  |  | **}** |
|  |  | **}** |
|  |  | **printf** OUT "$count_A,$count_T,$count_G,$count_C\n"**;** |
|  |  | } |
|  | **}** |  |

**#**********************************************************************************

**Script No: 14**

**Script Name: Fre_20_Amino_Acids.pl**

**Programming Language: PERL**

**Description: Calculation frequencies of the twenty amino acids of each nucleotide sequence**

**Input: nucleotide_cds_RN_SH.fasta**

**Output:** **SF_Fre_20_Amino_Acids.csv**

**#**********************************************************************************

**`pepstats -sequence <** **nucleotide_cds_RN_SH.fasta> -outfile**

**<** **nucleotide_cds_RN_SH.pepstats>`;**

#!/usr/bin/perl

**use** strict**;**

**use** warnings**;**

**open(**FH1**,** "< nucleotide_cds_RN_SH.pepstats"**);**

**open(**OUT**,** ">SF_Fre_20_Amino_Acids.csv"**);**

**my** @file1**=**<FH1>**;**

**print** OUT "GeneId,Alanine,Cysteine,AsparticAcid,GlutamicAcid,Phenylalanine,Glycine,Histidine,Iso leucine,Lysine,Leucine,Asparagine,Proline,Glutamate,Arginine,Serine,Threonine,Valine,T ryptophan,Tyrosine,Methionine\n"**;**

**for(my** $i**=**0**;**$i**<**@file1**;**$i**++)**

**{**

| $file1**[**$i**] =~** s/\n//g**;** |  |
| --- | --- |
| $file1**[**$i**] =~** s/\r//g**;** |  |
| **chomp(**$file1**[**$i**]);** |  |
| **if(**$file1**[**$i**]=~**/^PEPSTATS.*/**)** |  |
| **{** |  |
| **my** @temp**=split(**" "**,**$file1**[**$i**]);** |  |
| **print** OUT "$temp[2],"**;** |  |
| **}** |  |
| **if(**$file1**[**$i**]=~**/^A\s+\=\s+Ala\s+(\d+).*/**)** | #Alanine |
| **{** |  |
| **chomp(**$1**);** |  |
| **print** OUT "$1,"**;** |  |
| **}** |  |
| **if(**$file1**[**$i**]=~**/^C\s+\=\s+Cys\s+(\d+).*/**)** | #Cysteine |
| **{** |  |
| **print** OUT "$1,"**;** |  |
| **}** |  |
| **if(**$file1**[**$i**]=~**/^D\s+\=\s+Asp\s+(\d+).*/**)** | #AsparticAcid |
| **{** |  |
| **print** OUT "$1,"**;** |  |
| **}** |  |
| **if(**$file1**[**$i**]=~**/^E\s+\=\s+Glu\s+(\d+).*/**)** | #GlutamicAcid |
| **{** |  |
| **print** OUT "$1,"**;** |  |
| **}** |  |
| **if(**$file1**[**$i**]=~**/^F\s+\=\s+Phe\s+(\d+).*/**)** | #Phenylalanine |
| **{** |  |
| **print** OUT "$1,"**;** |  |
| **}** |  |
| **if(**$file1**[**$i**]=~**/^G\s+\=\s+Gly\s+(\d+).*/**)** | #Glycine |
| **{** |  |
| **print** OUT "$1,"**;** |  |
| **}** |  |
| **if(**$file1**[**$i**]=~**/^H\s+\=\s+His\s+(\d+).*/**)** | #Histidine |
| **{** |  |
| **print** OUT "$1,"**;** |  |
| **}** |  |
| **if(**$file1**[**$i**]=~**/^I\s+\=\s+Ile\s+(\d+).*/**)** | #Isoleucine |
| **{** |  |
| **print** OUT "$1,"**;** |  |
| **}** |  |
| **if(**$file1**[**$i**]=~**/^K\s+\=\s+Lys\s+(\d+).*/**)** | #Lysine |
| **{** |  |
| **print** OUT "$1,"**;** |  |
| **}** |  |
| **if(**$file1**[**$i**]=~**/^L\s+\=\s+Leu\s+(\d+).*/**)** | #Leucine |
| **{** |  |
| **print** OUT "$1,"**;** |  |
| **}** |  |
| **if(**$file1**[**$i**]=~**/^N\s+\=\s+Asn\s+(\d+).*/**)** | #Asparagine |
| **{** |  |
| **print** OUT "$1,"**;** |  |
| **}** |  |
| **if(**$file1**[**$i**]=~**/^M\s+\=\s+Met\s+(\d+).*/**)** | #Methonine |
| **{** |  |
| **print** OUT "$1,"**;** |  |
| **}** |  |
| **if(**$file1**[**$i**]=~**/^P\s+\=\s+Pro\s+(\d+).*/**)** | #Proline |
| **{** |  |
| **print** OUT "$1,"**;** |  |
| **}** |  |
| **if(**$file1**[**$i**]=~**/^Q\s+\=\s+Gln\s+(\d+).*/**)** | #Glutamate |
| **{** |  |
| **print** OUT "$1,"**;** |  |
| **}** |  |
| **if(**$file1**[**$i**]=~**/^R\s+\=\s+Arg\s+(\d+).*/**)** | #Arginine |
| **{** |  |
| **print** OUT "$1,"**;** |  |
| **}** |  |
| **if(**$file1**[**$i**]=~**/^S\s+\=\s+Ser\s+(\d+).*/**)** | #Serine |
| **{** |  |
| **print** OUT "$1,"**;** |  |
| **}** |  |
| **if(**$file1**[**$i**]=~**/^T\s+\=\s+Thr\s+(\d+).*/**)** | #Threonine |
| **{** |  |
| **print** OUT "$1,"**;** |  |
| **}** |  |
| **if(**$file1**[**$i**]=~**/^V\s+\=\s+Val\s+(\d+).*/**)** | #Valine |
| **{** |  |
| **print** OUT "$1,"**;** |  |
| **}** |  |
| **if(**$file1**[**$i**]=~**/^W\s+\=\s+Trp\s+(\d+).*/**)** | #Tryptophan |
| **{** |  |
| **print** OUT "$1,"**;** |  |
| **}** |  |
| **if(**$file1**[**$i**]=~**/^Y\s+\=\s+Tyr\s+(\d+).*/**)** | #Tyrosine |
| **{** |  |
| **print** OUT "$1\n"**;** |  |
| **}** |  |
| **}** |  |

**#*********************************************************************************

**Script No: 15**

**Script Name: step15_IFC_pep_len.pl**

**Programming Language: PERL**

**Description: Calculation of Protein length**

**Input: nucleotide_cds_RN_SH.pepstats**

**Output: SF_pep_len.csv**

**#**********************************************************************************

**open(**F**,** "< nucleotide_cds_RN_SH.pepstats"**) or die;**

@data**=**<F>**;**

**open(**OUT**,**">SF_pep_len.csv"**);**

**print** OUT "GeneId,PL\n"**;**

**for(my** $i**=**0**;**$i**<**@data**;**$i**++)**

**{**

**if(**$data**[**$i**]=~**/PEPSTATS of/**)**

**{**

**my** @temp**=split(**' '**,**$data**[**$i**]);**

**print** OUT "$temp[2],"**;**

**}**

**if(**$data**[**$i**]=~**/^Molecular\s+\w+\s+\=\s+(\d+\.\d+)\s+\w+\s+\=\s+(\d+)/**)**

**{**

**print** OUT "$2\n"**;**

**}**

**}**

**close(FH);**

**#***********************************************************************************

**Script No: 16**

**Script Name: Paralogy_in_diff_E_value_cut_off.pl**

**Programming Language: PERL**

**Description: Calculation of Paralogy based features (Paralogy score)in different E-value cut-off**

**Input: Protein_RN_SH.fasta**

**Protein_ALL_SH.fasta**

**Output: SF_Paralog_P3_P5_P7_P10_P20_P30.csv**

**#***********************************************************************************

**`makeblastdb -in Protein_ALL_SH.fasta -input_type fasta -dbtype prot -parse_seqids**

**-out ALL -title "ALL"`;**

**`blastp -query Protein_RN_SH.fasta -db ALL -evalue 1e-3 -outfmt 6 -out blast_e_10_3.txt`;**

**`blastp -query Protein_RN_SH.fasta -db ALL -evalue 1e-5 -outfmt 6 -out blast_e_10_5.txt`;**

**`blastp -query Protein_RN_SH.fasta -db ALL -evalue 1e-7 -outfmt 6 -out blast_e_10_7.txt`;**

**`blastp -query Protein_RN_SH.fasta -db ALL -evalue 1e-10 -outfmt 6 -out blast_e_10_10.txt`;**

**`blastp -query Protein_RN_SH.fasta -db ALL -evalue 1e-20 -outfmt 6 -out blast_e_10_20.txt`;**

**`blastp -query Protein_RN_SH.fasta -db ALL -evalue 1e-30 -outfmt 6 -out blast_e_10_30.txt`;**

#-------------------------------------------------------------

**use** strict**;**

**use** warnings**;**

**my** @EV **= (**3**,**5**,**7**,**10**,**20**,**30**);**

**foreach my** $e **(**@EV**)**

**{**

**open(**F**,**"<blast_e_10_$e.txt"**);**

**my** @table**=**<F>**;**

**open(**OUT**,**">Paralog_E_10_$e.csv"**);**

**print** OUT "GeneID,P$e\n"**;**

**my** @data1**;**

**my** @data2**;**

**for(my** $i**=**0**;**$i**<**@table**;**$i**++)**

**{**

**chomp(**$table**[**$i**]);**

**my** @tempi**=split(**/\t/**,**$table**[**$i**]);**

**push(**@data1**,**$tempi**[**0**]);**

**push(**@data2**,**$tempi**[**1**]);**

**}**

**my** %count**;**

**foreach my** $x **(**@data1**)**

**{**

$count**{**$x**}++;**

**}**

**my** $i1**=**0**;**

**foreach my** $x **(keys** %count**)** **{**

**my** $P**=**0**;**

**my** @temp1**;**

**my** $c**=**0**;**

**for(my** $j**=**0**;**$j**<**@data1**;**$j**++)**

**{**

**if(**$x **eq** $data1**[**$j**])**  **{**

**if((**$x **eq** $data2**[**$j**]))** **{**

**my** @tempi1**=split(**/\t/**,**$table**[**$i1**]);**

**if(**$tempi1**[**2**]>**40**)**

**{**

$c**=**$c**+**1**;**

**}**

**}**

**}**

$P**=**$count**{**$x**}-**$c**;**

**}**

**print** OUT "$x,$P\n"**;**

$i1**=**$i1**+**1**;**

**}**

**}**

#-------------------------------------------------

**open(**F**,**"<Paralog_E_10_5.csv"**);**

**my** @KEY**=**<F>**;**

**open(**OUT**,**">SF_Paralog_P3_P5_P7_P10_P20_P30.csv"**);**

**print** OUT "GeneId,P3,P5,P7,P10,P20,P30\n"**;**

**open(**Pi3**,**"<Paralog_E_10_3.csv"**);**

**my** @P3**=**<Pi3>**;**

**open(**Pi5**,**"<Paralog_E_10_5.csv"**);**

**my** @P5**=**<Pi5>**;**

**open(**Pi7**,**"<Paralog_E_10_7.csv"**);**

**my** @P7**=**<Pi7>**;**

**open(**Pi10**,**"<Paralog_E_10_10.csv"**);**

**my** @P10**=**<Pi10>**;**

**open(**Pi20**,**"<Paralog_E_10_20.csv"**);**

**my** @P20**=**<Pi20>**;**

**open(**Pi30**,**"<Paralog_E_10_30.csv"**);**

**my** @P30**=**<Pi30>**;**

**for(my** $i**=**1**;**$i**<**@KEY**;**$i**++)**

**{**

**my** @temp_KEY**=split(**'\,'**,** $KEY**[**$i**]);**

$KEY**[**$i**]=**$temp_KEY**[**0**];**

**my** $flagP3**=**0**;**

**my** $flagP5**=**0**;**

**my** $flagP7**=**0**;**

**my** $flagP10**=**0**;**

**my** $flagP20**=**0**;**

**my** $flagP30**=**0**;**

**my** @splitP3**;**

**my** @splitP5**;**

**my** @splitP7**;**

**my** @splitP10**;**

**my** @splitP20**;**

**my** @splitP30**;**

$KEY**[**$i**]=~**s/\r\n//g**;**

**chomp(**$KEY**[**$i**]);**

**print** OUT "$KEY[$i]," **;**

**for(my** $j**=**1**;**$j**<**@P3**;**$j**++)**

**{**

**chomp(**$P3**[**$j**]);**

**if(**$P3**[**$j**]=~** /$KEY[$i]/**)**

**{**

@splitP3**=split(**'\,'**,**$P3**[**$j**]);**

$flagP3**=**1**;**

**}**

**}**

**for(my** $j**=**1**;**$j**<**@P5**;**$j**++)**

**{**

**chomp(**$P5**[**$j**]);**

**if(**$P5**[**$j**]=~** /$KEY[$i]/**)**

**{**

@splitP5**=split(**'\,'**,**$P5**[**$j**]);**

$flagP5**=**1**;**

**}**

**}**

**for(my** $j**=**1**;**$j**<**@P7**;**$j**++)**

**{**

**chomp(**$P7**[**$j**]);**

**if(**$P7**[**$j**]=~** /$KEY[$i]/**)**

**{**

@splitP7**=split(**'\,'**,**$P7**[**$j**]);**

$flagP7**=**1**;**

**}**

**}**

**for(my** $j**=**1**;**$j**<**@P10**;**$j**++)**

**{**

**chomp(**$P10**[**$j**]);**

**if(**$P10**[**$j**]=~** /$KEY[$i]/**)**

**{**

@splitP10**=split(**'\,'**,**$P10**[**$j**]);**

$flagP10**=**1**;**

**}**

**}**

**for(my** $j**=**1**;**$j**<**@P20**;**$j**++)**

**{**

**chomp(**$P20**[**$j**]);**

| **if(**$P20**[**$j**]=~** /$KEY[$i]/**)** |
| --- |
| **{** |
| @splitP20**=split(**'\,'**,**$P20**[**$j**]);** |
| $flagP20**=**1**;** |
| **}** |
| **}** |
| **for(my** $j**=**1**;**$j**<**@P30**;**$j**++)** |
| **{** |
| **chomp(**$P30**[**$j**]);** |
| **if(**$P30**[**$j**]=~** /$KEY[$i]/**)** |
| **{** |
| @splitP30**=split(**'\,'**,**$P30**[**$j**]);** |
| $flagP30**=**1**;** |
| **}** |
| **}** |
| **if(**$flagP3**==**1**)** |
| **{** |
| **print** OUT "$splitP3[1],"**;** |
| **}** |
| **else** |
| **{** |
| **print** OUT "0,"**;** |
| **}** |
| **if(**$flagP5**==**1**)** |
| **{** |
| **print** OUT "$splitP5[1],"**;** |
| **}** |
| **else** |
| **{** |
| **print** OUT "0,"**;** |
| **}** |
| **if(**$flagP7**==**1**)** |
| **{** |
| **print** OUT "$splitP7[1],"**;** |
| **}** |
| **else** |
| **{** |
| **print** OUT "0,"**;** |
| **}** |
| **if(**$flagP10**==**1**)** |
| **{** |
| **print** OUT "$splitP10[1],"**;** |
| **}** |
| **else** |
| **{** |
| **print** OUT "0,"**;** |
| **}** |
| **if(**$flagP20**==**1**)** |
| **{** |
| **print** OUT "$splitP20[1],"**;** |
| **}** |
| **else** |
| **{** |
| **print** OUT "0,"**;** |
| **}** |
| **if(**$flagP30**==**1**)** |
| **{** |
| **print** OUT "$splitP30[1]\n"**;** |
| **}** |
| **else** |
| **{** |
| **print** OUT "0\n"**;** |
| **}** |
| **}** |
|  |

**#**********************************************************************************

**Script No: 17**

**Script Name: CMI.pl**

**Programming Language: PERL**

**Description: Conditional Mutual Information (CMI) from nucleotide coding sequences**

**Input: nucleotide_cds_RN_SH.fasta**

**Output:** **SF_CMI.csv**

**#**********************************************************************************

#!/usr/bin/perl

**use** strict**;**

**use** warnings**;**

**use** Math**::**Complex**;**

**open(**FH2**,** "<nucleotide_cds_RN_SH.fasta"**);**

**open(**OUT**,**">SF_CMI.csv"**);**

**my** @ns**=(**"A"**,**"T"**,**"G"**,**"C"**);**

**my** %count**;**

# For Heade

**foreach my** $x **(**@ns**)**

**{**

$count**{**$x**}=**$x**;**

**}**

**my** $header**;**

**foreach my** $x **(sort keys** %count**)**

**{**

**foreach my** $y **(sort keys** %count**)** **{**

**foreach my** $z **(sort keys** %count**)**

**{**

**my** $te**=**$x**.**$y**.**$z**;**

$header**.=**"CMI\_$te,"**;** **}**

**}**

**}**

**chop(**$header**);**

**print** OUT "GeneID,"**.**"$header"**;**

**print** OUT "\n"**;**

**my** @file2**=**<FH2>**;**

**my** $sz**=**@file2**;**

**my** $j**=**0**;**

**for(my** $i**=**0**;**$i**<**@file2**;)**  **{**

**my** $seqh**;**

**my** $seq**;**

**if(**$file2**[**$i**]=~**/\>/**)**

**{**

**chomp(**$file2**[**$i**]);**

$file2**[**$i**] =~** tr/\>//d**;**

$seqh**=**$file2**[**$i**];**

**while(**$file2**[**$i**+**1**])**

**{**

**if((**$file2**[**$i**+**1**]=~**/\>/**))**

**{**

**last;**

**}**

**else**

**{**

$seq**.=**$file2**[**$i**+**1**];**

$i**++;**

**}**

**}**

**}**

$i**=**$i**+**1**;**

# Input Sequence fasta

**chomp(**$seqh**);**

$seq **=~** tr/[B|D-F|H-S|U-Z|\*]|s+\r\n//d**;**

# Calculation Of Conditional Mutual Information (CMI) features

**my** $ttnf**=**0**;**

**my** $tdnf**=**0**;**

**my** $tsnf**=**0**;**

**foreach my** $x **(sort keys** %count**)**

**{**

**foreach my** $y **(sort keys** %count**)** **{**

**foreach my** $z **(sort keys** %count**)** **{**

**my** $te**=**$x**.**$y**.**$z**;**

**my** $pxyz**;**

**if(**$pxyz **= (**$seq **=~** s/$te/$te/g**))**

**{**

$ttnf**=**$ttnf**+**$pxyz**;**

**}**

**}**

**}**

**}**

**print** "TTNF=$ttnf\n"**;**

# Total number of di neucleotide frequency count

**foreach my** $x **(sort keys** %count**)**

**{**

**foreach my** $y **(sort keys** %count**)** **{**

**my** $te**=**$x**.**$y**;**

**my** $pxy**;**

**if(**$pxy **= (**$seq **=~** s/$te/$te/g**))**

**{**

$tdnf**=**$tdnf**+**$pxy**;**

**}**

**}**

**}**

**print** "TDNF=$tdnf\n"**;**

# Total number of single neucleotide frequency count

**foreach my** $x **(sort keys** %count**)**

**{**

**my** $px**;**

**if(**$px **= (**$seq **=~** s/$x/$x/g**))** **{**

$tsnf**=**$tsnf**+**$px**;**

**}**

**}**

**print** "TSNF=$tsnf\n"**;**

**if(**$tsnf**%**3**==**0**)** #Check it is coding sequence or not?

**{**

**my** $result**=**$seqh**.**","**;**

**foreach my** $x **(sort keys** %count**)**

**{**

**foreach my** $y **(sort keys** %count**)**

**{**

**foreach my** $z **(sort keys** %count**)**

**{**

**my** $sin_z**=**$z**;**

**my** $din_xz**=**$x**.**$z**;** #di necleotide

**my** $din_yz**=**$y**.**$z**;** #di necleotide

**my** $tin_xyz**=**$x**.**$y**.**$z**;** #Tri necleotide

**my** $pz**;**

**if(**$pz **= (**$seq **=~** s/$sin_z/$sin_z/g**))** #Frequency count of z

**{**

**}**

**else**

**{**

$pz**=**0**;**

**}**

**my** $pxz**;**

**if(**$pxz **= (**$seq **=~** s/$din_xz/$din_xz/g**))** #Frequency count of xy

**{**

**}**

**else**

**{**

$pxz **=**0**;**

**}**

**my** $pyz**;**

**if(**$pyz **= (**$seq **=~** s/$din_yz/$din_yz/g**))** #Frequency count of xy

**{**

**}**

**else**

**{**

$pyz **=**0**;**

**}**

**my** $pxyz**;**

**if(**$pxyz **= (**$seq **=~** s/$tin_xyz/$tin_xyz/g**))** #Frequency count of xy

**{**

**}**

**Else**

|  | { |
| --- | --- |
|  | $pxyz =0; |
|  | } |
|  | #Mutual information formulae |
|  | if(((($pz/$tsnf)*($pxyz/$ttnf))/(($pxz/$tdnf)*($pyz/$tdnf)))>0) |
|  | { |
|  | my |
|  | $CMI_xyz=($pxyz/$ttnf)*logn(((($pz/$tsnf)*($pxyz/$ttnf))/(($pxz/$tdnf)*($pyz |
|  | /$tdnf))), 2); |
|  | $result.="$CMI_xyz,"; |
|  | } |
|  | else |
|  | { |
|  | $result.="0,"; |
|  | } |
|  | } |
|  | } |
|  | } |
|  | chop($result); |
|  | print OUT "$result\n"; |
|  | } # End of if $tsnf%3=0 |
| } |  |
|  |  |
|  |  |
|  |  |

**#*********************************************************************************

**Script No: 18**

**Script Name: MI.pl**

**Programming Language: PERL**

**Description: Mutual Information (MI) from nucleotide coding sequences**

**Input: nucleotide_cds_RN_SH.fasta**

**Output:** **SF_MI.csv**

**#*********************************************************************************

#!/usr/bin/perl

**use** strict**;**

**use** warnings**;**

**use** Math**::**Complex**;**

**open(**FH2**,** "<nucleotide_cds_RN_SH.fasta"**);**

**open(**OUT**,**">SF_MI.csv"**);**

**my** @ns**=(**"A"**,**"T"**,**"G"**,**"C"**);**

**my** %count**;**

# For Heade r

**foreach my** $x **(**@ns**)**

**{**

$count**{**$x**}=**$x**;** **}**

**my** $header**;**

**foreach my** $x **(sort keys** %count**)**

**{**

**foreach my** $y **(sort keys** %count**)**

**{**

**my** $te**=**$x**.**$y**;**

$header**.=**"MI\_$te,"**;**

**}**

**}**

**chop(**$header**);**

**print** OUT "GeneID,"**.**"$header"**;**

**print** OUT "\n"**;**

#-----------------------------------

**my** @file2**=**<FH2>**;**

**my** $sz**=**@file2**;**

**my** $j**=**0**;**

**for(my** $i**=**0**;**$i**<**@file2**;) {**

**my** $seqh**;**

**my** $seq**;**

**if(**$file2**[**$i**]=~**/\>/**)**

**{**

**chomp(**$file2**[**$i**]);**

$file2**[**$i**] =~** tr/\>//d**;**

$seqh**=**$file2**[**$i**];**

**while(**$file2**[**$i**+**1**])**

**{**

**if((**$file2**[**$i**+**1**]=~**/\>/**))**

**{**

**last;**

**}**

**else**

**{**

$seq**.=**$file2**[**$i**+**1**];**

$i**++;**

**}**

**}**

**}**

$i**=**$i**+**1**;**

# Input Sequence fasta

**chomp(**$seqh**);**

$seq **=~** tr/[B|D-F|H-S|U-Z|\*]|s+\r\n//d**;**

#----Calculation Of Mutual Information features--------

# Total number of di neucleotide frequency count

**my** $tdnf**=**0**;**

**my** $tsnf**=**0**;**

**foreach my** $x **(sort keys** %count**)** **{**

**foreach my** $y **(sort keys** %count**)** **{**

**my** $te**=**$x**.**$y**;**

**my** $pxy**;**

**if(**$pxy **= (**$seq **=~** s/$te/$te/g**))** **{**

$tdnf**=**$tdnf**+**$pxy**;**

**}**

**}**

**}**

# Total number of di neucleotide frequency count

**foreach my** $x **(sort keys** %count**)** **{**

**my** $px**;**

**if(**$px **= (**$seq **=~** s/$x/$x/g**))**

**{**

$tsnf**=**$tsnf**+**$px**;**

**}**

**}**

**if(**$tsnf**%**3**==**0**)** #Check it is coding sequence or not? **{**

**my** $result**=**$seqh**.**","**;** #Added gene ID

**foreach my** $x **(sort keys** %count**)**

**{**

**foreach my** $y **(sort keys** %count**)** **{**

**my** $te**=**$x**.**$y**;** #di necleotide

**my** $px**;**

**if(**$px **= (**$seq **=~** s/$x/$x/g**))** #Frequency count of x **{**

**}**

**else**

**{**

$px**=**0**;**

**}**

**my** $py**;**

**if(**$py **= (**$seq **=~** s/$y/$y/g**))** #Frequency count of y **{**

**}**

**else**

**{**

$py**=**0**;**

**}**

**my** $pxy**;**

**if(**$pxy **= (**$seq **=~** s/$te/$te/g**))** #Frequency count of xy

**{**

**}**

**else**

**{**

$pxy **=**0**;**

**}**

# Mutual information formulae

**if(((**$pxy**/**$tdnf**)/((**$px**/**$tsnf**)*(**$py**/**$tsnf**)))>**0**)**

**{**

**my** $MI_xy**=(**$pxy**/**$tdnf**)***logn**(((**$pxy**/**$tdnf**)/((**$px**/**$tsnf**)*(**$py**/**$tsnf**))),** 2**);**

$result**.=**"$MI_xy,"**;**

**}**

**else**

**{**

$result**.=**"0,"**;**

**}**

**}**

**}**

**chop(**$result**);**

**print** OUT "$result\n"**;**

**}** # End of if $tsnf%3=0

**}**

**#*********************************************************************************

**Script No: 19**

**Script Name: AverageKideraFactor.R**

**Programming Language: R**

**Description: Calculation of Average Kidera Factor**

**Input: protein sequences of each gene**

**Output: Average Kidera Factor**

**#*********************************************************************************

library**(**Peptides**)**

args **<-** commandArgs**(**trailingOnly**=TRUE)**

out**<-**kideraFactors**(**seq **=** args**)**

print**(**out**)**

**#**********************************************************************************

**Script No: 20**

**Script Name: AKF.pl**

**Programming Language: PERL**

**Description: Calculation of Average Kidera Factor**

**Input: Protein_sequences_of_new_organisms.fasta**

**Output: SF_KideraFactor.csv**

**#**********************************************************************************

#!/usr/bin/perl

**use** strict**;**

**use** warnings**;**

**open(**FH2**,** "<protein_sequences_of_new_organisms.fasta"**);**

**open(**OUT**,**">SF_KideraFactor.csv"**);**

**print** OUT "GeneID,AKF1,AKF2,AKF3,AKF4,AKF5,AKF6,AKF7,AKF8,AKF9,AKF10\n"**;**

**my** @file2**=**<FH2>**;**

**my** $sz**=**@file2**;** 9 **my** $j**=**0**;**

**my** $pc**=**1**;**

**for(my** $i**=**0**;**$i**<**@file2**;)** 12 **{**

**print** "-----------------$pc \n"**;**

$pc**=**$pc**+**1**;**

**my** $seqh**;**

**my** $seq**;**

**if(**$file2**[**$i**]=~**/\>/**)**

**{**

**chomp(**$file2**[**$i**]);**

$file2**[**$i**] =~** tr/\>//d**;**

$seqh**=**$file2**[**$i**];**

**print** OUT "$file2[$i],"**;**

**while(**$file2**[**$i**+**1**])**

**{**

**if((**$file2**[**$i**+**1**]=~**/\>/**))**

**{**

**last;**

**}**

**else**

**{**

$seq**.=**$file2**[**$i**+**1**];**

$i**++;**

**}**

**}**

**}**

$i**=**$i**+**1**;**

**chomp(**$seqh**);**

$seq **=~** tr/[B|D-F|H-S|U-Z|\*]|s+\r\n//d**;**

$seq **=~** s/\s+|\n|\r//g**;**

**my** $command **= `Rscript AverageKideraFactor.R** $seq**`;**

$command **=~** s/KF\d+//g**;**

**chomp(**$command**);**

**my** @array**=split** /\s+/**,**$command**;**

**print** OUT "$array[1],$array[2],$array[3],$array[4],$array[5],$array[6],$array[7],$array[8],$ar ray[9],$array[10]\n"**;**

**}**

**#*********************************************************************************

**Script No: 21**

**Script Name: FourierCoefficient_calculation.pl**

**Programming Language: PERL**

**Description: Calculation of Fourier sine and cosine coefficient using protein_sequences**

**Input: protein_sequences_of_new_organisms.fasta**

**Output:** **SF_SIN_FourierCoefficient.csv**

**SF_COS_FourierCoefficient.csv**

**#*********************************************************************************

#!/usr/bin/perl

**use** strict**;**

**use** warnings**;**

**use** Math**::**Complex**;**

**use** Math**::**Trig**;**

**open(**FH2**,** "<protein_sequences_of_new_organisms.fasta"**);**

**open(**OUT**,**">SF_SIN_FourierCoefficient.csv"**);**

**open(**OUT2**,**">SF_COS_FourierCoefficient.csv"**);**

#-----------For Fetch Kidera Table ---------------

**open(**FH**,**"<kftable.csv"**);**

**my** @file**=**<FH>**;**

**my** %hash**;**

**foreach my** $x**(**@file**)**

**{**

**chomp($x);**

**my** @temp**=split(**/\,/**,**$x**);**

**for(my** $i**=**1**;**$i**<**@temp**;**$i**++)**

**{**

**push(**@**{**$hash**{**$temp**[**0**]}},**$temp**[**$i**]);**

**}**

**}**

#--------------- For Heade -----------------------

**print** OUT "key,"**;**

**for(my** $k**=**1**;**$k**<=**7**;**$k**++)**#Wave number K

**{**

**for(my** $n**=**1**;** $n**<=**10**;** $n**++)** **{**

**if(**$k*****$n**!=**70**)**

**{**

**print** OUT "FCsin_WN$k\_KF$n,"**;**

**}**

**if(**$k*****$n**==**70**)**

**{**

**print** OUT "FCsin_WN$k\_KF$n\n"**;** **}**

**}**

**}**

**print** OUT2 "key,"**;**

**for(my** $k**=**0**;**$k**<=**7**;**$k**++)** #Wave number K **{**

**for(my** $n**=**1**;** $n**<=**10**;** $n**++)** **{**

**if(**$k*****$n**!=**70**)**

**{**

**print** OUT2 "FCcos_WN$k\_KF$n,"**;**

**}**

**if(**$k*****$n**==**70**)**

**{**

**print** OUT2 "FCcos_WN$k\_KF$n\n"**;**

**}**

**}**

**}**

# Break Necleotide Fasta

**my** @file2**=**<FH2>**;**

**my** $sz**=**@file2**;** **my** $j**=**0**;**

**for(my** $i**=**0**;**$i**<**@file2**;)**

**{**

**my** $seqh**;**

**my** $seq**;**

**if(**$file2**[**$i**]=~**/\>/**)**

**{**

**chomp(**$file2**[**$i**]);**

$file2**[**$i**] =~** tr/\>//d**;**

$seqh**=**$file2**[**$i**];**

**while(**$file2**[**$i**+**1**])**

**{**

**if((**$file2**[**$i**+**1**]=~**/\>/**))**

**{**

**last;**

**}**

**else**

**{**

$seq**.=**$file2**[**$i**+**1**];**

$i**++;**

**}**

**}**

**}**

$i**=**$i**+**1**;**

# Input Sequence fasta

**chomp(**$seqh**);**

$seq **=~** tr/\r\n//d**;**

#----Calculation Of Fourier Coefficient features----------

**my (**%sin_hash**,**%cos_hash**);**

**chomp(**$seq**);**

**my** @seq2**=split(**//**,**$seq**);**

**my** $len**=**@seq2**;**

**for(my** $k**=**0**;**$k**<=**7**;**$k**++)** #Wave number K **{**

**for(my** $n**=**0**;** $n**<**10**;** $n**++)** # 10 Kidera factor 91 **{**

**my** $count_sin**=**0**;**

**my** $count_cos**=**0**;**

**for(my** $l**=**0**;** $l**<**$len**;** $l**++)** **{**

$count_sin**=**$count_sin**+(**$hash**{**$seq2**[**$l**]}[**$n**]*sin((**2*****pi***$k***$l**)/**$len**));**

$count_cos**=**$count_cos**+(**$hash**{**$seq2**[**$l**]}[**$n**]*cos((**2*****pi***$k***$l**)/**$len**));**

**}**

$count_sin **= sprintf(**"%0.4f"**,** $count_sin**);**

$count_cos **= sprintf(**"%0.4f"**,** $count_cos**);** **if(**$k**>=**1**)**

**{**

**push(**@**{**$sin_hash**{**$seqh**}},**$count_sin**);**

**}**

**push(**@**{**$cos_hash**{**$seqh**}},**$count_cos**);**

**}**

**}**

#--------------Result Merge------------------------------------

**my** $resultx**;**

**my** $resulty**;**

**foreach my** $x**(keys** %sin_hash**)**

**{**

$resultx**=** $x**.**","**;**

**foreach my** $y**(**@**{**$sin_hash**{**$x**}})**

**{**

$resultx**.=**"$y,"**;**

**}**

**}**

**foreach my** $x**(keys** %cos_hash**)**

**{**

$resulty**=** $x**.**","**;**

**foreach my** $y**(**@**{**$cos_hash**{**$x**}})**

**{**

$resulty**.=**"$y,"**;**

**}**

**}**

**chop(**$resultx**);**

**chop(**$resulty**);**

**print** OUT "$resultx\n"**;**

**print** OUT2 "$resulty\n"**;**

**}**

**#*********************************************************************************

**Script No: 22**

**Script Name: merge_network_feature.pl**

**Programming Language: PERL**

**Description: Merge different network topological features**

**#*********************************************************************************

#!/usr/bin/perl

**use** strict**;**

**use** warnings**;**

#--------------------Command Line Argument ---------------------------

**my** $FH1_name**=**$ARGV**[**0**];** #Base File

**my** $FH2_name**=**$ARGV**[**1**];** #Result of Particular Feature Type

**my** $FH3_name**=**$ARGV**[**2**];** #Result will be merged with Base File 8

**open(**FH1**,** "<$FH1_name.csv"**);**

**my** @file1**=**<FH1>**;**

**open(**FH2**,** "<$FH2_name.csv"**);**

**my** @file2**=**<FH2>**;**

**open(**OUT**,** ">$FH3_name.csv"**);**

$file1**[**0**] =~** s/\r\n//g**;**

$file2**[**0**] =~** s/\r\n//g**;**

**chomp(**$file1**[**0**]);**

**my** @headerF**=split(**'\,'**,**$file2**[**0**],**2**);**

$headerF**[**1**]=~** s/\r\n//g**;**

**print** OUT "$file1[0],$headerF[1]"**;**

**for(my** $i**=**1**;**$i**<**@file1**;**$i**++)**

**{**

**chomp(**$file1**[**$i**]);**

$file1**[**$i**] =~** s/\r\n//g**;**

**my** @temp1**=split** '\|'**,** $file1**[**$i**];**

**my** $sz1**=**@temp1**;**

# print "$temp1[$sz1-1]\n";

**my** $flag**=**0**;**

**for(my** $j**=**1**;**$j**<**@file2**;**$j**++)**

**{**

$file2**[**$j**] =~** s/\r\n//g**;**

**if(**$file2**[**$j**]=~**/$temp1[0]/**)**

**{**

**my** @temp2 **= split** '\,'**,** $file2**[**$j**],**2**;**

**if(**$temp2**[**0**] eq** $temp1**[**0**])**

**{**

$temp2**[**1**] =~** s/\r\n//g**;**

**print** OUT "$file1[$i],$temp2[1]"**;**

$flag**=**1**;**

**}**

**}**

**}**

$file2**[**1**] =~** s/\r\n//g**;**

**my** @temp3 **= split** '\,'**,** $file2**[**1**];**

**my** $str**=**'0'**;**

**for(my** $k**=**0**;**$k**<**@temp3**-**2**;**$k**++)**

**{**

$str**.=**',0'**;**

**}**

**if(**$flag**==**0**)**

**{**

$file1**[**$i**] =~** s/\r\n//g**;**

**print** OUT "$file1[$i],$str\n"**;**

**}**

**}**

**#**********************************************************************************

**Script No: 23**

**Script Name: merge_sequence_feature.pl**

**Programming Language: PERL**

**Description: Merge different sequence features**

**#**********************************************************************************

#!/usr/bin/perl

**use** strict**;**

**use** warnings**;**

#--------------------Command Line Argument -----------------------------

**my** $FH1_name**=**$ARGV**[**0**];** #Base File

**my** $FH2_name**=**$ARGV**[**1**];** #Result of Particular Feature Type

**my** $FH3_name**=**$ARGV**[**2**];** #Result will be merged with Base File

**open(**FH1**,** "<$FH1_name.csv"**);**

**my** @file1**=**<FH1>**;**

**open(**FH2**,** "<$FH2_name.csv"**);**

**my** @file2**=**<FH2>**;**

**open(**OUT**,** ">$FH3_name.csv"**);**

**chomp(**$file1**[**0**]);**

**chomp(**$file2**[**0**]);**

**my** @headerF**=split(**'\,'**,**$file2**[**0**],**2**);**

**print** OUT "$file1[0],$headerF[1]\n"**;**

**for(my** $i**=**1**;**$i**<**@file1**;**$i**++)**

**{**

**chomp(**$file1**[**$i**]);**

**my** $flag**=**0**;**

**for(my** $j**=**1**;**$j**<**@file2**;**$j**++)**

**{**

**chomp(**$file2**[**$j**]);**

**my** @tempj**=split** '\,'**,** $file2**[**$j**];**

**if(**$file1**[**$i**]=~**/$tempj[0]/**)**

**{**

**my** @temp2 **= split** '\,'**,** $file2**[**$j**],**2**;**

**print** OUT "$file1[$i],$temp2[1]\n"**;**

$flag**=**1**;**

**}**

**}**

**chomp(**$file2**[**1**]);**

**my** @temp3 **= split** '\,'**,** $file2**[**1**];**

**my** $str**=**'0'**;**

**for(my** $k**=**0**;**$k**<**@temp3**-**2**;**$k**++)**

**{**

$str**.=**',0'**;**

**}**

**if(**$flag**==**0**)**

**{**

**print** OUT "$file1[$i],$str\n"**;**

**}**

**}**

**#********************************************************************************

**Script No: 24**

**Script Name: merge_gene_essentiality.pl**

**Programming Language: PERL**

**Description: Merge Gene Essentiality information with feature’s matrix**

**#********************************************************************************

#!/usr/bin/perl

**use** strict**;**

**use** warnings**;**

#--------------------Command Line Argument -----------------------------

**my** $FH1_name**=**$ARGV**[**0**];** #Base File

**my** $FH2_name**=**$ARGV**[**1**];** #Result of Particular Feature Type

**my** $FH3_name**=**$ARGV**[**2**];** #Result will be merged with Base File

**open(**FH1**,** "<$FH1_name.csv"**);**

**my** @file1**=**<FH1>**;**

**open(**FH2**,** "<$FH2_name.csv"**);**

**my** @file2**=**<FH2>**;**

**open(**OUT**,** ">$FH3_name.csv"**);**

**chomp(**$file1**[**0**]);**

**chomp(**$file2**[**0**]);**

**my** @headerF**=split(**'\,'**,**$file2**[**0**],**2**);**

**print** OUT "$file1[0],Class\n"**;**

**for(my** $i**=**1**;**$i**<**@file1**;**$i**++)**

**{**

**chomp(**$file1**[**$i**]);**

**my** $flag**=**0**;**

**for(my** $j**=**1**;**$j**<**@file2**;**$j**++)**

**{**

**chomp(**$file2**[**$j**]);**

**my** @tempj**=split** '\,'**,** $file2**[**$j**];**

**if(**$file1**[**$i**]=~**/$tempj[0]/**)**

**{**

**my** @temp2 **= split** '\,'**,** $file2**[**$j**],**2**;**

**print** OUT "$file1[$i],$temp2[1]\n"**;**

$flag**=**1**;**

**}**

**}**

**chomp(**$file2**[**1**]);**

**my** @temp3 **= split** '\,'**,** $file2**[**1**];**

**my** $str**=**'0'**;**

**for(my** $k**=**0**;**$k**<**@temp3**-**2**;**$k**++)**

**{**

$str**.=**',0'**;**

**}**

**if(**$flag**==**0**)**

**{**

**print** OUT "$file1[$i],$str\n"**;**

**}**

**}**

**#**********************************************************************************

**Script No: 25**

**Script Name: merge_ALL_feature.sh**

**Programming Language: SHELL Script**

**Description: Integration of all diverse features and generation of master training dataset**

**Input: Individual feature matrix**

**Output:** Master_Table_F289.csv #**Master Training Dataset with 289 features**

**#***********************************************************************************

**perl** merge_network_feature.pl Reac_Gene.csv NF_FCA Me_NF_FCA

**#Merge FCA features with Reaction Gene Pairs**

**perl** merge_network_feature.pl Me_NF_FCA NF_RN Me_NF_RN

**#Merge network topological features**

**perl** merge_sequence_feature.pl Me_NF_RN SF_CAI Me_SF_CAI

**#Merge CAI features**

**perl** merge_sequence_feature.pl Me_SF_CAI SF_ENC Me_SF_ENC

**#Merge ENC features**

**perl** merge_sequence_feature.pl Me_SF_ENC SF_A3_G3_C3_T3 Me_A3_G3_C3_T3

**#Merge nucleotide features**

**perl** merge_sequence_feature.pl Me_A3_G3_C3_T3 SF_Fre_20_Amino_Acids Me_SF_Fre_20_Amini_Acids

**#Merge frequency of Amino acids**

**perl** merge_sequence_feature.pl Me_SF_Fre_20_Amino_Acids SF_Pep_len Me_SF_Pep_len **#Merge frequency of peptide length**

**perl** merge_sequence_feature.pl Me_SF_Pep_len SF_Paralog_P3_P5_P7_P10_P20_P30 Me_Paralog_P3_P5_P7_P10_P20_P30 **#Merge frequency Paralogy Score**

**perl** merge_sequence_feature.pl Me_Paralog_P3_P5_P7_P10_P20_P30 SF_CMI Me_SF_CMI **#Merge CMI**

**perl** merge_sequence_feature.pl Me_SF_CMI SF_MI Me_SF_MI

**#Merge MI**

**perl** merge_sequence_feature.pl Me_SF_MI SF_KideraFactor Me_SF_KideraFactor

**#Merge Average Kidera Factor**

**perl** merge_sequence_feature.pl Me_SF_KideraFactor F_SIN_FourierCoefficient Me_SF_SIN_FourierCoefficient **#Merge sine Fourier Coefficient**

**perl** merge_sequence_feature.pl Me_SF_SIN_FourierCoefficient SF_COS_FourierCoefficient Me_SF_COS_FourierCoefficient

**#Merge cosine Fourier Coefficient**

**perl** merge_gene_essentiality.pl Me_SF_COS_FourierCoefficient GeneEssentiality Master_Table_F289

**#Merge limited gene essentiality information**

**#***************************************************************************

**Script No: 26**

**Script Name: UfsCov.R**

**Programming Language: R**

**Description: Selection of most relevant biological features from 289 features**

**Input:** Master_Table_F289.csv #Master training dataset

**Output:** **DataSet_After_UFS.csv #Data set after unsupervised feature selection**

**#***************************************************************************

#--------------------------------------------------------------------

#`Rscript --vanilla UfsCov.R <TrainingDataSetName>.csv

#------------Command Line Argument Passing --------------------------

args **=** commandArgs**(**trailingOnly**=TRUE)**

**if (**length**(**args**)<**1**) {**

stop**(**"Please Enter Dataset (input file).n", call.**=FALSE)**

**}else**

**{**

library**(**SFtools**)**

MT**<-**args**[**1**]**

inputcsv1**<-**read.csv**(**MT,head**=TRUE**,sep**=**","**)**

dat_unique **<-** unique**(**inputcsv1**)**

inp**=**dat_unique

fMBFS_SelecData**=**"DataSet_After_UFS.csv"

nCOL**=**NCOL**(**inp**)-**1

tryCatch**({**

print**(**"Process Start for Feature Selection"**)**

Results**<-** UfsCov_par**(**inp**[**2**:**nCOL**]**, ncores**=**10**)**

#-------------------Save Data matrix for selected features ------------

matr1**=**inp**[**,c**(**1,**(**Results**[[**2**]][**1**:**which.min**(**Results**[[**1**]])])+**1,NCOL**(**inp**))]**

write.csv**(**matr1,fMBFS_SelecData, row.names**=**F**)** 25

#----------------------------------------------------------------------

**}**, error**=function(**e**){**cat**(**"ERROR :",conditionMessage**(**e**)**, "\n"**)})** # TryCatch loop

**}**

**#***************************************************************************

**Script No: 27**

**Script Name: Select_K_Value_Circular_Data_structure.R**

**Programming Language: R**

**Description: After feature selection, the data set was transformed into a lower dimension (2-D) using a dimension reduction technique for visualization and finding best K for circular layout**

**Input: DataSet_After_UFS.csv #Dataset after feature selection**

**Output: Best K for circular layout after applying KK dimension reduction technique**

**#***************************************************************************

args **=** commandArgs**(**trailingOnly**=TRUE)**

**if (**length**(**args**)<**1**) {**

stop**(**"Please Enter Dataset (input file).n", call.**=FALSE)**

**}else**

**{**

library**(**dimRed**)**

library**(**doParallel**)**

#library(fossil)

library**(**plot3D**)**

library**(**ggplot2**)**

input**=**args**[**1**]**

#-----------------------------------------------------------------------

MasterTable**<-**read.csv**(**input,head**=TRUE**,sep**=**","**)**

Statics_h**=**paste**(**"K","Points_On_Circle","Points_Inside_Circle","Points_Outside_Circle", "%_of_Points_On_Circle",sep**=**","**)**

write.table**(**Statics_h, file**=**"DataSet_Statistics.csv", append**=**T, row.names**=**F, col.names**=**F, sep**=**",", quote**=FALSE)**

MTdata**<-**dimRedData**(**data **=** MasterTable**[**,(2**:**ncol**(**MasterTable**)-**1)**]**, meta **=**

MasterTable**[**ncol**(**MasterTable**)])**

no_cores **<-**10

registerDoParallel**(**cores**=**no_cores**)**

cl **<-** makeCluster**(**no_cores, type**=**"FORK"**)**

foreach**(**k**=** seq**(**from**=**2, to**=**10, by**=**1**)**,.errorhandling**=**"pass"**)** %dopar% **{**

tryCatch**({**

leim **<-** KamadaKawai**()** #Kamada Kawai

leim@stdpars**$**ndim**=**2

leim@stdpars**$**knn**=**k

emb **<-** leim@fun**(**MTdata, leim@stdpars**)**

emb@data@data**[**is.na**(**emb@data@data**)] <-** 0

MT_KK**=**cbind**(**MasterTable**[**1**]**,emb@data@data, MTdata@meta**)**

MT_KK**<-**MT_KK**[**order**(**MT_KK**[**,1**]**, decreasing **= FALSE)**,**]**

#---------Model Prediction for data set -----------

Radi_MT_KK_L1**<-**abs**(**sqrt**((**MT_KK**[**,2**]***MT_KK**[**,2**])+(**MT_KK**[**,3**]***MT_KK**[**,3**])))**

dm_MT_KK_eq_1**=**MT_KK**[**which**(**Radi_MT_KK_L1**==**1**)**,**]**

dm_MT_KK_lt_1**=**MT_KK**[**which**(**Radi_MT_KK_L1**<**1**)**,**]**

dm_MT_KK_gt_1**=**MT_KK**[**which**(**Radi_MT_KK_L1**>**1**)**,**]**

PD**=((**nrow**(**dm_MT_KK_eq_1**))/**nrow**(**MTdata**))***100 41

Statis**=**paste**(**k,nrow**(**dm_MT_KK_eq_1**)**,nrow**(**dm_MT_KK_lt_1**)**,nrow**(**dm_MT_KK_g t_1**)**,PD,sep**=**","**)**

write.table**(**Statis, file**=**"DataSet_Statistics.csv", append**=**T, row.names**=**F, col.names**=**F, sep**=**",", quote**=FALSE)**

**}**, error**=function(**e**){**cat**(**"ERROR :",conditionMessage**(**e**)**, "\n"**)})** # TryCatch loop

**}**#k

stopCluster**(**cl**)**

DataSet**<-**read.csv**(**"DataSet_Statistics.csv",head**=TRUE**,sep**=**","**)**

DataSet_sort**<-**DataSet**[**order**(**DataSet**[**,ncol**(**DataSet**)]**, decreasing **= FALSE)**,**]**

BestK**<-**DataSet_sort**[**1,1**]**

tryCatch**({**

leim **<-** KamadaKawai**()** #Kamada Kawai

leim@stdpars**$**ndim**=**2

leim@stdpars**$**knn**=**BestK

emb **<-** leim@fun**(**MTdata, leim@stdpars**)**

emb@data@data**[**is.na**(**emb@data@data**)] <-** 0

MT_KK**=**cbind**(**MasterTable**[**1**]**,emb@data@data, MTdata@meta**)**

MT_KK**<-**MT_KK**[**order**(**MT_KK**[**,1**]**, decreasing **= FALSE)**,**]**

| Radi_MT_KK_L1**<-**abs**(**sqrt**((**MT_KK**[**,2**]***MT_KK**[**,2**])+(**MT_KK**[**,3**]***MT_KK**[**,3**])))** |
| --- |
| dm_MT_KK_eq_1**=**MT_KK**[**which**(**Radi_MT_KK_L1**==**1**)**,**]** |
| dm_MT_KK_lt_1**=**MT_KK**[**which**(**Radi_MT_KK_L1**<**1**)**,**]** |
| RDM**=**rbind**(**dm_MT_KK_eq_1,dm_MT_KK_lt_1**)** |
| tiff**(**"Circular_Layout_Best_K", width **=** 6, height **=** 6, units **=** 'in', res **=** 600**)**  KK1**=**RDM**[**,2**]** |
| KK2**=**RDM**[**,3**]** |
| par**(**lwd**=**2,font.axis **=** 2**)** |
| #plot(x, x, col=ifelse(x==3, "red", "black")) |
| Class**=**as.integer**(**RDM**[**,4**])** |
| color **=** rep**(NA**, length**=**length**(**Class**))** |
| color**[**which**(**Class**==**1**)] =** "red" |
| color**[**which**(**Class**==**2**)] =** "Forest Green" |
| color**[**which**(**Class**==**3**)] =** "blue" |
| plot**(**KK1,KK2,col**=**color,type**=**"p",pch **=** 19,cex.lab**=**1,cex.main**=**1.25, cex.axis**=**2**)**  dev.off**()** |
| **}**, error**=function(**e**){**cat**(**"ERROR :",conditionMessage**(**e**)**, "\n"**)})** # TryCatch loop  **}** |

**#***************************************************************************

**Script No: 28**

**Script Name: KK_LapSVM.R**

**Programming Language: R**

**Description: Semi-supervised classifier (Laplacian SVM) and Best model selection by SSMSS Score**

**Input: DataSet_After_UFS.csv #Dataset after feature selection**

**Best K for circular layout after applying KK dimension reduction technique**

**Output: Prediction of essential reaction-gene pairs**

**#***************************************************************************

#`Rscript --vanilla KK_LapSVM_V4.R <TrainingDataSetName>.csv <Best K for Circular Layout>

#------------Command Line Argument Passing -----------------

args **=** commandArgs**(**trailingOnly**=TRUE)**

**if (**length**(**args**)<**2**) {**

stop**(**"Please Enter Training Dataset (input file).n", call.**=FALSE)**

**}else**

**{**

library**(**dplyr**)**

library**(**RSSL**)**

library**(**Matrix**)**

library**(**dimRed**)**

library**(**pdist**)**

library**(**clusterCrit**)**

library**(**ROCR**)**

# default output file

TrainData**<-**args**[**1**]**

MasterTable**<-**read.csv**(**TrainData,head**=TRUE**,sep**=**","**)**

k**=**as.integer**(**args**[**2**])** #$$$$$$$$$$$$$$$$$

print**(**k**)**

#---Function Defination of Supervised Performance Metrics------

performance_UD **<- function(**Result_MT**)**

**{**

P**=**0

TN**=**0

TP**=**0

PL**=**"E"

PN**=**"N"

PC**=**0

NC**=**0

Precision**=**0

Recall**=**0

F_measure**=**0

MCC**=**0

auROC**=**0

nr**=**nrow**(**Result_MT**)**

**for (**i1 **in** 1**:**nr**)**

**{**

**if(**identical**(**as.character**(**Result_MT**[**i1,2**])**,PL**))**

**{**

PC**=**PC**+**1

**if(**identical**(**as.character**(**Result_MT**[**i1,2**])**,as.character**(**Result_MT**[**i1,3**])))**

**{**

TP**=**TP**+**1

**}**

**}**

**if(**identical**(**as.character**(**Result_MT**[**i1,2**])**,PN**))**

**{**

NC**=**NC**+**1

**if(**identical**(**as.character**(**Result_MT**[**i1,2**])**,as.character**(**Result_MT**[**i1,3**])))**

**{**

TN**=**TN**+**1

**}**

**}**

**}**# End loop i1

PI**<-**length**(**which**(**as.character**(**Result_MT**[**,2**])==**"E"**))**

NI**<-**length**(**which**(**as.character**(**Result_MT**[**,2**])==**"N"**))**

Accuracy**=**0

TPR**=**0

FPR**=**0

TPR_W**=**0

FPR_W**=**0

TNR**=**0

FNR**=**0

Precision_W**=**0

Recall_W**=**0

F_measure_W**=**0

FN**=**PC**-**TP

FP**=**NC**-**TN

Accuracy**=(**TP**+**TN**)/(**TP**+**TN**+**FN**+**FP**)**

TPR**=**TP**/(**TP**+**FN**)**

TNR**=**TN**/(**TN**+**FP**)**

TPR_W**=((**TPR*****PI**)+(**TNR*****NI**))/(**PI**+**NI**)**

FPR**=**FP**/(**FP**+**TN**)**

FNR**=**FN**/(**FN**+**TP**)**

FPR_W**=((**FPR*****PI**)+(**FNR*****NI**))/(**PI**+**NI**)**

Precision_P**=**TP**/(**TP**+**FP**)**

Precision_N**=**TN**/(**TN**+**FN**)**

Precision_W**=((**Precision_P*****PI**)+(**Precision_N*****NI**))/(**PI**+**NI**)**

Recall_P**=**TP**/(**TP**+**FN**)**

Recall_N**=**TN**/(**TN**+**FP**)**

Recall_W**=((**Recall_P*****PI**)+(**Recall_N*****NI**))/(**PI**+**NI**)**

F_measure_P**=(**2***(**Precision_P*****Recall_P**))/(**Precision_P**+**Recall_P**)**

F_measure_N**=(**2***(**Precision_N*****Recall_N**))/(**Precision_N**+**Recall_N**)**

F_measure_W**=((**F_measure_P*****PI**)+(** F_measure_N*****NI**))/(**PI**+**NI**)**

MCC**=((**TP*****TN**)-(**FP*****FN**))/**sqrt**((**TP**+**FP**)*(**TP**+**FN**)*(**TN**+**FP**)*(**TN**+**FN**))**

act_pos**=**Result_MT**[**which**(**as.character**(**Result_MT**[**,2**])==**"E"**)**,2**]**

act_neg**=**Result_MT**[**which**(**as.character**(**Result_MT**[**,2**])==**"N"**)**,2**]**

pred_pos**=**Result_MT**[**which**(**as.character**(**Result_MT**[**,2**])==**"E"**)**,3**]**

pred_neg**=**Result_MT**[**which**(**as.character**(**Result_MT**[**,2**])==**"N"**)**,3**]**

actual**=**as.numeric**(**factor**(**rbind**(**act_pos,act_neg**)))**

predi**=**as.numeric**(**factor**(**rbind**(**pred_pos,pred_neg**)))**

pred **<-** prediction**(**predi,actual**)**

auc.tmp **<-** performance**(**pred,"auc"**)**

auROC **<-** as.numeric**(**auc.tmp@y.values**)**

TPR_W**[**is.infinite**(**TPR_W**)] <-** 0

FPR_W**[**is.infinite**(**FPR_W**)] <-** 0

Precision_W**[**is.infinite**(**Precision_W**)] <-** 0

Recall_W**[**is.infinite**(**Recall_W**)] <-** 0

F_measure_W**[**is.infinite**(**F_measure_W**)] <-** 0

MCC**[**is.infinite**(**MCC**)] <-** 0

Accuracy**[**is.infinite**(**Accuracy**)] <-** 0

auROC**[**is.infinite**(**auROC**)] <-** 0

TPR_W**[**is.nan**(**TPR_W**)] <-** 0

FPR_W**[**is.nan**(**FPR_W**)] <-** 0

Precision_W**[**is.nan**(**Precision_W**)] <-** 0

Recall_W**[**is.nan**(**Recall_W**)] <-** 0

F_measure_W**[**is.nan**(**F_measure_W**)] <-** 0

MCC**[**is.nan**(**MCC**)] <-** 0

Accuracy**[**is.nan**(**Accuracy**)] <-** 0

auROC**[**is.nan**(**auROC**)] <-** 0

my_list **<-**

list**(**"TP"**=**TP,"TN"**=**TN,"FP"**=**FP,"FN"**=**FN,"TPR_W"**=**TPR_W,"FPR_W"**=**FPR_W,"Precision_W"**=**Precisi

on_W,"Recall_W"**=**Recall_W,"F_measure_W"**=**F_measure_W, "MCC" **=** MCC, "auROC"**=**auROC, "Accuracy"**=**Accuracy**)**

return**(**my_list**)**

**}**

#-------------------------------------------------------------

fname**=**"ALL_Parameters.csv"

b_M_fname**=**"Best_Training_Model_Parameter.csv"

b_M_GEP**=**"Gene_Essentiality_Prediction_From_Best_Model.csv"

**if(**file.exists**(**fname**))**

**{**

unlink**(**fname**)**

**}**

**if(**file.exists**(**b_M_fname**))**

**{**

unlink**(**b_M_fname**)**

**}**

**if(**file.exists**(**b_M_GEP**))**

**{**

unlink**(**b_M_GEP**)**

**}**

header**=**paste**(**"k","rbp","gm","lm","kr1","TP","TN","FP","FN","TPR_W","FPR_W","Precision_ W","Recall_W","F_measure_W","MCC","auROC","Accuracy","SSMSS","Label",sep**=**","**)**

write.table**(** header, file**=**fname, append**=**T, row.names**=**F, col.names**=**F,sep**=**",", quote**=FALSE)**

write.table**(** header, file**=**b_M_fname, append**=**T, row.names**=**F, col.names**=**F,sep**=**",", quote**=FALSE)**

header_BMGEP**=**paste**(**"RXN_Gene","Experiment","Predicted",sep**=**","**)**

write.table**(** header_BMGEP, file**=**b_M_GEP, append**=**T, row.names**=**F, col.names**=**F, sep**=**",", quote**=FALSE)**

MTdata**<-**dimRedData**(**data **=** MasterTable**[**,2**:(**ncol**(**MasterTable**)-**1)**]**,

meta **=** MasterTable**[**ncol**(**MasterTable**)])**

tryCatch**({**

leim **<-** KamadaKawai**()** #Kamada Kawai

leim@stdpars**$**ndim**=**2

leim@stdpars**$**knn**=**k

emb **<-** leim@fun**(**MTdata, leim@stdpars**)**

emb@data@data**[**is.na**(**emb@data@data**)] <-** 0

MT_KK**=**cbind**(**MasterTable**[**1**]**,emb@data@data, MTdata@meta**)**

MT_KK**<-**MT_KK**[**order**(**MT_KK**[**,1**]**, decreasing **= FALSE)**,**]**

Class1**<-**MT_KK**[**,**(**ncol**(**MT_KK**))]**

MT_KK**=**cbind**(**MT_KK,Class1**)**

MT_KK **<-** MT_KK %>%

add_missinglabels_mar**(**Class1**~**.,prob**=**1**)**

#-------------LapSVM-------------------------

hSSMSSre**=**0

hsResult**=**0

hsPredictionClass**=**0

hsResult**=**paste**(**0,0,0,0,0,0,0,0,0,0,0,0,0,0,0,0,0,0,0,0,0,0,0,0,sep**=**""**)**

MT_KK_i**=**MT_KK

nr**=**nrow**(**MT_KK_i**)**

LD_E**=**MT_KK_i**[**which**(**MT_KK_i**[**,ncol**(**MT_KK_i**)]==**"E"**)**,2**]**

LD_N**=**MT_KK_i**[**which**(**MT_KK_i**[**,ncol**(**MT_KK_i**)]==**"N"**)**,2**]**

iranS**=**length**(**LD_E**)+**length**(**LD_N**)**

MT_KK_i**[**which**(**as.character**(**MT_KK_i**[**,2**])** %in% LD_E**)**,1**]=**"E"

MT_KK_i**[**which**(**as.character**(**MT_KK_i**[**,2**])** %in% LD_N**)**,1**]=**"N"

dm_Laptrain**<-**MT_KK_i**[**order**(**MT_KK_i**[**,1**]**, decreasing **= FALSE)**,**]**

dm_Laptrain1**<-**dm_Laptrain**[**,c**(**2,3**:**4,1,ncol**(**dm_Laptrain**))]**

dm_Laptrain1_L_E**=**dm_Laptrain1**[**which**(**dm_Laptrain1**[**,4**]==**'E'**)**,**]**

dm_Laptrain1_L_N**=**dm_Laptrain1**[**which**(**dm_Laptrain1**[**,4**]==**'N'**)**,**]**

dm_Laptrain1_UL**=**dm_Laptrain1**[-**which**((**dm_Laptrain1**[**,4**]==**'E'**)|(**dm_Laptrain1**[**,4**]==**'N'

**))**,**]**

Radi_L1**<-**abs**(**sqrt**((**dm_Laptrain1_UL**[**,2**]***dm_Laptrain1_UL**[**,2**])+(**dm_Laptrain1_UL**[**,3**]***d m_Laptrain1_UL**[**,3**])))**

dm_Laptrain1_UL_eq_1**=**dm_Laptrain1_UL**[**which**(**Radi_L1**<=**1**)**,**]**

disEtoE**<-**as.matrix**(**pdist**(**dm_Laptrain1_L_E**[**,2**:**3**]**,dm_Laptrain1_L_E**[**,2**:**3**]))**

disNtoN**<-**as.matrix**(**pdist**(**dm_Laptrain1_L_N**[**,2**:**3**]**,dm_Laptrain1_L_N**[**,2**:**3**]))**

UT_disEtoE**<-**disEtoE**[**upper.tri**(**disEtoE, diag **= FALSE)]**

UT_disNtoN**<-**disNtoN**[**upper.tri**(**disNtoN, diag **= FALSE)]**

avgDisE**<-**sum**(**UT_disEtoE**)/**length**(**UT_disEtoE**)**

avgDisN**<-**sum**(**UT_disNtoN**)/**length**(**UT_disNtoN**)**

avgDisEN**= (**avgDisE**+**avgDisN**)/**2

disEtoUL**<-**as.matrix**(**pdist**(**dm_Laptrain1_L_E**[**,2**:**3**]**,dm_Laptrain1_UL_eq_1**[**,2**:**3**]))**

disNtoUL**<-**as.matrix**(**pdist**(**dm_Laptrain1_L_N**[**,2**:**3**]**,dm_Laptrain1_UL_eq_1**[**,2**:**3**]))**

NC**<-**ncol**(**dm_Laptrain1_UL**)**

averageDisEtoUL**<-**colMeans**(**disEtoUL**)**

averageDisNtoUL**<-**colMeans**(**disNtoUL**)**

**if(**avgDisE**<**avgDisN**)**

**{**

dm_Laptrain1_UL_eq_1**[**which**(**averageDisEtoUL**<(**avgDisE**))**,NC**+**1**]=**"E"

dm_Laptrain1_UL_eq_1**[**which**(**averageDisEtoUL**>=(**avgDisE**))**,NC**+**1**]=**"N"

**}**

**if(**avgDisE**>**avgDisN**)**

**{**

dm_Laptrain1_UL_eq_1**[**which**(**averageDisEtoUL**<(**avgDisN**))**,NC**+**1**]=**"E"

dm_Laptrain1_UL_eq_1**[**which**(**averageDisEtoUL**>=(**avgDisN**))**,NC**+**1**]=**"N"

**}**

dm_Laptrain1_L_E**[**,NC**+**1**]=**"E"

dm_Laptrain1_L_N**[**,NC**+**1**]=**"N"

dm_Laptrain3**=**rbind**(**dm_Laptrain1_L_E,dm_Laptrain1_L_N,dm_Laptrain1_UL_eq_1**)**

dm_Laptrain4**=**dm_Laptrain3

rbfp**=**c**(**0.01,0.1,1**)**

**for (**rbp **in** rbfp**)**

**{**

rbf_param **<-** rbp

krbf**=**kernlab**::**rbfdot**(**rbf_param**)**

kr1**=**1

gmA**=**c**(**0.01,0.1,1**)**

**for (**gm **in** gmA**)**

**{**

lmA**=**c**(**0.01,0.1,1**)**

**for (**lm **in** lmA**)**

**{**

print**(**paste**(**iranS,"---",rbp,"---",lm,"---",gm,sep**=**""**))**

Class**<-** factor**(**dm_Laptrain4**[**,4**])**

lap_svm **<-**LaplacianSVM**(**Class**~**.,dm_Laptrain4**[**,2**:**3**]**,scale**=**T,kernel**=**krbf

,lambda**=**lm,gamma**=**gm,eps**=**1e**-**09,adjacency_k **=** k,normalized_laplacian **= FALSE)**

#--------------------------------------------------------------------

prediction_Train **<-**  predict**(**lap_svm,dm_Laptrain4**[**,2**:**3**])**

**if(**any**(!**is.na**(**prediction_Train**)))**

**{**

RM_Pred_Train**<-**cbind**(**dm_Laptrain4,prediction_Train**)**

Result_Train_L**<-**RM_Pred_Train**[**which**((**RM_Pred_Train**[**,4**]==**'E'**)|(**RM_Pre d_Train**[**,4**]==**'N'**))**,c**(**1,4,ncol**(**RM_Pred_Train**))]**

Result_Train**<-**cbind**(**as.character**(**Result_Train_L**[**,1**])**,as.character**(**fa ctor**(**Result_Train_L**[**,2**]))**,as.character**(**factor**(**Result_Train_L**[**,3**])))**

per_Train_L **<-** performance_UD**(**Result_Train**)**

R1_train_L**=**paste**(**k,gm,lm,kr1,per_Train_L**$**MCC,per_Train_L**$**TP,per_Trai n_L**$**TN,per_Train_L**$**FP,per_Train_L**$**FN,sep**=**","**)**

#----------------------------------------------------

dm_Train**<-**dm_Laptrain4

prediction_Train **<-** predict**(**lap_svm, dm_Train**[**,2**:**3**])**

Result_Train**<-**cbind**(**as.character**(**dm_Train**[**,1**])**,as.character**(**factor**(**d m_Train**[**,6**]))**,as.character**(**factor**(**prediction_Train**)))**

per_Train **<-**

performance_UD**(**Result_Train**)**

DM_MT**=**as.matrix**(**dm_Train**[**,2**:**3**])**

CL_MT_LR**=**as.integer**(**factor**(**prediction_Train**))**

UnSup_Inx_LR **<-** intCriteria**(**DM_MT,CL_MT_LR,"all"**)**

CL_MT_PR**=**as.integer**(**factor**(**dm_Train**[**,6**]))**

UnSup_Inx_PR **<-** intCriteria**(**DM_MT,CL_MT_PR,"all"**)**

#---------------------------------------------------------

Result_MT_GT**<-**cbind**(**as.character**(**dm_Train**[**,1**])**,as.character**(**factor**(**d m_Train**[**,5**]))**,as.character**(**factor**(**prediction_Train**)))**

per_MT_GT **<-** performance_UD**(**Result_MT_GT**)**

UnSup_Inx_LR**$**silhouette**[**is.infinite**(**UnSup_Inx_LR**$**silhouette**)] <-** 0

UnSup_Inx_PR**$**silhouette**[**is.infinite**(**UnSup_Inx_PR**$**silhouette**)] <-** 0

per_Train_L**$**MCC**[**is.infinite**(**per_Train_L**$**MCC**)] <-** 0

per_Train**$**MCC**[**is.infinite**(**per_Train**$**MCC**)] <-** 0

UnSup_Inx_LR**$**silhouette**[**is.nan**(**UnSup_Inx_LR**$**silhouette**)] <-** 0

UnSup_Inx_PR**$**silhouette**[**is.nan**(**UnSup_Inx_PR**$**silhouette**)] <-** 0

per_Train_L**$**MCC**[**is.nan**(**per_Train_L**$**MCC**)] <-** 0

per_Train**$**MCC**[**is.nan**(**per_Train**$**MCC**)] <-** 0

UnSup_Inx_LR**$**silhouette**[**is.na**(**UnSup_Inx_LR**$**silhouette**)] <-** 0

UnSup_Inx_PR**$**silhouette**[**is.na**(**UnSup_Inx_PR**$**silhouette**)] <-** 0

per_Train_L**$**MCC**[**is.na**(**per_Train_L**$**MCC**)] <-** 0

per_Train**$**MCC**[**is.na**(**per_Train**$**MCC**)] <-** 0

#-------------------------SSMSS Calculation -----------------------

**if((**per_Train_L**$**MCC**>=**0**)&(**per_Train**$**MCC**>=**0**)&(**UnSup_Inx_LR**$**silhouette**>**

**=**0**)&(**UnSup_Inx_PR**$**silhouette**>=**0**))**

**{**

SSMSS**=min(**UnSup_Inx_LR**$**silhouette**,**UnSup_Inx_PR**$**silhouette**,**per_Train_L**$**MCC*****per_Train**$**MCC**)**

SSMSS_V**=**paste**(**k,rbp,gm,lm,kr1,per_MT_GT**$**TP,per_MT_GT**$**TN,per_MT_GT

**$**FP,per_MT_GT**$**FN,per_MT_GT**$**TPR_W,per_MT_GT**$**FPR_W,per_MT_GT**$**Precis ion_W,per_MT_GT**$**Recall_W,per_MT_GT**$**F_measure_W,per_MT_GT**$**MCC,per_ MT_GT**$**auROC,per_MT_GT**$**Accuracy,SSMSS,iranS,sep**=**","**)**

write.table**(** SSMSS_V, file**=**fname, append**=**T, row.names**=**F, col.names**=**F, sep**=**",", quote**=FALSE)**

**}** #if(per_Train_L$Accuracy>0 & intIdx_MT_GT$silhouette>0 & intIdx_MT$silhouette>0)

**}**

**if(**SSMSS**>=**hSSMSSre**)**

**{**

hSSMSSre**=**SSMSS

hsResult**=**SSMSS_V

bmPredictionClass**=**Result_MT_GT

**}**

**}** #lm

**}** #gm

kr1**=**kr1**+**1

**}**#for (rbp in kerA)

write.table**(**hsResult, file**=**b_M_fname, append**=**T, row.names**=**F, col.names**=**F,sep**=**",", quote**=FALSE)**

write.table**(**bmPredictionClass, file**=**b_M_GEP, append**=**T, row.names**=**F, col.names**=**F, sep**=**",", quote**=FALSE)**

**}**, error**=function(**e**){**cat**(**"ERROR :",conditionMessage**(**e**)**, "\n"**)})** # TryCatch loop

**}**
