# Supplementary material for: Essential gene prediction using limited gene essentiality information–An integrative semi-supervised machine learning strategy
Source: PLoS One. 2020 Nov 30;15(11):e0242943. doi: 10.1371/journal.pone.0242943 (PMC7703937; doi:10.1371/journal.pone.0242943)
Supplement: Attachment — Submitted filename: Response to Reviewers.pdf [file pone.0242943.s017.pdf]

<u>Response to Reviewers</u>

To,

Dr. Seyedali Mirjalili

Academic Editor

PLOS ONE

Dear Dr. Mirjalili,

First, we would like to sincerely thank you for giving us another opportunity to perform the revision of our manuscript. We also thank the Reviewers for their useful suggestions and recognizing the importance of our work and effort. The manuscript has now been revised further following the suggestions/comments made by the third Reviewer (**Highlighted in the revised Manuscript, Marked-up Copy**), and the point to point answers are given below.

We hope this revised version will satisfy you and the reviewers and will be acceptable to the journal.

REPLY TO REVIEWERS' COMMENTS

Reviewer #1: My comments have been addressed.

Reply: We thank the Reviewer for appreciating our efforts.

==

Reviewer #3: In this paper, the authors proposed the machine learning pipeline for essential gene prediction. This is a good work. The quality of the paper has been improved after revision. There are several observations listed as follows:

Reply: We thank the Reviewer for the critical review and the useful suggestions. In the revised version of the manuscript, we have now tried to answer the queries raised by the Reviewer to enhance the clarity of our manuscript.

Comment 1: Please separate the Introduction into two sections (1) Introduction and (2) Related Works.

Reply: We thank the reviewer for the useful suggestion. We admit that the current length of the introduction is a bit on the higher side. However, the manuscript organization according to the Plos One format does not accommodate any separate section for Related Works. This might affect the flow of the introduction for the readers and might break its continuity with the subsequent sections. Hence, we have retained the introduction in its current form. However, if the editorial board finds it appropriate and give permission, we are willing to change the format for the introduction section, if required.

Comment 2: Authors performed the feature selection and then used the dimensionality

reduction method. Why use both feature selection and dimensionality reduction techniques in the same model? You can reduce a great number of features with only feature selection or dimensionality reduction. The justification for using both technique is required.

Reply: Both the feature selection and the dimensionality reduction methods are used for reducing the number of features in a dataset. Feature selection is also used for selecting the relevant features without changing the original values, whereas, the dimensionality reduction step transforms the higher dimensional features into a lower dimension. It is very difficult to identify the key features from the dimension reduction technique which are contributing for classification.
From our analysis (Fig. 3), we observe that in our pipeline, the Dimension Reduction technique Kamada Kawai is contributing heavily towards enhancing the model performance for all the organisms. However, we have also retained the Feature Selection step to identify the important features that are contributing to gene essentiality classification.
The corresponding results have also been discussed in the current revised version of the manuscript (Revised Manuscript, Lines #348-354,719-721, Highlighted)

Comment 3: In Figure 2, the results for the training and testing accuracy are similar in most cases. Have you run the experiment for several times (with different data partition) and then take the average?

Reply: We apologize for the lack of clarity of the Figure 2 which has arisen due to the scaling of the Y-axis. In Figure 2, although the accuracy for the training and testing looks similar in most cases, the values are not exactly the same.

To establish the model consistency and reproducibility, the model has been run by creating different data sets in each labeled/partition category (e.g. 1% category, where 100 datasets of 1% labelled category were created randomly from the master table). It is to be mentioned here that this selection of labeled data was conditionally randomized to ensure that both the essential and non-essential genes categories appear with equal probability. Thereafter the model performance of these 100 models were represented by plotting the average and standard deviation of each metrics. As observed from the low standard deviations for each metrics (under each category), it is worth to mention that the accuracy for the training and testing are very similar in most of the cases.

It is to be noted that 100 datasets were created for each of the 10 partition categories (1%, 2%, 3%, 4%, 5%, 10%, 30%, 50%, 70% and 90%). Hence the total number of datasets for each organism for training was 100x10=1000 datasets. (Revised Manuscript, Line # 523-529). We hope this answers the concern raised by the Reviewer.

We hope that this revised version will satisfy the reviewers and will be acceptable for publication in PLoS ONE
